# The U-box family genes in *Medicago truncatula*: Key elements in response to salt, cold, and drought stresses

**DOI:** 10.1371/journal.pone.0182402

**Published:** 2017-08-03

**Authors:** Jianbo Song, Xiaowei Mo, Haiqi Yang, Luming Yue, Jun Song, Beixin Mo

**Affiliations:** 1 Guangdong Provincial Key Laboratory for Plant Epigenetics, College of Life Sciences and Oceanography, Shenzhen University, Shenzhen, China; 2 Department of Biochemistry and Molecular Biology, College of Science, Jiang Xi Agricultural University, Nanchang, China; 3 Key Laboratory of Optoelectronic Devices and Systems of Ministry of Education and Guangdong Province, College of Optoelectronic Engineering, Shenzhen University, Shenzhen, China; Nanjing Agricultural University, CHINA

## Abstract

The ubiquitination pathway regulates growth, development, and stress responses in plants, and the U-box protein family of ubiquitin ligases has important roles in this pathway. Here, 64 putative U-box proteins were identified in the *Medicago truncatula* genome. In addition to the conserved U-box motif, other functional domains, such as the ARM, kinase, KAP, and WD40 domains, were also detected. Phylogenetic analysis of the *M*. *truncatula* U-box proteins grouped them into six subfamilies, and chromosomal mapping and synteny analyses indicated that tandem and segmental duplications may have contributed to the expansion and evolution of the U-box gene family in this species. Using RNA-seq data from *M*. *truncatula* seedlings subjected to three different abiotic stresses, we identified 33 stress-inducible plant U-box genes (*MtPUBs*). Specifically, 25 salinity-, 15 drought-, and 16 cold-regulated *MtPUBs* were detected. Among them, *MtPUB10*, *MtPUB17*, *MtPUB18*, *MtPUB35*, *MtPUB42*, and *MtPUB44* responded to all three stress conditions. Expression profiling by qRT-PCR was consistent with the RNA-seq data, and stress-related elements were identified in the promoter regions. The present findings strongly indicate that U-box proteins play critical roles in abiotic stress response in *M*. *truncatula*.

## Introduction

Ubiquitin-mediated proteolysis is required for most cellular processes, and the pathway is mediated by three sequential ubiquitination enzymes, E1, E2, and E3. E3 ubiquitin ligases are of particular importance as they confer substrate specificity that catalyzes the attachment of ubiquitin to protein targets [1,2]. The E3 ligases can be categorized into distinct families based on their protein domains (RING, HECT, or U-box domains) or mode of action [3,4]. The U-box E3 ligases, of which there are 64 members in *Arabidopsis*, were identified most recently and comprise the smallest E3 ligase family [5]. They have an approximately 70-amino-acid conserved U-box motif, which is present in U-box E3 ligases from yeast to humans [6]. A large expansion of the U-box gene family occurred in plants, which may be attributable to biological processes that are unique to the plant life cycle. It has been reported that plant U-box (PUB) proteins are largely involved in abiotic and biotic stress responses [7].

The *Arabidopsis* PUB protein AtCHIP plays an important role in temperature stress tolerance [8]. U-BOX17, another *Arabidopsis* PUB protein, and its tobacco homolog ACRE276 have been identified as positive regulators of cell death and defense [9], and subsequent studies yielded similar findings for the functions of these PUB proteins. AtPUB22 and AtPUB23 were found to have critical combinatory roles in response to drought stress [10], and they directly ubiquitinate RPN6, a 26S proteasome lid subunit, for subsequent degradation in *Arabidopsis* [7]. Similarly, AtPUB18 has a function linked to that of AtPUB19 in the negative regulation of ABA-mediated drought stress responses [11]. AtPUB13 acts as a node that connects flowering time regulation and salicylic acid (SA)-dependent defense signaling in *Arabidopsis* [12]. AtPUB30 acts in salt stress tolerance as a negative factor whose activity during germination is ABA independent [13]. The roles of PUBs in response to abiotic stresses have also been shown in other plants. For example, rice (*Oryza sativa*) *Spotted leaf11* (*Spl11*) encodes a U-box-containing E3 ligase and negatively regulates plant cell death and defense [14]. OsPUB15 helps reduce cellular oxidative stress during seedling establishment [15], and its ARM repeat domain is essential for its physical interaction with the kinase domain of PID2 (PID2K), an interaction observed both *in vitro* and *in vivo* [16]. *OsUPS*, another gene encoding a U-box-containing E3 ligase, responds to phosphate starvation in rice [17]. In hot pepper (*Capsicum annuum* L. cv. Pukang), CaPUB1 has been implicated in counteracting dehydration and high-salinity stress [18].

Efforts have been made to characterize these U-box genes in plant species as well as algae. Thus far, 30 full-length U-box genes have been identified in the *Chlamydomonas reinhardtii* genome sequence [19]. In *Arabidopsis* and rice, 64 and 77 U-box genes have been identified, respectively [20,21]. However, U-box genes have not been studied in the model legume *M*. *truncatula*. Here, we present a comprehensive analysis of the genes encoding U-box family proteins in *M*. *truncatula*.

## Materials and methods

### Identification of PUB proteins

Putative PUB proteins were identified in the *M*. *truncatula* genome database (http://www.medicagohapmap.org/tools/Blastform) using the BLAST program and the amino acid sequences of published U-box proteins as queries. The proteins identified by the BLAST program were used for domain searches from the Pfam (http://www.sanger.ac.uk/Software/Pfam/) and SMART (http://smart.embl-heidelberg.de/) databases with an E-value cut-off level of 1.0 or 10. These cut-off values were recommended for more reliable search results. Using the Pfam/SMART databases, the C-terminal domain of each PUB protein was analyzed with an E-value cut-off level of 1.0.

### Alignments, phylogenetic analysis, intron/exon organization, and localization of PUB genes on chromosomes

The U-box domain (PF00646) was obtained from the Pfam database, and HMMER 3.0 (http://hmmer.janelia.org/) was used for U-box motif identification in each PUB protein. Clustal X (version 2.0; http://www.clustal.org/) was used for the multiple sequence alignment of all predicted U-box protein motifs. A neighbor-joining (NJ) tree was constructed by MEGA (version 5.1; Tamura et al. 2011), using the p-distance method with gaps treated by pairwise deletion and a 1,000 bootstrap replicate. Intron/exon organization was determined using the *M*. *truncatula* genome database (http://www.medicagohapmap.org/home/view), and chromosomal maps were generated using the Genome Pixelizer Tcl/Tk script [www.atgc.org/GenomePixelizer (released 02/15/2002)]. Gene duplication was defined according to the following criteria: (1) The length of the sequence alignment covered ≥80% of the longest gene, and (2) the similarity of the aligned gene regions was ≥70% [22,23].

### Promoter element analysis

To investigate *cis* elements in the promoter sequences of the U-box protein-encoding genes, the 1,500 bp DNA sequences upstream of the transcriptional start site were obtained from NCBI (http://www.ncbi.nlm.nih.gov/). The PlantCARE database (http://bioinformatics.psb.ugent.be/webtools/plantcare/html/) was used to identify *cis* elements in the promoters and to collect data for the following: ABRE, ARE, AuxRR core, CGTCA motif, ERE, GARE motif, HSE, LTR, MBS, P Box, TC-rich repeat, TCA element, TGA element, and TGACG motif.

### Plant materials and stress treatments

*M*. *truncatula* seeds were soaked with distilled water and placed on a plastic net floating on 1/4-strength Hoagland nutrient solution (1.0 mM Ca^2+^, 1.5 mM K^+^, 0.5 mM Mg^2+^, 0.25 mM NH_4_^+^, 3.5 mM NO_3_^-^, 0.25 mM H_2_PO_4_^-^, 0.523 mM SO_4_^2-^, 22 μM Fe^2+^, 0.30 μM Cu^2+^, 0.8 μM Zn^2+^, 9 μM Mn^2+^, 46 μM BO_3_^3-^, 0.1 μM MoO_4_^2-^). After germination, seedlings were grown under the following conditions for 4 weeks: 22–24°C, 200 μmol m^−2^s^−1^ photosynthetic active radiation, and a photoperiod of 14/10 h (day/night).

Four weeks after germination, seedlings were subjected to various treatments. For drought treatment, the seedlings were transferred to dry Whatman 3MM paper in a sterile petri dish for 0, 2, 6, and 12 h. For cold treatment, the seedlings were transferred to 4°C for 0, 2, 6, and 12 h. For salt treatment, the seedlings were transferred to solutions containing 300 mM NaCl for 0, 2, 6, and 12 h. After treatment, the seedlings were harvested, immediately frozen in liquid nitrogen, and stored at -80°C for further analysis.

### Statistical analysis

Experiments in the study were independently performed in triplicate. Each result in this study is the mean of at least three replicated treatments and each treatment contained at least 10 plants. The significant differences between treatments were statistically assessed by standard deviation and one-way analysis of variance (ANOVA). The data between differently treated groups were compared statistically by ANOVA, followed by the least significant difference (LSD) test if the ANOVA result was significant at P<0.05.

### Library construction and sequencing

For RNA-seq analyses, RNA was extracted using Trizol. The 3’-tag digital gene expression libraries were prepared using the Illumina Gene Expression Sample Prep Kit based on the method described by Zhou *et al*. [24]. Deep sequencing were carried out using the Illumina HiSeq 3000 platform (Illumina, San Diego, CA, USA) following the manufacturer’s instructions by Genergy Biotechnology Co. Ltd. (Shanghai, China). The raw data comprised 100-bp paired-end sequences, and the cleaned reads were then mapped to Arabidopsis thaliana genome (TAIR10) using default settings of TOPHATv2.0.8. The duplicated reads were removed and alignments with MAPQ score > 20 were used for further analysis. RNA-seq alignments were processed using HTSeq-count, and differentially expressed genes were identified using DESeq with |log_2_ fold change| > 3.5.

## Results

### Identification and homology analysis of U-box proteins in *M*. *truncatula*

U-box domains (PF04564) were downloaded from the Pfam database and used as queries to identify U-box proteins in the *M*. *truncatula* genome database (http://www.medicagohapmap.org/tools/Blastform) using the BLAST program (HMMER 3.0, http://hmmer.janelia.org/). The identified proteins were used for a domain search of the Pfam (http://www.sanger.ac.uk/Software/Pfam/) and SMART (http://smart.embl-heidelberg.de/) databases with an E-value cut-off level of 1.0 or 10, which was recommended for more reliable search results. Using the Pfam/SMART databases, the C-terminal domains of each U-box protein with an E-value cut-off level of 1.0 were analyzed. We found 64 proteins containing at least one U-box motif in *M*. *truncatula* as annotated by the SMART/Pfam databases, and these proteins were designated as U-box proteins (MtPUB) (Table 1 and S1 Table). The isoelectric point (pI) bias of most of these U-box proteins was neutral. Only MtPUB10 and MtPUB11 had a pI greater than 10, and only MtPUB62 had a pI less than 5 (Table 1). Some of the genes encoding these U-box proteins had numerous introns; for example, *MtPUB9*, *MtPUB39*, *MtPUB47*, and *MtPUB64* all had more than 10 introns (Table 1).

**Table 1:** Distribution of *MtPUB* genes in the *Medicago truncatula* genome.

S.No	Gene_ID	Accession number	Other domain	Predicted protein (aa)	Mol wt (kDa)	pI	Chromosome	No. of introns
1	*MtPUB1*	Medtr1g017770.1	Unknown	434	48.39	6.88	1	0
2	*MtPUB2*	Medtr1g056840.1	Unknown	411	46.09	8.47	1	1
3	*MtPUB3*	Medtr1g056870.1	Unknown	437	48.80	6.95	1	0
4	*MtPUB4*	Medtr1g056880.1	Unknown	437	48.90	7.91	1	0
5	*MtPUB5*	Medtr1g056910.1	Unknown	406	46.25	8.54	1	0
6	*MtPUB6*	Medtr1g069845.1	ARM	608	66.67	6.52	1	4
7	*MtPUB7*	Medtr1g076400.1	Unknown	1013	112.48	5.09	1	3
8	*MtPUB8*	Medtr1g079450.1	Unknown	446	49.65	8.05	1	1
9	*MtPUB9*	Medtr1g090320.1	WD40	1488	166.85	5.96	1	16
10	*MtPUB10*	Medtr1g093965.1	Unknown	200	21.84	10.05	1	3
11	*MtPUB11*	Medtr1g093995.1	Unknown	200	21.86	10.05	1	3
12	*MtPUB12*	Medtr1g094025.1	Unknown	296	33.24	8.14	1	3
13	*MtPUB13*	Medtr1g094215.1	ARM	447	48.10	6.13	1	3
14	*MtPUB14*	Medtr1g100820.1	Kinase	715	80.80	5.44	1	7
15	*MtPUB15*	Medtr2g007630.1	Unknown	259	28.73	9.58	2	3
16	*MtPUB16*	Medtr2g011140.1	Unknown	383	42.16	6.77	2	0
17	*MtPUB17*	Medtr2g018010.1	ARM	720	78.67	6.58	2	0
18	*MtPUB18*	Medtr2g087350.1	Unknown	403	45.33	8.61	2	0
19	*MtPUB19*	Medtr2g096850.1	Kinase	810	91.60	7.01	2	6
20	*MtPUB20*	Medtr3g008270.1	Kinase	797	88.60	6.48	3	9
21	*MtPUB21*	Medtr3g008280.1	Kinase	809	90.02	7.21	3	9
22	*MtPUB22*	Medtr3g065080.1	Unknown	439	49.13	8.08	3	0
23	*MtPUB23*	Medtr3g078160.1	Unknown	681	75.94	8.29	3	0
24	*MtPUB24*	Medtr3g078340.1	ARM	529	57.91	7.03	3	0
25	*MtPUB25*	Medtr3g085610.1	KAP	766	84.92	6.10	3	5
26	*MtPUB26*	Medtr3g095730.1	Unknown	419	46.77	8.92	3	0
27	*MtPUB27*	Medtr3g096370.1	Unknown	404	45.02	6.35	3	0
28	*MtPUB28*	Medtr3g115670.1	HEAT	814	89.47	5.06	3	3
29	*MtPUB29*	Medtr3g466220.1	ARM	836	90.68	5.45	3	3
30	*MtPUB30*	Medtr4g028960.1	ARM	701	76.42	6.83	4	0
31	*MtPUB31*	Medtr4g051515.1	Unknown	413	47.12	9.26	4	0
32	*MtPUB32*	Medtr4g063800.1	ARM	662	72.09	5.14	4	3
33	*MtPUB33*	Medtr4g085720.1	Unknown	410	45.35	7.81	4	0
34	*MtPUB34*	Medtr4g091880.1	Unknown	375	40.59	8.36	4	0
35	*MtPUB35*	Medtr4g107010.1	ARM	747	83.52	8.04	4	1
36	*MtPUB36*	Medtr4g125930.1	Kinase	764	85.49	6.00	4	8
37	*MtPUB37*	Medtr4g485520.1	ARM	652	70.78	7.01	4	3
38	*MtPUB38*	Medtr5g015210.1	Unknown	451	49.54	6.50	5	0
39	*MtPUB39*	Medtr5g015500.1	Pro isomerase	552	59.75	7.65	5	10
40	*MtPUB40*	Medtr5g020570.1	KAP	782	88.26	6.44	5	5
41	*MtPUB41*	Medtr5g032010.1	Kinase	808	92.93	7.98	5	8
42	*MtPUB42*	Medtr5g034440.1	ARM	689	76.82	7.19	5	0
43	*MtPUB43*	Medtr5g048050.1	Unknown	438	50.05	6.88	5	0
44	*MtPUB44*	Medtr5g077510.1	Unknown	442	49.45	8.53	5	0
45	*MtPUB45*	Medtr5g083030.1	ARM	694	76.93	6.78	5	0
46	*MtPUB46*	Medtr6g008170.1	KAP	554	61.48	8.22	6	0
47	*MtPUB47*	Medtr6g013690.1	Ufd2p	1047	11.80	5.47	6	15
48	*MtPUB48*	Medtr6g071340.1	Unknown	418	47.61	5.56	6	0
49	*MtPUB49*	Medtr7g005940.1	Unknown	1073	12.18	7.21	7	8
50	*MtPUB50*	Medtr7g053260.1	Unknown	459	51.53	8.39	7	1
51	*MtPUB51*	Medtr7g059405.1	ARM	634	69.82	6.27	7	4
52	*MtPUB52*	Medtr7g077780.1	Kinase	896	100.38	6.02	7	8
53	*MtPUB53*	Medtr7g078330.1	ARM	646	72.78	5.05	7	3
54	*MtPUB54*	Medtr7g097020.1	ARM	767	84.39	7.44	7	5
55	*MtPUB55*	Medtr7g106340.1	Unknown	421	46.96	8.71	7	0
56	*MtPUB56*	Medtr7g116600.1	Unknown	460	51.32	8.35	7	1
57	*MtPUB57*	Medtr7g117890.1	ARM	1001	111.25	5.30	7	4
58	*MtPUB58*	Medtr8g011720.1	TPR	277	31.95	6.38	8	7
59	*MtPUB59*	Medtr8g027140.1	Unknown	1006	112.03	5.62	8	4
60	*MtPUB60*	Medtr8g068530.1	Kinase	769	88.97	5.81	8	7
61	*MtPUB61*	Medtr8g077205.1	KAP	760	85.19	6.66	8	4
62	*MtPUB62*	Medtr8g080280.1	Unknown	767	85.42	4.87	8	5
63	*MtPUB63*	Medtr8g092870.1	Unknown	418	46.35	7.48	8	0
64	*MtPUB64*	Medtr8g103227.1	WD40	1335	148.78	5.64	8	14

### Analysis of the functional domains of the *M*. *truncatula* U-box proteins

**Fig 1:**
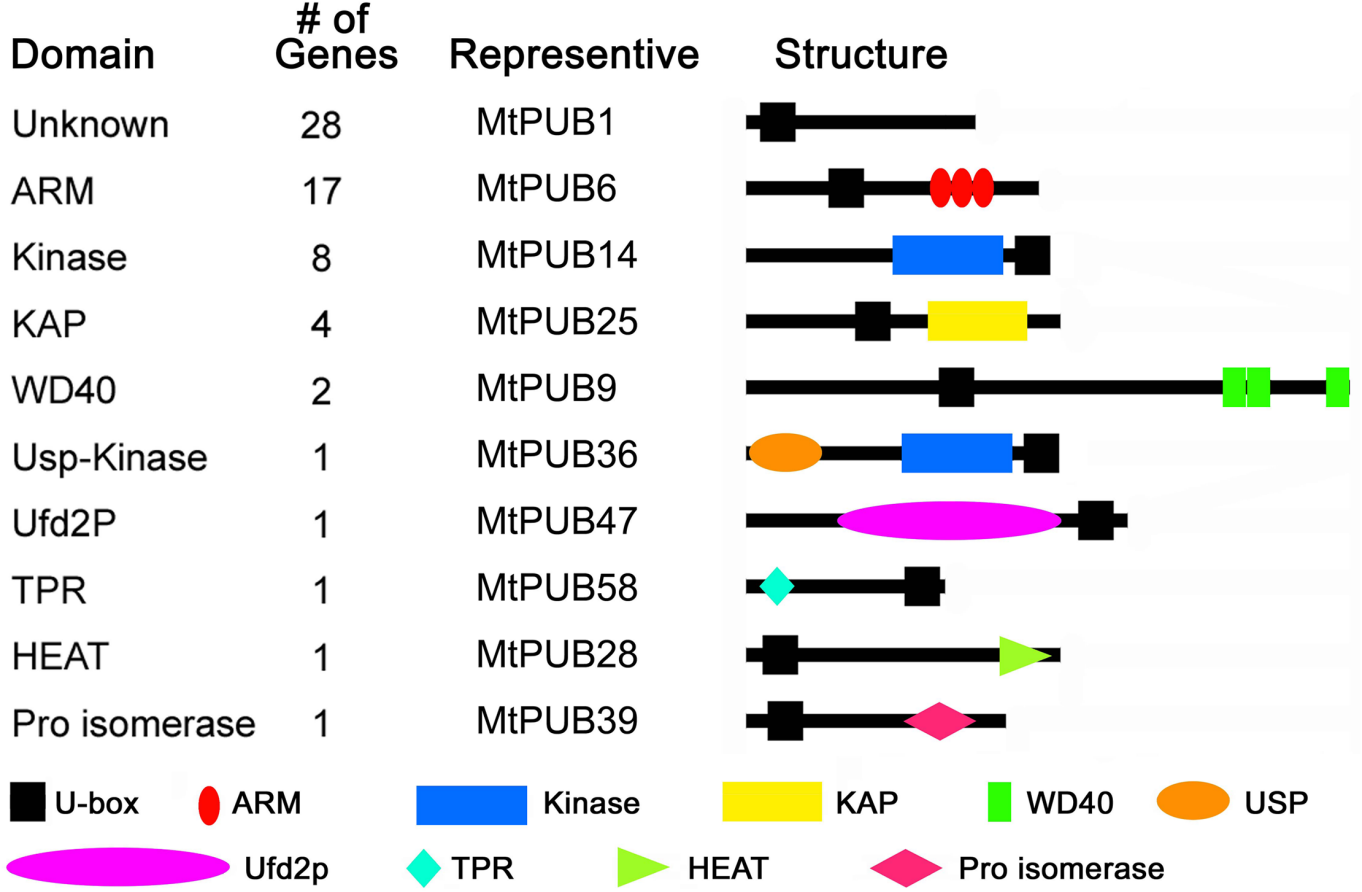
Number and domain structure of U-box proteins in *Medicago truncatula*. Shown on the left are the types of functional domains and the number of U-box proteins predicted to have those domains. The domain names are taken from the Pfam or SMART database. Domain abbreviations: Unknown, U-box proteins that have no obvious N- or C-terminal interaction domain or have rare or functionally uncertain domains; ARM, Armadillo/beta-catenin-like repeat; Kinase, protein tyrosine kinase; KAP, kinesin-associated protein; WD40, WD40 domain, G-beta repeat; USP-Kinase; Ufd2p, ubiquitin elongating factor core; TPR2, TPR repeats; HEAT, HEAT repeats; Pro isomerase, cyclophilin-type peptidyl-prolyl cis-trans isomerase/CLD.

U-box proteins often contain several other functional domains at their N- or C-terminal regions. The SMART and Pfam database searches revealed that the U-box proteins contained several known or unknown conserved domains, which presumably participate in substrate recognition, and we designated these domains as functional domains (Fig 1). The types of functional domains in the U-box proteins are listed in Table 1. The 36 U-box proteins with one or more known functional domains were as follows, with the number in parentheses indicating the number of proteins: ARM(17), Armadillo/beta-catenin-like repeat; Kinase(8), protein tyrosine kinase; KAP(4), kinesin-associated protein; WD40(2), WD40 domain, G-beta repeat; USP-Kinase(1); Ufd2p(1), ubiquitin elongating factor core; TPR (1), TPR repeats; HEAT(1), HEAT repeats; and Pro isomerase(1), cyclophilin-type peptidyl-prolyl cis-trans isomerase/CLD. Some U-box proteins had no other obvious interaction domains or had a few rare or functionally uncertain domains; all of these were classified together as ‘Unknown’ (Fig 1).

Aside from the U-box motif, the ARM (Armadillo/beta-catenin-like repeat) domain, an approximately 40-amino-acid tandemly repeated sequence motif, was the most highly represented functional domain among the identified MtPUB proteins. In beta-catenin, these tandem repeats form a super-helix of helices that presumably mediates ligand interaction (Fig 1). U-box-ARM proteins have been reported in *Arabidopsis*. For example, *AtPUB18* and *AtPUB19* have related functions in negatively regulating ABA-mediated drought stress response [11]. The homologs of *AtPUB18* and *AtPUB19* in *M*. *truncatula* are *MtPUB35* and *MtPUB42* (S1 Fig). In *Medicago truncatula*, *MtPUB35* and *MtPUB42* have high sequence similarities with *AtPUB18* and *AtPUB19* (S1 Fig). *MtPUB32* also has high sequence similarity with *AtPUB13*, which encodes a U-box-ARM protein that links the flowering time and SA-dependent defense signaling pathways in Arabidopsis [12] (S1 Fig and S1 Table). U-box-ARM protein *AtPUB30* acts in salt stress tolerance as a negative factor independent of ABA during seed germination [13], and it is homologous to *MtPUB38* (S1 Fig). In rice, the U-box-ARM E3 ligase SPL11 negatively regulates plant cell death and defense[14]. OsPUB15, another U-box-ARM protein in rice, helps reduce cellular oxidative stress during seedling establishment [15]. *OsPUB15* is homologous to *MtPUB29* in *M*. *truncatula* (S1 Fig).

Eight MtPUB proteins were found to have a kinase domain, indicating their potential involvement in signal transduction cascades via phosphorylation. The KAP (kinesin-associated protein) domain, found in four MtPUB proteins, is associated with motor function, consistent with the role of kinesins as intracellular multimeric transport motor proteins that move cellular cargo on microtubule tracks.

Two MtPUB proteins had WD40 domains. WD40 domain-containing proteins are made up of highly conserved repeating units approximately 40 amino acids long and usually ending with Trp-Asp (WD) [25]. They are found in all eukaryotes but not in prokaryotes, and they regulate numerous cellular functions, such as cell division, cell-fate determination, gene transcription, transmembrane signaling, mRNA modification, and vesicle fusion. The USP, Ufd2p, TPR, HEAT, and Pro isomerase domains were each present in only one MtPUB protein (Fig 1). WD40 and TPR domains are known to be involved in protein interactions [26,27]. Rice and *Arabidopsis* U-box proteins containing WD40 repeats are homologous to animal and human Prp19p proteins and are involved in preRNA splicing and other biological processes [7,28,29]. AtCHIP, the only TPR domain-containing U-box protein in *Arabidopsis*, is homologous to the mammalian CHIP (carboxyl terminus of Hsc70-interacting protein) and participates in abiotic stress response and the regulation of chloroplast protein turnover [30,31]. In humans and animals, CHIP interacts with molecular chaperones, such as Hsp70 and Hsp90, and acts as a partner in the cell to ensure protein stability. CHIP is involved in cell stress protection and several neurodegenerative diseases [32,33]. The homolog of *AtCHIP* in *M*. *truncatula* is *MtPUB58* (S1 Fig).

### Phylogenetic and evolutionary analysis of U-box proteins in *M*. *truncatula*

**Fig 2:**
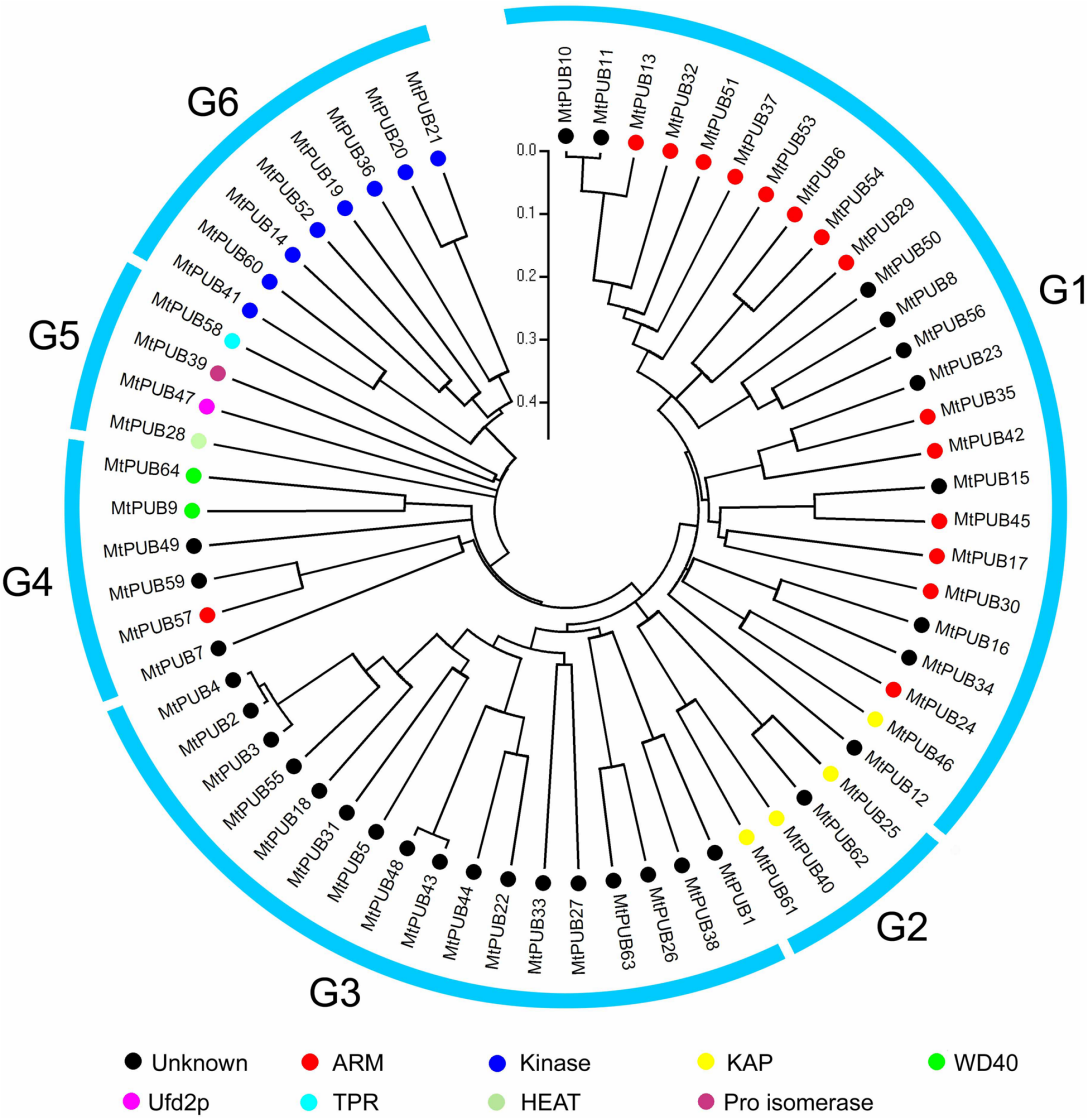
Phylogenetic tree of the U-box protein family from *Medicago truncatula*. The 70-amino-acid U-box motifs from the 64 putative U-box proteins were aligned by CLUSTAL X 2.0, and the unrooted NJ phylogenetic tree was constructed by MEGA 5.1, using the p-distance method and a bootstrap value of 1,000. The six groups identified from the phylogenetic analysis are marked on the outside. The bar represents the branch length equivalent to 0.05 amino acid changes per residue. Table 1 provides additional information for the corresponding genes.

For the phylogenetic analysis of the identified U-box proteins, we used HMMER 3.0 software (http://hmmer.janelia.org/) to analyze the motif sequences of each U-box protein. All of the U-box proteins were found to contain only one U-box motif. Using the U-box motif sequence for the alignment, an unrooted phylogenetic tree of the entire dataset was created (Fig 2). The phylogenetic tree divided the 64 MtPUB proteins into six subfamilies according to the distribution of various branches, the length of each branch, and the phylogenetic relationship between MtPUB proteins.

The phylogenetic tree was color-coded according to the different functional domains (Fig 2). Most of the kinase domain-containing MtPUB proteins were in the G6 family. The ARM-containing MtPUB proteins generally localized in clades within the G1 family. This correlation further supports the phylogenetic relationships in the U-box tree and suggests a co-evolution of the U-box motif with other domains.

### Locations of the U-box protein-encoding genes on *M*. *truncatula* chromosomes

**Fig 3:**
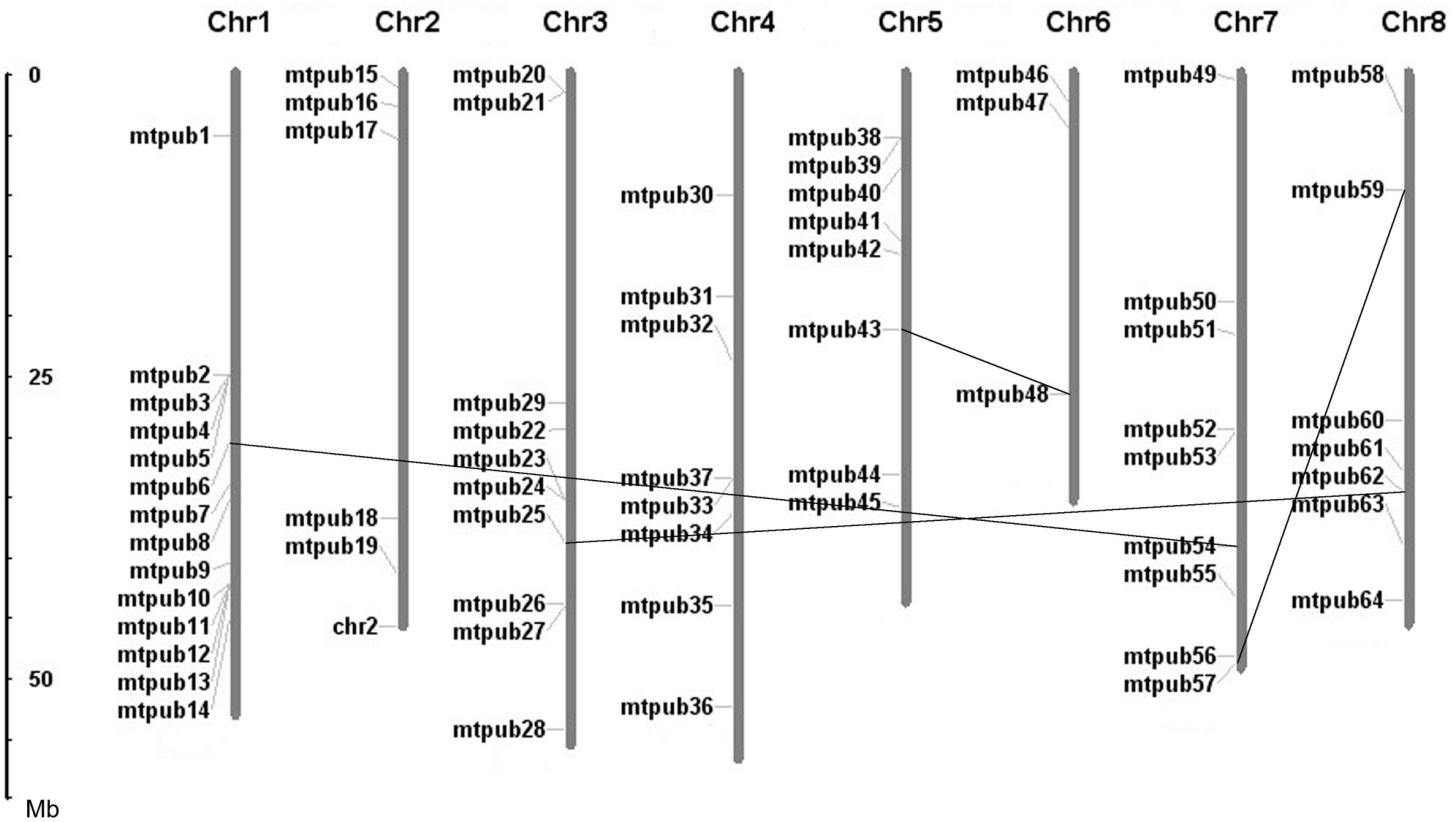
Locations and duplications of *Medicago truncatula* U-box genes on chromosomes 1–8. Genes lying on duplicated segments of genome have been joined by lines. The scale represents megabases (Mb). The chromosome numbers are indicated at the top of each bar.

The U-box protein-encoding genes were distributed randomly on all eight *M*. *truncatula* chromosomes. To determine whether the gene family in *M*. *truncatula* evolved through duplication events, we obtained the chromosomal locations of the U-box protein-encoding genes from the *M*. *truncatula* genomic database and mapped the loci on the chromosomes (Fig 3). With 14 U-box genes, chromosome 1 had the largest number, whereas chromosome 6 had only three U-box genes. Some U-box genes were arranged in tandem repeats either in the same or inverse orientation, representing local gene duplications. As shown in Fig 3, there were four segmental duplication events between chromosomes, suggesting that tandem duplications of chromosomal regions played a major role in the expansion of this gene family.

### Expression analysis of U-box protein-encoding genes in various tissues

**Fig 4:**
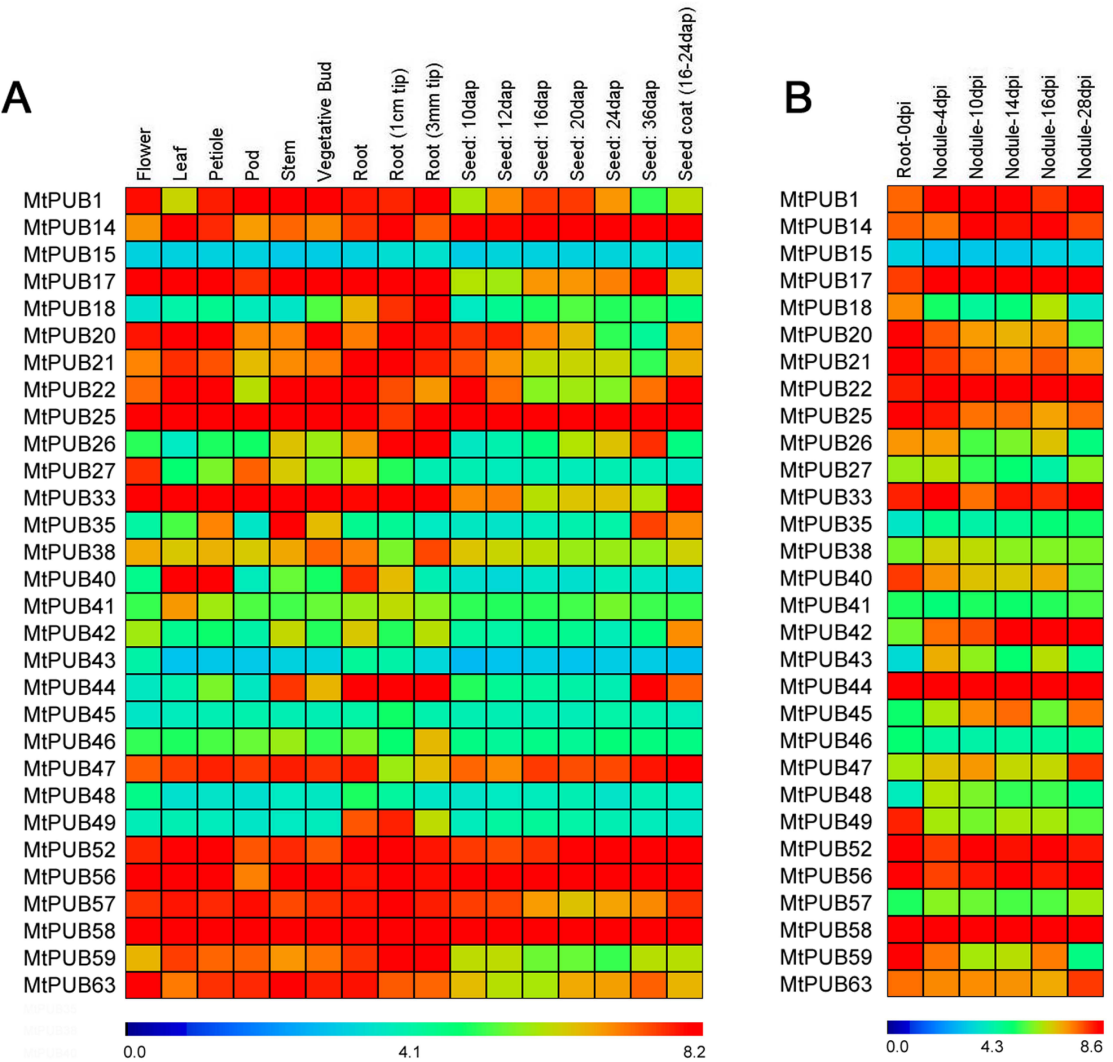
**Expression profiles of *Medicago truncatula* U-box protein-encoding genes during panicle development (A) and root development (B).** The average log signal values of U-box protein-encoding genes in various tissues/organs and developmental stages (mentioned at the top of each lane) are presented. The data comes from this site (http://mtgea.noble.org/v2/annotation_search_form.php#gid). The color scale (representing log signal values) is shown at the bottom. dap: days after pollination.

Using an existing database (http://mtgea.noble.org/v2/), we were able to survey the expression of many *MtPUB* genes in different tissues. A few *MtPUBs* were expressed only in certain tissues. For example, *MtPUB18* and *MtPUB49* were mainly expressed in roots; *MtPUB40* expression was largely restricted to leaves and roots; *MtPUB27* was expressed in flowers and pods; and *MtPUB44* was expressed in roots and mature seeds (Fig 4A). Because legume root nodules plays an important role in symbiotic nitrogen fixation, we also identified *MtPUBs* that were differentially expressed in the nodule. Strong expression of *MtPUB42* and *MtPUB47* could be seen in root nodules, while the expression of *MtPUB18*, *MtPUB40*, and *MtPUB49* in root nodules was low (Fig 4B).

### Identification of stress-responsive *MtPUBs*

**Fig 5:**
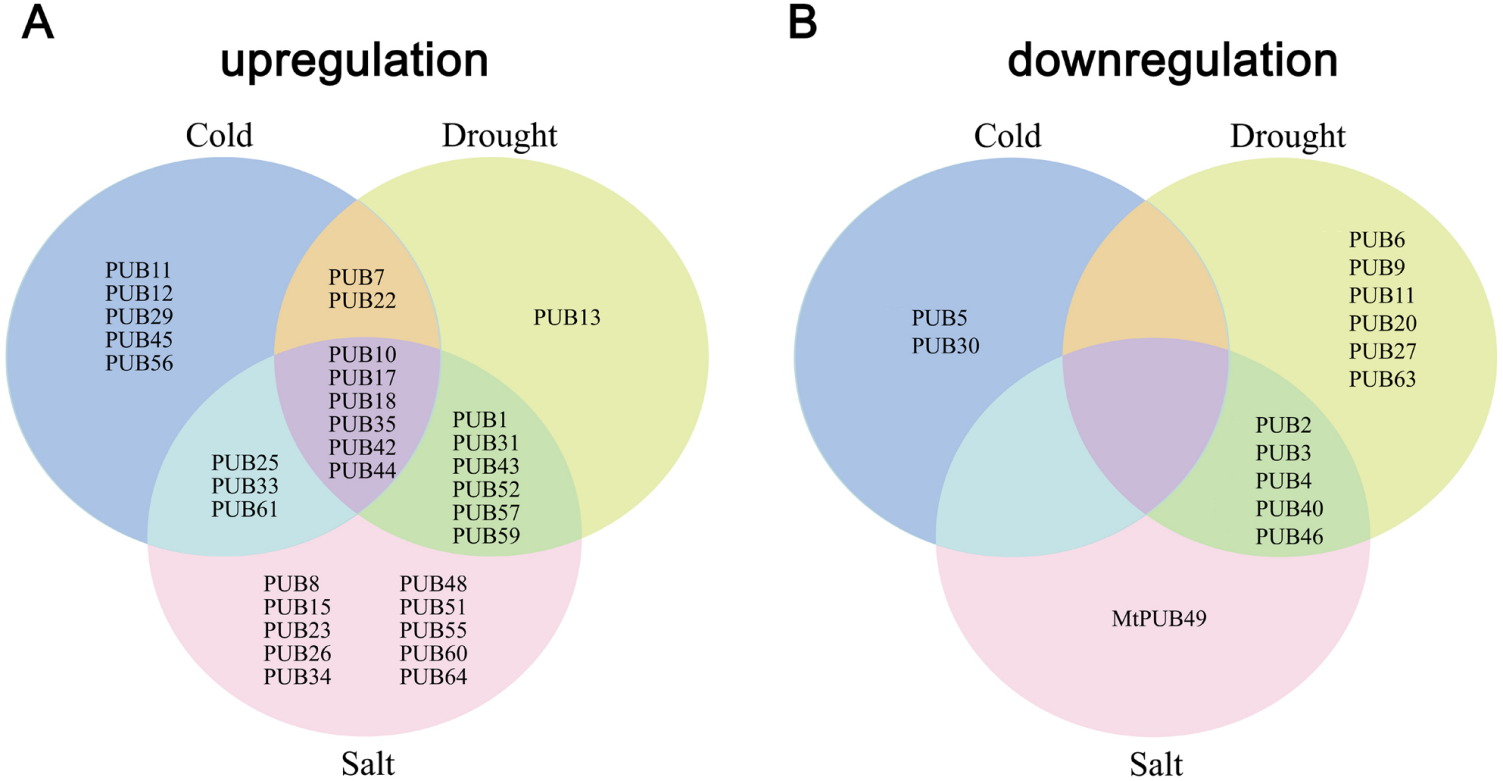
Venn diagram showing common and unique differential *MtPUB* gene expression under three treatment conditions. Among them, 25 high-salinity-, 15 drought-, and 16 cold- up regulated U-box genes were detected and 6 U-box genes were observed to respond remarkably to all three stresses. in contrast, 6 high-salinity-, 11 drought-, and 2 cold- down regulated U-box genes were detected.

To study the expression of the U-box family genes under abiotic stress, 4-week-old *M*. *truncatula* seedlings were collected and treated with drought, salt, or cold stress for 0, 2, 6, and 12 h. Total RNA was extracted, and libraries were constructed for RNA-seq. In general, under drought, salt, and cold stress, there were more up-regulated genes than down-regulated genes, and the difference was most obvious at 2 h (S2 Fig). Salt stress had the strongest correlation with drought stress, and the R value was more than 0.95 at 2, 6, and 12 h (S3 Fig). The analysis showed that some of the 64 U-box family genes could be induced by salt, drought, or cold stress, but a few genes were down-regulated (Fig 5, Tables 2–4). After drought treatment, *MtPUB1*, *MtPUB7*, *MtPUB10*, *MtPUB13*, *MtPUB17*, *MtPUB18*, *MtPUB22*, *MtPUB31*, *MtPUB35*, *MtPUB42*, *MtPUB43*, *MtPUB44*, *MtPUB52*, *MtPUB57*, and *MtPUB59* were up-regulated (Table 2). (A gene was considered up-regulated if its expression was increased at 2, 6, and 12 h and if the log_2_ fold change > 1 for at least one of these time points.) Using the same criteria, we found that *MtPUB1*, *MtPUB8*, *MtPUB10*, *MtPUB15*, *MtPUB17*, *MtPUB18*, *MtPUB23*, *MtPUB25*, *MtPUB26*, *MtPUB31*, *MtPUB33*, *MtPUB34*, *MtPUB35*, *MtPUB42*, *MtPUB43*, *MtPUB44*, *MtPUB48*, *MtPUB51*, *MtPUB52*, *MtPUB55*, *MtPUB57*, *MtPUB59*, *MtPUB60*, *MtPUB61*, and *MtPUB64* were up-regulated under salt stress (Table 3). After cold treatment, *MtPUB7*, *MtPUB10*, *MtPUB11*, *MtPUB12*, *MtPUB17*, *MtPUB18*, *MtPUB22*, *MtPUB25*, *MtPUB29*, *MtPUB33*, *MtPUB35*, *MtPUB42*, *MtPUB44*, *MtPUB45*, *MtPUB56*, and *MtPUB61* were up-regulated (Table 4).

**Table 2:** Read abundance of *MtPUB* genes in the drought-0, drought-2, drought-6, and drought-12 libraries.

Gene_ID	Drought-0	Drought-2	Drought-6	Drought-12	log_2_ (Drought-2/ Drought-0)	log_2_ (Drought-6/ Drought-0)	log_2_ (Drought-12/ Drought-0)
*MtPUB1*	79	146	178	111	0.89	1.17*	0.49
*MtPUB2*	59	27	13	4	-1.11*	-2.17*	-3.91*
*MtPUB3*	69	41	14	13	-0.76	-2.29*	-2.45*
*MtPUB4*	158	76	41	14	-1.05*	-1.97*	-3.49*
*MtPUB5*	62	176	53	55	1.51*	-0.23	-0.16
*MtPUB6*	299	200	91	131	-0.58	-1.71*	-1.19*
*MtPUB7*	348	841	784	717	1.27*	1.17*	1.04*
*MtPUB8*	3	6	1	4	1.07*	-1.53*	0.44
*MtPUB9*	941	633	624	343	-0.57	-0.59	-1.46*
*MtPUB10*	10	11	23	35	0.09	1.13*	1.72*
*MtPUB11*	4	4	1	2	-0.12	-1.94*	-0.64
*MtPUB12*	5	9	9	7	0.85	0.86	0.51
*MtPUB13*	463	911	1106	901	0.98	1.26*	0.96
*MtPUB14*	393	446	539	442	0.18	0.46	0.17
*MtPUB15*	2	3	2	2	0.47	0.11	0.34
*MtPUB16*	1	1	1	1	0	0	0
*MtPUB17*	520	730	1218	905	0.49	1.23*	0.80
*MtPUB18*	51	155	122	67	1.59*	1.25*	0.38
*MtPUB19*	526	661	798	914	0.33	0.60	0.80
*MtPUB20*	449	393	191	232	-0.19	-1.23*	-0.95
*MtPUB21*	448	478	281	325	0.09	-0.67	-0.46
*MtPUB22*	119	336	237	176	1.50*	1.00*	0.57
*MtPUB23*	174	96	452	560	-0.85	1.38*	1.69*
*MtPUB24*	444	449	484	468	0.02	0.13	0.08
*MtPUB25*	274	304	286	354	0.15	0.06	0.37
*MtPUB26*	219	340	233	222	0.63	0.09	0.02
*MtPUB27*	143	143	69	46	0.00	-1.05*	-1.63*
*MtPUB28*	1009	1266	1309	1224	0.33	0.38	0.28
*MtPUB29*	768	883	1182	953	0.20	0.62	0.31
*MtPUB30*	361	308	313	283	-0.23	-0.21	-0.35
*MtPUB31*	26	362	60	36	3.82*	1.23*	0.49
*MtPUB32*	1348	1690	1839	1330	0.33	0.45	-0.02
*MtPUB33*	159	328	150	165	1.04*	-0.08	0.05
*MtPUB34*	238	209	254	251	-0.19	0.09	0.07
*MtPUB35*	47	1924	3780	3587	5.34*	6.32*	6.24*
*MtPUB36*	461	771	700	702	0.74	0.60	0.61
*MtPUB37*	561	394	355	321	-0.51	-0.66	-0.81
*MtPUB38*	169	233	97	81	0.47	-0.81	-1.05*
*MtPUB39*	415	462	571	540	0.15	0.46	0.38
*MtPUB40*	688	662	176	184	-0.06	-1.97*	-1.91*
*MtPUB41*	405	361	681	712	-0.16	0.75	0.81
*MtPUB42*	36	439	1544	912	3.61*	5.42*	4.66*
*MtPUB43*	10	70	10	27	2.87*	0.04	1.52*
*MtPUB44*	226	1168	693	341	2.37*	1.62*	0.60
*MtPUB45*	6	21	9	5	1.90*	0.60	-0.09
*MtPUB46*	208	95	109	96	-1.13*	-0.94	-1.11*
*MtPUB47*	1422	1781	2647	2632	0.32	0.90	0.89
*MtPUB48*	32	99	32	45	1.62*	-0.02	0.47
*MtPUB49*	279	153	160	143	-0.87	-0.80	-0.97
*MtPUB50*	178	126	136	154	-0.50	-0.39	-0.21
*MtPUB51*	119	110	113	127	-0.10	-0.07	0.09
*MtPUB52*	806	1059	1823	2081	0.39	1.18*	1.37*
*MtPUB53*	1	1	1	1	0	0	0
*MtPUB54*	222	156	237	157	-0.51	0.10	-0.50
*MtPUB55*	96	436	89	29	2.19*	-0.11	-1.74*
*MtPUB56*	261	284	156	220	0.12	-0.74	-0.24
*MtPUB57*	832	1663	1887	1736	1.00*	1.18*	1.06*
*MtPUB58*	230	300	244	241	0.38	0.09	0.07
*MtPUB59*	328	584	609	728	0.83	0.89	1.15*
*MtPUB60*	1	1	6	1	0	2.70*	0
*MtPUB61*	262	456	431	470	0.80	0.72	0.84
*MtPUB62*	1125	1514	1961	1846	0.43	0.80	0.71
*MtPUB63*	149	135	76	67	-0.14	-0.98	-1.16*
*MtPUB64*	2	10	16	1	2.41*	3.07*	-0.96

Values indicate number of reads.

* indicates a significant difference in expression compared to the 0 h time point (*P* < 0.01 and |log_2_N| ≥ 1). Drought-0, Drought-2, Drought-6, and Drought-12 indicate 0, 2, 6, and 12 h drought treatment, respectively.

**Table 3:** Read abundance of *MtPUB* genes in the salt-0, salt-2, salt-6, and salt-12 libraries.

Gene_ID	Salt-0	Salt-2	Salt-6	Salt-12	log_2_ (Salt-2/Salt-0)	log_2_ (Salt-6/Salt-0)	log_2_ (Salt-12/Salt-0)
*MtPUB1*	109	142	158	223	0.38	0.54	1.03*
*MtPUB2*	44	18	13	24	-1.31*	-1.81*	-0.91
*MtPUB3*	44	42	13	13	-0.09	-1.81*	-1.78*
*MtPUB4*	159	81	54	42	-0.97	-1.56*	-1.91*
*MtPUB5*	61	43	70	147	-0.52	0.20	1.27*
*MtPUB6*	249	183	242	179	-0.45	-0.04	-0.48
*MtPUB7*	375	518	578	621	0.46	0.62	0.73
*MtPUB8*	1	3	1	10	1.73*	0	3.37*
*MtPUB9*	975	654	837	944	-0.58	-0.22	-0.05
*MtPUB10*	13	13	37	25	0.01	1.47*	0.91
*MtPUB11*	1	3	1	1	1.35*	0	0
*MtPUB12*	1	2	2	1	0.82	0.83	0
*MtPUB13*	484	809	775	927	0.74	0.68	0.94
*MtPUB14*	375	509	615	377	0.44	0.71	0.01
*MtPUB15*	1	9	7	4	3.25*	2.85*	1.87*
*MtPUB16*	1	1	3	1	0	1.74*	0
*MtPUB17*	527	752	854	1116	0.51	0.70	1.08*
*MtPUB18*	34	117	103	171	1.76*	1.58*	2.32*
*MtPUB19*	587	525	601	526	-0.16	0.04	-0.16
*MtPUB20*	534	351	428	483	-0.61	-0.32	-0.15
*MtPUB21*	464	383	408	667	-0.28	-0.18	0.52
*MtPUB22*	131	242	223	240	0.88	0.76	0.87
*MtPUB23*	191	217	348	428	0.18	0.86	1.16*
*MtPUB24*	393	458	450	585	0.22	0.19	0.57
*MtPUB25*	220	411	397	526	0.90	0.85	1.26*
*MtPUB26*	237	281	395	797	0.24	0.74	1.75*
*MtPUB27*	110	63	104	58	-0.80	-0.08	-0.92
*MtPUB28*	929	1282	1146	1239	0.47	0.30	0.42
*MtPUB29*	745	963	1003	1122	0.37	0.43	0.59
*MtPUB30*	419	340	396	989	-0.30	-0.08	1.24*
*MtPUB31*	28	92	108	367	1.73*	1.97*	3.73*
*MtPUB32*	1387	1959	2172	2297	0.50	0.65	0.73
*MtPUB33*	123	264	169	302	1.10*	0.45	1.29*
*MtPUB34*	177	249	254	388	0.49	0.52	1.13*
*MtPUB35*	80	1755	1351	1129	4.45*	4.08*	3.82*
*MtPUB36*	513	703	683	589	0.45	0.41	0.20
*MtPUB37*	483	491	490	550	0.02	0.02	0.19
*MtPUB38*	158	106	127	151	-0.58	-0.32	-0.06
*MtPUB39*	458	508	509	674	0.15	0.16	0.56
*MtPUB40*	807	220	240	153	-1.88*	-1.75*	-2.40*
*MtPUB41*	421	314	520	514	-0.42	0.31	0.29
*MtPUB42*	25	1145	1127	1003	5.49*	5.47*	5.30*
*MtPUB43*	13	32	34	72	1.27*	1.34*	2.43*
*MtPUB44*	248	528	507	1083	1.09*	1.03*	2.12*
*MtPUB45*	5	17	4	1	1.66*	-0.41	-2.45*
*MtPUB46*	274	104	142	119	-1.40*	-0.94	-1.20*
*MtPUB47*	1416	1755	2165	2306	0.31	0.61	0.70
*MtPUB48*	47	86	83	131	0.88	0.84	1.49*
*MtPUB49*	272	176	187	94	-0.63	-0.54	-1.53*
*MtPUB50*	207	208	240	137	0.01	0.21	-0.60
*MtPUB51*	110	127	168	251	0.20	0.61	1.19*
*MtPUB52*	908	1296	1477	1886	0.51	0.70	1.05*
*MtPUB53*	1	1	1	1	0	0	0
*MtPUB54*	209	191	351	307	-0.13	0.75	0.55
*MtPUB55*	83	289	93	259	1.79*	0.15	1.64*
*MtPUB56*	295	268	190	218	-0.14	-0.64	-0.44
*MtPUB57*	797	1701	1589	2026	1.09*	1.00*	1.35*
*MtPUB58*	236	298	291	302	0.34	0.30	0.35
*MtPUB59*	286	585	647	694	1.03*	1.18*	1.28*
*MtPUB60*	1	9	5	2	3.12*	2.29*	1.22*
*MtPUB61*	204	319	465	638	0.65	1.19*	1.65*
*MtPUB62*	1122	1812	1784	1790	0.69	0.67	0.67
*MtPUB63*	134	84	90	98	-0.68	-0.57	-0.44
*MtPUB64*	1	10	12	18	3.36*	3.57*	4.19*

Values indicate number of reads.

* indicates a significant difference in expression compared to the 0 h time point (*P* < 0.01 and |log_2_N| ≥ 1). Salt-0, Salt-2, Salt-6, and Salt-12 indicate 0, 2, 6, and 12 h salt treatment, respectively.

**Table 4:** Read abundance of *MtPUB* genes in the cold-0, cold-2, cold-6, and cold-12 libraries.

Gene_ID	Cold-0	Cold-2	Cold-6	Cold-12	log_2_ (Cold-2/Cold-0)	log_2_ (Cold-6/Cold-0)	log_2_ (Cold-12/Cold-0)
*MtPUB1*	83	154	43	50	0.90	-0.94	-0.72
*MtPUB2*	57	146	63	30	1.37*	0.14	-0.90
*MtPUB3*	68	149	71	54	1.13*	0.05	-0.33
*MtPUB4*	162	380	205	121	1.23*	0.34	-0.42
*MtPUB5*	60	48	28	21	-0.31	-1.11*	-1.54*
*MtPUB6*	283	290	400	446	0.04	0.50	0.66
*MtPUB7*	340	1346	1024	771	1.99*	1.59*	1.18*
*MtPUB8*	1	1	2	1		0.74	
*MtPUB9*	969	743	1315	1567	-0.38	0.44	0.69
*MtPUB10*	7	17	20	15	1.26*	1.50*	1.06*
*MtPUB11*	3	13	12	9	2.13*	2.01*	1.62*
*MtPUB12*	4	5	11	8	0.25	1.43*	0.96
*MtPUB13*	459	558	555	627	0.28	0.27	0.45
*MtPUB14*	370	401	396	446	0.12	0.10	0.27
*MtPUB15*	1	1	2	1	0	0.74	0
*MtPUB16*	1	1	1	1	0	0	0
*MtPUB17*	505	1059	686	750	1.07*	0.44	0.57
*MtPUB18*	56	76	271	152	0.45	2.28*	1.45*
*MtPUB19*	509	570	867	719	0.16	0.77	0.50
*MtPUB20*	448	487	483	508	0.12	0.11	0.18
*MtPUB21*	372	520	322	404	0.48	-0.21	0.12
*MtPUB22*	193	2724	268	295	3.82*	0.47	0.61
*MtPUB23*	188	72	450	278	-1.38*	1.26*	0.57
*MtPUB24*	420	438	355	439	0.06	-0.24	0.07
*MtPUB25*	296	330	619	797	0.16	1.07*	1.43*
*MtPUB26*	254	168	163	229	-0.60	-0.64	-0.15
*MtPUB27*	125	89	123	102	-0.49	-0.02	-0.30
*MtPUB28*	960	963	1088	1170	0.00	0.18	0.29
*MtPUB29*	689	1078	1388	1378	0.65	1.01*	1.00*
*MtPUB30*	364	232	164	240	-0.65	-1.15*	-0.60
*MtPUB31*	26	23	16	49	-0.14	-0.71	0.91
*MtPUB32*	1314	1815	2248	1965	0.47	0.77	0.58
*MtPUB33*	176	898	367	276	2.35*	1.06*	0.65
*MtPUB34*	247	232	148	191	-0.09	-0.74	-0.37
*MtPUB35*	33	392	158	162	3.57*	2.26*	2.29*
*MtPUB36*	407	413	544	654	0.02	0.42	0.68
*MtPUB37*	526	480	270	270	-0.13	-0.96	-0.96
*MtPUB38*	166	169	138	170	0.02	-0.27	0.04
*MtPUB39*	389	453	432	579	0.22	0.15	0.58
*MtPUB40*	713	720	760	728	0.01	0.09	0.03
*MtPUB41*	413	348	273	399	-0.25	-0.60	-0.05
*MtPUB42*	37	222	684	449	2.58*	4.20*	3.60*
*MtPUB43*	17	68	23	8	2.05*	0.49	-1.04*
*MtPUB44*	274	2081	506	532	2.93*	0.89	0.96
*MtPUB45*	3	17	7	5	2.49*	1.20*	0.76
*MtPUB46*	212	215	218	229	0.02	0.04	0.11
*MtPUB47*	1392	1537	1539	1790	0.14	0.14	0.36
*MtPUB48*	62	148	50	25	1.26*	-0.31	-1.32*
*MtPUB49*	350	269	237	525	-0.38	-0.56	0.58
*MtPUB50*	174	194	174	148	0.16	0.00	-0.23
*MtPUB51*	121	136	143	152	0.17	0.24	0.33
*MtPUB52*	792	937	775	958	0.24	-0.03	0.27
*MtPUB53*	1	1	1	1	0	0	0
*MtPUB54*	205	226	195	219	0.14	-0.07	0.10
*MtPUB55*	95	949	227	77	3.32*	1.25*	-0.31
*MtPUB56*	298	626	925	467	1.07*	1.64*	0.65
*MtPUB57*	874	1148	1370	1357	0.39	0.65	0.63
*MtPUB58*	255	250	261	297	-0.03	0.03	0.22
*MtPUB59*	278	358	353	385	0.37	0.34	0.47
*MtPUB60*	1	1	1	1	0	0	0
*MtPUB61*	198	432	337	378	1.12*	0.77	0.93
*MtPUB62*	1146	891	2446	2168	-0.36	1.09*	0.92
*MtPUB63*	160	188	170	100	0.23	0.08	-0.67
*MtPUB64*	4	4	2	8	0.00	-0.81	0.96

Values indicate number of reads.

* indicates a significant difference in expression compared to the 0 h time point (*P* < 0.01 and |log_2_N| ≥ 1). Cold-0, Cold-2, Cold-6, and Cold-12 indicate 0, 2, 6, and 12 h cold treatment, respectively.

As indicated above, fewer *MtPUB* genes were down-regulated under the analyzed stress conditions. Under drought treatment, *MtPUB2*, *MtPUB3*, *MtPUB4*, *MtPUB6*, *MtPUB9*, *MtPUB11*, *MtPUB20*, *MtPUB27*, *MtPUB40*, *MtPUB46*, and *MtPUB63* were down-regulated (Fig 5B, Table 2). (A gene was considered down-regulated if its expression was decreased at 2, 6, and 12 h and if the log2 fold change < -1 for at least one of these time points). Using the same criteria, *MtPUB2*, *MtPUB3*, *MtPUB4*, *MtPUB40*, *MtPUB46*, and *MtPUB49* were down-regulated under salt stress (Table 3). After cold treatment, only *MtPUB5* and *MtPUB30* were down-regulated. We also identified *MtPUB* genes that were induced by more than one stress condition (Fig 5). For example, *MtPUB10*, *MtPUB17*, *MtPUB18*, *MtPUB35*, *MtPUB42*, and *MtPUB44* were induced by salt, drought, and cold treatment. In addition, *MtPUB2*, *MtPUB3*, *MtPUB4*, *MtPUB40*, and *MtPUB46* were down-regulated under salt stress and under drought stress (Fig 5).

**Fig 6:**
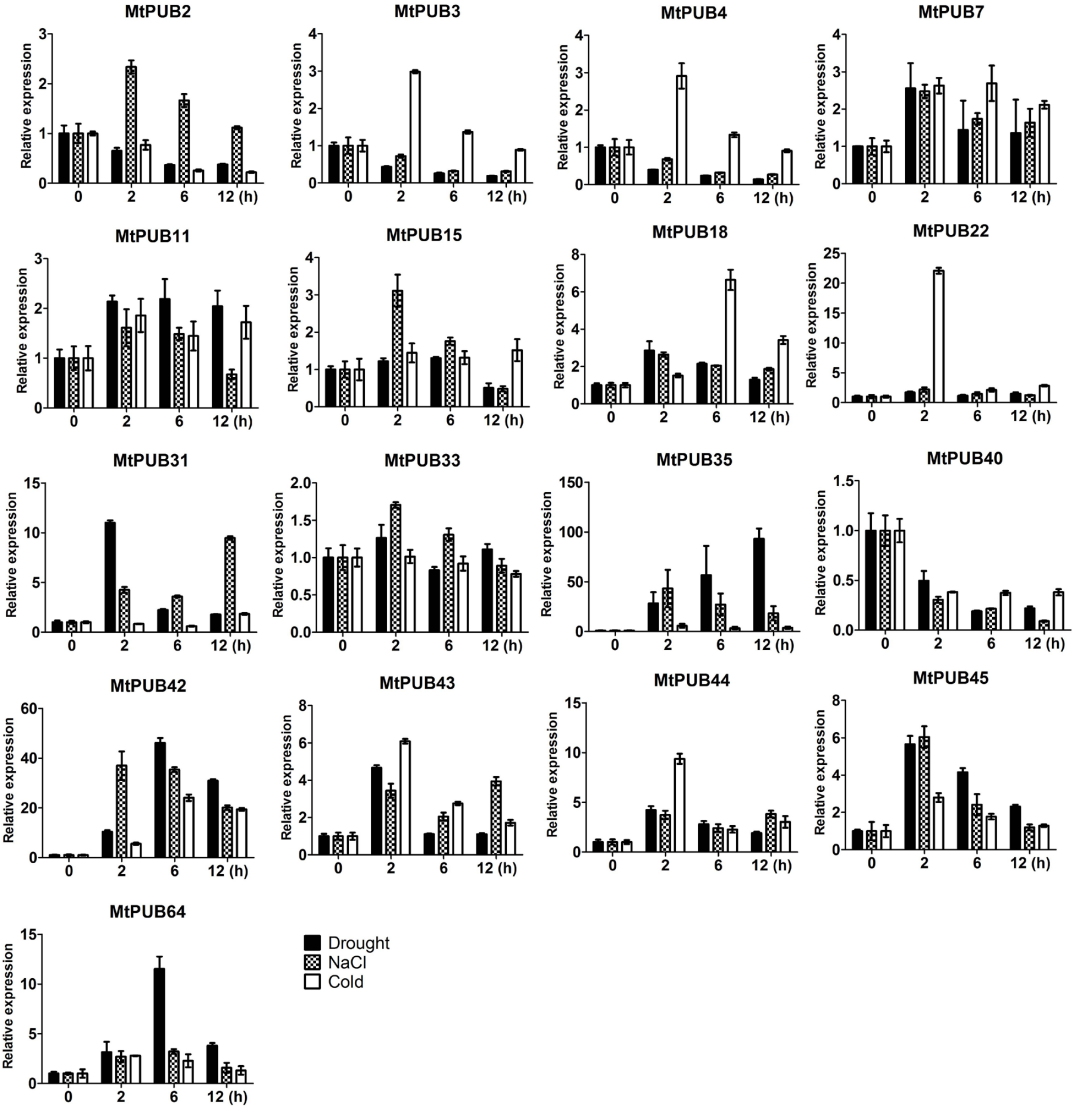
The expression of U-box protein-encoding genes induced by drought, salt, and cold stress as determined by qRT-PCR. Four-week-old seedlings were treated with drought (by transferring them to dry Whatman 3MM paper in a sterile petri dish), NaCl (300 mM), or cold (4°C) for 0, 2, 6, and 12 h.

To verify the above data, we conducted qRT-PCR to examine the expression patterns of 17 *MtPUB* genes under the different stress conditions (Fig 6 and S3 Table). Under drought stress, the transcript levels of the following U-box protein-encoding genes increased: *MtPUB7*, *MtPUB11*, *MtPUB18*, *MtPUB22*, *MtPUB31*, *MtPUB35*, *MtPUB42*, *MtPUB43*, *MtPUB44*, *MtPUB45*, and *MtPUB64*. Among these, *MtPUB31*, *MtPUB35*, *MtPUB42*, *MtPUB43*, *MtPUB44*, *MtPUB45*, and *MtPUB64* were strongly induced. *MtPUB35*, *MtPUB42*, *MtPUB43*, *MtPUB44*, and *MtPUB45* were also strongly induced by salt stress treatment. Under cold stress, the transcript levels of *MtPUB3*, *MtPUB4*, *MtPUB22*, *MtPUB35*, *MtPUB42*, *MtPUB43*, *MtPUB44*, *MtPUB45*, and *MtPUB64* increased, and among these, *MtPUB18*, *MtPUB22*, *MtPUB35*, *MtPUB42*, *MtPUB43*, and *MtPUB44* were strongly induced. It is worth noting that the domain analysis identified MtPUB35 and MtPUB42 as U-box-ARM proteins and that U-box-ARM proteins in *Arabidopsis* and rice are known to have important roles in plant stress response [15].

A few *MtPUBs* were down-regulated under stress, including *MtPUB40*, which was down-regulated under all three abiotic stress conditions. *MtPUB3* and *MtPUB4* were down-regulated under drought and salt stress, and *MtPUB2* was down-regulated under drought and cold stress. These data illustrate the consistency between the qRT-PCR and high-throughput sequencing analyses (Fig 5 and Fig 6, Tables 2–4). Some U-box protein-encoding genes were induced by all three stress conditions and may therefore have important roles in response to abiotic stress; however, further study is required to characterize the functions of these and other *MtPUB* genes.

### Stress-associated *cis*-acting elements in *MtPUB* promoters

*Cis*-regulatory elements and *trans*-acting factors involved in stress-induced gene expression have been extensively analyzed [7]. To identify promoter elements at *MtPUB* loci, we analyzed the 1500 bp upstream promoter sequences of the 64 *MtPUBs* using the PlantCARE database (http://intra.psb.ugent.be:8080/PlantCARE) [34]. The elements listed in S2 Table include several known stress-related elements, including the MYB binding site involved in drought inducibility (MBS), anaerobic induction elements (AREs), heat-stress-responsive elements (HSEs), low-temperature-responsive elements (LTRs), ABA-responsive elements (ABREs), and stress-responsive elements (TC-rich repeats) and so on [35,36]. Among the 64 *MtPUBs*, 27 had ABREs, suggesting they might be involved in ABA-mediated stress response processes. Forty-five *MtPUBs* had AREs, elements involved in the response to hypoxic, low-temperature, and dehydration stresses [37]. The presence of ABREs and AREs in some *MtPUBs* suggests that they might be regulated by stress conditions. For example, we found more than two AREs and ABREs in the promoters of *MtPUB13*, *MtPUB17*, *MtPUB42*, *MtPUB48*, and *MtPUB57*. These findings from the analysis of stress-responsive *cis* elements provide auxiliary evidence that some *MtPUBs* are likely to be involved in the response to abiotic stresses.

## Discussion

### 2.1 U-box family genes structure and evolution

The global identification of U-box genes should help improve the understanding of gene expression and regulatory mechanisms that underlie plant tolerance to abiotic stresses such as salinity, drought, and cold. This study identified 64 U-box genes from *M*. *truncatula*, which is similar to the number identified in *Arabidopsis* (61) (S1 Table) [38] and rice (77) (S1 Table) [5]. Compared to higher plants, there are far fewer U-box proteins in yeast (3) and human (20) [39], indicating an uneven distribution of U-box proteins among species of different kingdoms. Considering the percentage of U-box genes among total genes in the genome, the percentage in *M*. *truncatula* (0.134%) was lower than that in *Arabidopsis* (0.249%). Through the phylogenetic tree analysis, we found that multiple members in each class of U-box proteins raised the possibility of functional redundancy among the members, such as *MtPUB10* and *MtPUB11* (Fig 2). Such functional redundancy may represent a daunting challenge for the functional characterization of *PUB* genes.

In addition to the U-box domain, other important domains, including the ARM, kinase, KAP, and WD40 domains, were present in the identified proteins. The most highly represented was the ARM domain, an approximately 40-amino-acid long tandemly repeated sequence motif (Fig 1). This domain was first identified in the *Drosophila melanogaster* segment polarity protein Armadillo, which is involved in Wingless signal transduction [40]. Structural characteristics of the ARM motif suggest its involvement in protein-protein interaction, which has been demonstrated in several cases [41]. In a few cases, HEAT repeats were detected in proximity to the ARM repeats. In animals, the functions of ARM-repeat proteins are significant, including cytoskeletal regulation and intracellular signaling transduction.

We analyzed the chromosomal locations of the U-box protein-encoding genes on the *M*. *truncatula* genome (Fig 3). Profiling of the gene distribution on the eight *M*. *truncatula* chromosomes indicated that the gene family evolved in this species through a large number of duplication events. Gene duplication was defined according to the following criteria: (1) The length of the sequence alignment covered ≥80% of the longest gene, and (2) the similarity of the aligned gene regions was ≥70% [22,23]. The 64 U-box genes in *M*. *truncatula* were distributed on all eight chromosomes, but in some cases, the genes were concentrated in certain chromosomal regions, such as the bottom half of chromosome 1. In addition, we found some U-box genes were arranged in tandem repeats of two genes, representative of local gene duplications. This finding suggests that tandem duplications of chromosomal regions may have played an important role in the expansion of this gene family. On the other hand, we also found tandem U-box genes harboring different functional domains, indicative of diversification by domain shuffling after tandem duplication, which would promote functional diversity of the U-box genes.

### 2.2 U-box family genes tissue-differentially expression and function

The functions of U-box genes in *M*. *truncatula* remain poorly understood. Some of them were constitutively expressed, such as *MtPUB25*, *MtPUB52*, *MtPUB56*, and *MtPUB58*, whose high expression levels in tissues suggest they may be essential for *M*. *truncatula* growth and development (Fig 4). Other U-box genes, such as *MtPUB42*, had low expression levels in all tissues but were clearly induced by stress according to the RNA-seq data, indicating a potential role in abiotic stress. Finally, tissue-specific expression was also observed, such as the root-specific expression of *MtPUB49*, indicating that some U-box genes may have tissue-specific or organ-specific functions (Fig 4).

### 2.3 U-box family genes in response to various abiotic stresses

It remains unclear why plants have more U-box proteins than other organisms. One possibility is that U-box proteins significantly contribute to the ability of plants to respond to diverse environmental stresses, due to plant immobility and the lack of an animal-like immune system [39]. There has been increasing evidence supporting this hypothesis in recent years, which prompted us to investigate whether *M*. *truncatula* PUB proteins are induced by abiotic stress. The number of up-regulated U-box genes was 15, 25, and 16 under drought, salt, and cold stress, respectively. In contrast, the number of down-regulated U-box genes was 11, 6, and 3, respectively (Fig 5, Tables 2–4). Thus, abiotic stress mainly induces U-box gene expression. Many genes were induced by two or three stress conditions and may therefore play a role under various environmental stresses. Our results showed that, as in other species, the expression of many *MtPUB* genes, such as *MtPUB10*, *MtPUB17*, *MtPUB18*, *MtPUB35*, *MtPUB42*, and *MtPUB44*, could be induced by drought, salt, and cold stress (Fig 5, Tables 2–4).

In higher plants, U-box-ARM proteins have been implicated in the regulation of cell death and defense [9] and in reducing cellular oxidative stress during seedling establishment in rice [15]. *MtPUB35* and *MtPUB42* were found to encode ARM domain-containing proteins and were up-regulated more than 10-fold at different time points under all three stresses (Fig 5, Tables 2–4). In addition to their classification as U-box-ARM protein-encoding genes with markedly induced expression under abiotic stresses, the proteins encoded by *MtPUB35* and *MtPUB42* were grouped together in the G1 subfamily in the phylogenetic analysis. Analysis of *cis* sequences revealed 4 and 3 ABRE elements in *MtPUB35* and *MtPUB42*, respectively, as well as 5 ARE elements in *MtPUB42* (S2 Table), further indicating that the two U-Box-ARM genes are important for stress response. Further study of these genes is therefore warranted. In short, these results are consistent with the findings in other plants that U-box-ARM proteins have the potential to regulate plant responses to abiotic stresses. *M*. *truncatula* homologs of other characterized *PUB* genes were also identified in the present study. For example, the *Arabidopsis* genes *AtPUB22* and *AtPUB23* play a key role in drought stress response [10], so *MtPUB18*, the homologous gene in *M*. *truncatula*, may also be associated with drought stress. Similarly, *MtPUB44* may be involved in disease resistance, as it is homologous to tobacco *NtCMPG1*, which has been shown to be essential for disease resistance [42]. Taken together, the present findings suggest that PUB proteins likely play critical roles in stress response in *M*. *truncatula*.

## Supporting information

S1 Fig A phylogenetic tree of U-Box protein (Pub) family from 3 species (Mt,At,Os). (PDF)

S2 Fig Abundance of transcriptions in stress treatment vs. non-stress treatment samples. (PDF)

S3 Fig Abundance of transcriptions between two samples. (PDF)

S1 Table U-box protein-encoding genes in Medicago truncatula, Arabidopsis thaliana, and Oryza sativa. Detailed genomic information, including the gene name, gene ID, and protein sequence, is provided for each U-box gene.
(XLS)

S2 Table 15 types of cis-acting elements and the number of times they occurred in each U-box protein-encoding gene. (XLS)

S3 Table Primer sequences used for this study. (XLS)

## References

[1] Ciechanover A (1998) The ubiquitin–proteasome pathway: on protein death and cell life. The EMBO journal 17: 7151–7160. doi: 10.1093/emboj/17.24.7151 9857172 10.1093/emboj/17.24.7151 PMC1171061

[2] Ciechanover A (2005) Intracellular protein degradation: from a vague idea thru the lysosome and the ubiquitin–proteasome system and onto human diseases and drug targeting. Cell Death & Differentiation 12: 1178–1190. 16094394 10.1038/sj.cdd.4401692

[3] Yee D, Goring DR (2009) The diversity of plant U-box E3 ubiquitin ligases: from upstream activators to downstream target substrates. Journal of Experimental Botany: ern369. 10.1093/jxb/ern369 19196749

[4] Koegl M, Hoppe T, Schlenker S, Ulrich HD, Mayer TU, Jentsch S (1999) A novel ubiquitination factor, E4, is involved in multiubiquitin chain assembly. Cell 96: 635–644. 10089879 10.1016/s0092-8674(00)80574-7

[5] Zeng L-R, Park CH, Venu R, Gough J, Wang G-L (2008) Classification, expression pattern, and E3 ligase activity assay of rice U-box-containing proteins. Molecular Plant 1: 800–815. doi: 10.1093/mp/ssn044 19825583 10.1093/mp/ssn044

[6] Hatakeyama S, Yada M, Matsumoto M, Ishida N, Nakayama K-I (2001) U box proteins as a new family of ubiquitin-protein ligases. Journal of Biological Chemistry 276: 33111–33120. doi: 10.1074/jbc.M102755200 11435423 10.1074/jbc.M102755200

[7] Cho SK, Bae H, Ryu MY, Yang SW, Kim WT (2015) PUB22 and PUB23 U-BOX E3 ligases directly ubiquitinate RPN6, a 26S proteasome lid subunit, for subsequent degradation in Arabidopsis thaliana. Biochemical and biophysical research communications 464: 994–999. doi: 10.1016/j.bbrc.2015.07.030 26188517 10.1016/j.bbrc.2015.07.030

[8] Yan J, Wang J, Li Q, Hwang JR, Patterson C, Zhang H (2003) AtCHIP, a U-box-containing E3 ubiquitin ligase, plays a critical role in temperature stress tolerance in Arabidopsis. Plant Physiology 132: 861–869. doi: 10.1104/pp.103.020800 12805616 10.1104/pp.103.020800 PMC167026

[9] Yang C-W, González-Lamothe R, Ewan RA, Rowland O, Yoshioka H, Shenton M, et al (2006) The E3 ubiquitin ligase activity of Arabidopsis PLANT U-BOX17 and its functional tobacco homolog ACRE276 are required for cell death and defense. The Plant Cell 18: 1084–1098. doi: 10.1105/tpc.105.039198 16531496 10.1105/tpc.105.039198 PMC1425844

[10] Cho SK, Ryu MY, Song C, Kwak JM, Kim WT (2008) Arabidopsis PUB22 and PUB23 are homologous U-Box E3 ubiquitin ligases that play combinatory roles in response to drought stress. The Plant Cell 20: 1899–1914. doi: 10.1105/tpc.108.060699 18664614 10.1105/tpc.108.060699 PMC2518226

[11] Liu Y-C, Wu Y-R, Huang X-H, Sun J, Xie Q (2011) AtPUB19, a U-box E3 ubiquitin ligase, negatively regulates abscisic acid and drought responses in Arabidopsis thaliana. Molecular plant 4: 938–946. doi: 10.1093/mp/ssr030 21502661 10.1093/mp/ssr030 PMC3221247

[12] Li W, Ahn I-P, Ning Y, Park C-H, Zeng L, Whitehill JG, et al (2012) The U-Box/ARM E3 ligase PUB13 regulates cell death, defense, and flowering time in Arabidopsis. Plant physiology 159: 239–250. doi: 10.1104/pp.111.192617 22383540 10.1104/pp.111.192617 PMC3366716

[13] Hwang JH, Seo DH, Kang BG, Kwak JM, Kim WT (2015) Suppression of Arabidopsis AtPUB30 resulted in increased tolerance to salt stress during germination. Plant cell reports 34: 277–289. doi: 10.1007/s00299-014-1706-4 25410251 10.1007/s00299-014-1706-4

[14] Zeng L-R, Qu S, Bordeos A, Yang C, Baraoidan M, Yan H, et al (2004) Spotted leaf11, a negative regulator of plant cell death and defense, encodes a U-box/armadillo repeat protein endowed with E3 ubiquitin ligase activity. The Plant Cell 16: 2795–2808. doi: 10.1105/tpc.104.025171 15377756 10.1105/tpc.104.025171 PMC520972

[15] Park JJ, Yi J, Yoon J, Cho LH, Ping J, Jeong HJ, et al (2011) OsPUB15, an E3 ubiquitin ligase, functions to reduce cellular oxidative stress during seedling establishment. The Plant Journal 65: 194–205. doi: 10.1111/j.1365-313X.2010.04416.x 21223385 10.1111/j.1365-313X.2010.04416.x

[16] Wang J, Qu B, Dou S, Li L, Yin D, Pang Z, et al (2015) The E3 ligase OsPUB15 interacts with the receptor-like kinase PID2 and regulates plant cell death and innate immunity. BMC plant biology 15: 1 doi: 10.1186/s12870-014-0410-4 25849162 10.1186/s12870-015-0442-4 PMC4330927

[17] Hur Y-J, Yi YB, Lee JH, Chung YS, Jung HW, Yun DJ, et al (2012) Molecular cloning and characterization of OsUPS, a U-box containing E3 ligase gene that respond to phosphate starvation in rice (Oryza sativa). Molecular biology reports 39: 5883–5888. doi: 10.1007/s11033-011-1399-5 22201023 10.1007/s11033-011-1399-5

[18] Cho SK, Chung HS, Ryu MY, Park MJ, Lee MM, Bahk Y-Y, et al (2006) Heterologous expression and molecular and cellular characterization of CaPUB1 encoding a hot pepper U-Box E3 ubiquitin ligase homolog. Plant physiology 142: 1664–1682. doi: 10.1104/pp.106.087965 17041029 10.1104/pp.106.087965 PMC1676043

[19] Luo Q, Li Y, Wang W, Fei X, Deng X (2015) Genome-wide survey and expression analysis of Chlamydomonas reinhardtii U-box E3 ubiquitin ligases (CrPUBs) reveal a functional lipid metabolism module. PloS one 10: e0122600 doi: 10.1371/journal.pone.0122600 25822994 10.1371/journal.pone.0122600 PMC4378952

[20] Wiborg J, O'Shea C, Skriver K (2008) Biochemical function of typical and variant Arabidopsis thaliana U-box E3 ubiquitin-protein ligases. Biochemical Journal 413: 447–457. doi: 10.1042/BJ20071568 18393940 10.1042/BJ20071568

[21] Bae H, Kim WT (2014) Classification and interaction modes of 40 rice E2 ubiquitin-conjugating enzymes with 17 rice ARM-U-box E3 ubiquitin ligases. Biochemical and biophysical research communications 444: 575–580. doi: 10.1016/j.bbrc.2014.01.098 24486490 10.1016/j.bbrc.2014.01.098

[22] Yang X, Kalluri UC, Jawdy S, Gunter LE, Yin T, Tschaplinski TJ, et al (2008) The F-box gene family is expanded in herbaceous annual plants relative to woody perennial plants. Plant physiology 148: 1189–1200. doi: 10.1104/pp.108.121921 18775973 10.1104/pp.108.121921 PMC2577272

[23] Gu Z, Cavalcanti A, Chen F-C, Bouman P, Li W-H (2002) Extent of gene duplication in the genomes of Drosophila, nematode, and yeast. Molecular biology and evolution 19: 256–262. 11861885 10.1093/oxfordjournals.molbev.a004079

[24] Zhou ZS, Yang SN, Li H, Zhu CC, Liu ZP, Yang ZM (2013) Molecular dissection of mercury-responsive transcriptome and sense/antisense genes in Medicago truncatula. Journal of hazardous materials 252: 123–131. doi: 10.1016/j.jhazmat.2013.02.011 23500795 10.1016/j.jhazmat.2013.02.011

[25] Smith TF, Gaitatzes C, Saxena K, Neer EJ (1999) The WD repeat: a common architecture for diverse functions. Trends in biochemical sciences 24: 181–185. 10322433 10.1016/s0968-0004(99)01384-5

[26] Das AK, Cohen PT, Barford D (1998) The structure of the tetratricopeptide repeats of protein phosphatase 5: implications for TPR‐mediated protein–protein interactions. The EMBO journal 17: 1192–1199. doi: 10.1093/emboj/17.5.1192 9482716 10.1093/emboj/17.5.1192 PMC1170467

[27] LiD R (2001) WD-repeatproteins: Structurecharacteristics, biologicalfunction, andtheirinvolvementinhumandiseases. CellMolLifeSci 58: 2085–2097.

[28] Löscher M, Fortschegger K, Ritter G, Wostry M, Voglauer R, Schmid JA, et al (2005) Interaction of U-box E3 ligase SNEV with PSMB4, the β7 subunit of the 20 S proteasome. Biochemical Journal 388: 593–603. doi: 10.1042/BJ20041517 15660529 10.1042/BJ20041517 PMC1138967

[29] Ohi MD, Gould KL (2002) Characterization of interactions among the Cef1p-Prp19p-associated splicing complex. Rna 8: 798–815. 12088152 10.1017/s1355838202025050 PMC1370298

[30] Luo J, Shen G, Yan J, He C, Zhang H (2006) AtCHIP functions as an E3 ubiquitin ligase of protein phosphatase 2A subunits and alters plant response to abscisic acid treatment. The Plant Journal 46: 649–657. doi: 10.1111/j.1365-313X.2006.02730.x 16640601 10.1111/j.1365-313X.2006.02730.x

[31] Shen G, Adam Z, Zhang H (2007) The E3 ligase AtCHIP ubiquitylates FtsH1, a component of the chloroplast FtsH protease, and affects protein degradation in chloroplasts. The Plant Journal 52: 309–321. doi: 10.1111/j.1365-313X.2007.03239.x 17714429 10.1111/j.1365-313X.2007.03239.x

[32] Rosser MF, Washburn E, Muchowski PJ, Patterson C, Cyr DM (2007) Chaperone functions of the E3 ubiquitin ligase CHIP. Journal of Biological Chemistry 282: 22267–22277. doi: 10.1074/jbc.M700513200 17545168 10.1074/jbc.M700513200

[33] Sahara N, Murayama M, Mizoroki T, Urushitani M, Imai Y, Takahashi R, et al (2005) In vivo evidence of CHIP up‐regulation attenuating tau aggregation. Journal of neurochemistry 94: 1254–1263. doi: 10.1111/j.1471-4159.2005.03272.x 16111477 10.1111/j.1471-4159.2005.03272.x

[34] Lescot M, Déhais P, Thijs G, Marchal K, Moreau Y, Van de Peer Y, et al (2002) PlantCARE, a database of plant cis-acting regulatory elements and a portal to tools for in silico analysis of promoter sequences. Nucleic acids research 30: 325–327. 11752327 10.1093/nar/30.1.325 PMC99092

[35] Mundy J, Yamaguchi-Shinozaki K, Chua N-H (1990) Nuclear proteins bind conserved elements in the abscisic acid-responsive promoter of a rice rab gene. Proceedings of the National Academy of Sciences 87: 1406–1410. 10.1073/pnas.87.4.1406 PMC53484 2137613

[36] Xu D, Duan X, Wang B, Hong B, Ho T-HD, Wu R (1996) Expression of a late embryogenesis abundant protein gene, HVA1, from barley confers tolerance to water deficit and salt stress in transgenic rice. Plant physiology 110: 249–257. 12226181 10.1104/pp.110.1.249 PMC157716

[37] Dolferus R, Jacobs M, Peacock WJ, Dennis ES (1994) Differential interactions of promoter elements in stress responses of the Arabidopsis Adh gene. Plant Physiology 105: 1075–1087. 7972489 10.1104/pp.105.4.1075 PMC159435

[38] Azevedo C, Santos-Rosa MJ, Shirasu K (2001) The U-box protein family in plants. Trends in plant science 6: 354–358. 11495788 10.1016/s1360-1385(01)01960-4

[39] Patterson C (2002) A new gun in town: the U box is a ubiquitin ligase domain. Science Signaling 2002: pe4–pe4. 10.1126/stke.2002.116.pe4 11805346

[40] Riggleman B, Wieschaus E, Schedl P (1989) Molecular analysis of the armadillo locus: uniformly distributed transcripts and a protein with novel internal repeats are associated with a Drosophila segment polarity gene. Genes & development 3: 96–113. 2707602 10.1101/gad.3.1.96

[41] Huber AH, Nelson WJ, Weis WI (1997) Three-dimensional structure of the armadillo repeat region of β-catenin. Cell 90: 871–882. 9298899 10.1016/s0092-8674(00)80352-9

[42] González-Lamothe R, Tsitsigiannis DI, Ludwig AA, Panicot M, Shirasu K, Jones JD (2006) The U-box protein CMPG1 is required for efficient activation of defense mechanisms triggered by multiple resistance genes in tobacco and tomato. The Plant Cell 18: 1067–1083. doi: 10.1105/tpc.106.040998 16531490 10.1105/tpc.106.040998 PMC1425846

