# Supplementary figures and images for: The U-box family genes in Medicago truncatula: Key elements in response to salt, cold, and drought stresses
Source: PLoS One. 2017 Aug 3;12(8):e0182402. doi: 10.1371/journal.pone.0182402 (PMC5542650; doi:10.1371/journal.pone.0182402)

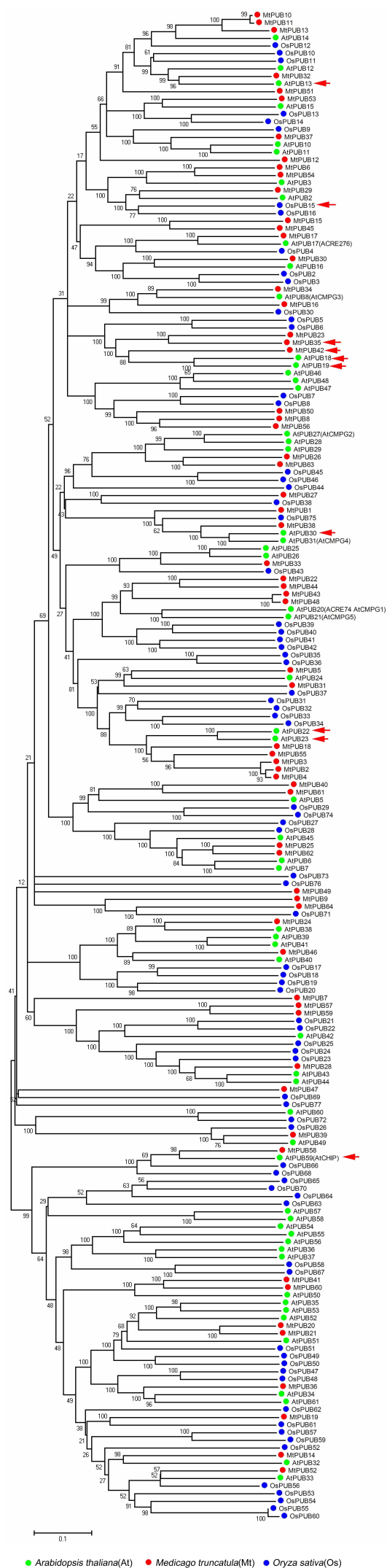

S1 Fig. A phylogenetic tree of U-Box protein (Pub) family from 3 species (Mt, At, Os)

Supplement: S1 Fig — (PDF) [file pone.0182402.s001.pdf]

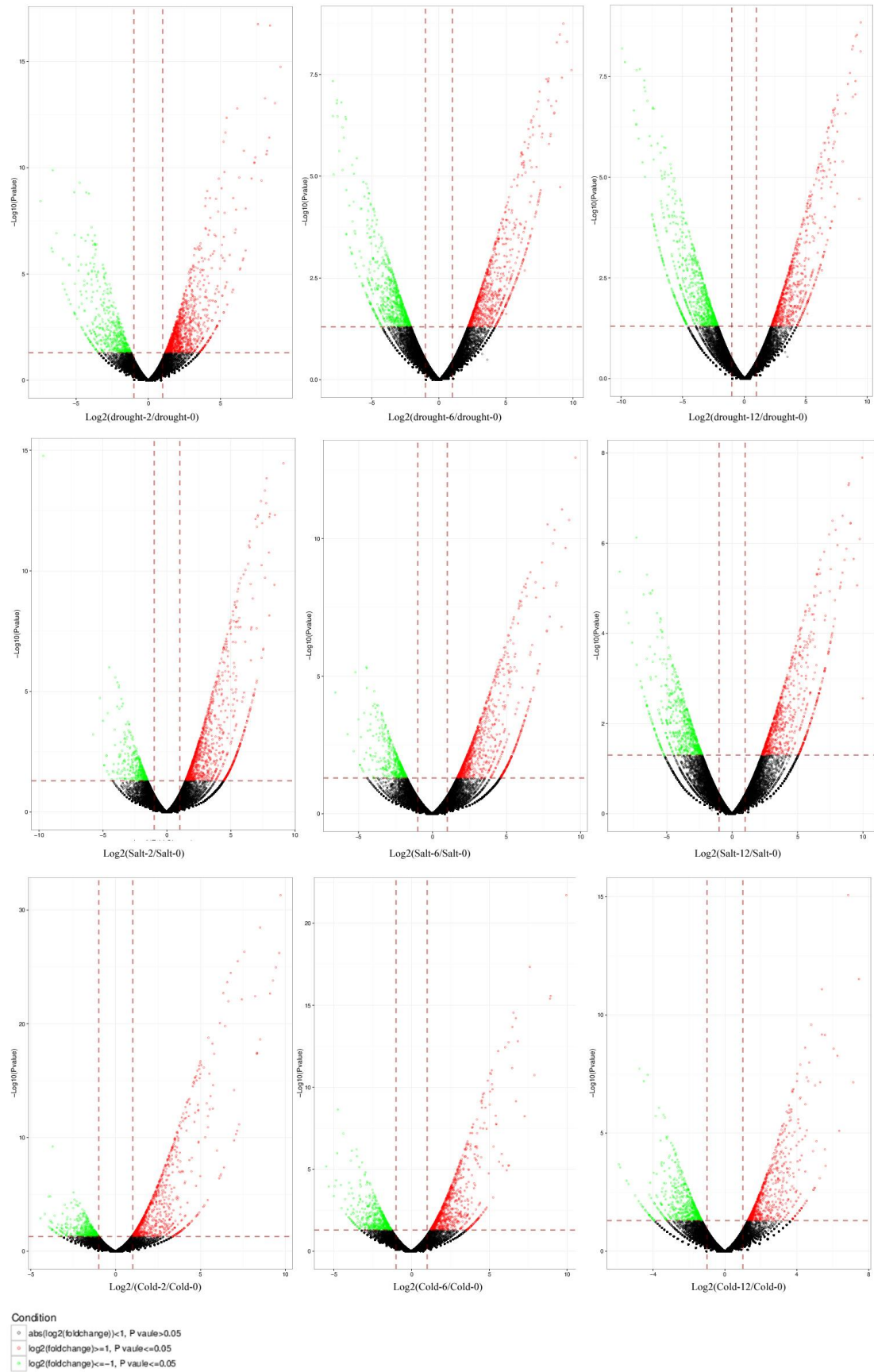

S2 Fig. Abundance of transcriptions in stress treatment vs. non-stress treatment samples

Supplement: S2 Fig — (PDF) [file pone.0182402.s002.pdf]

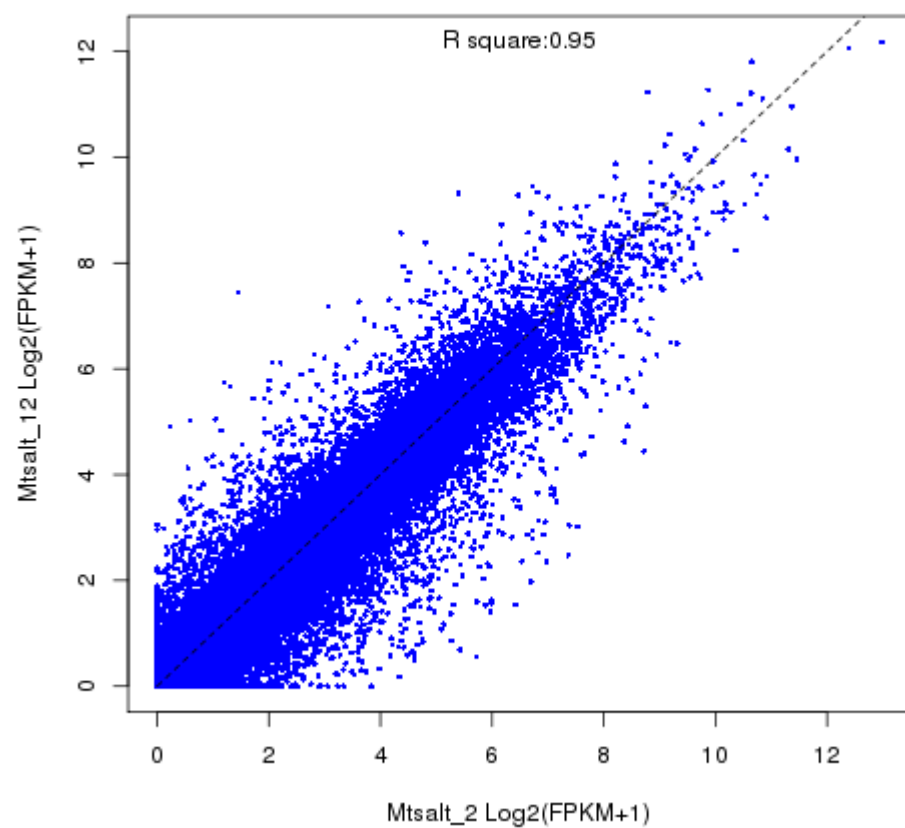

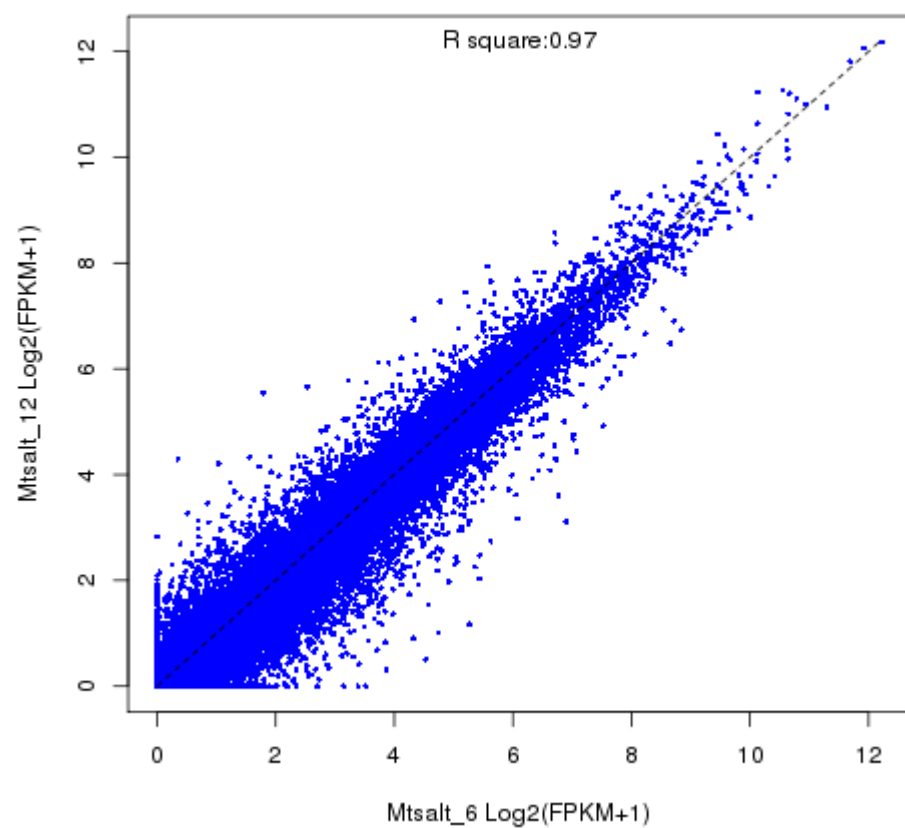

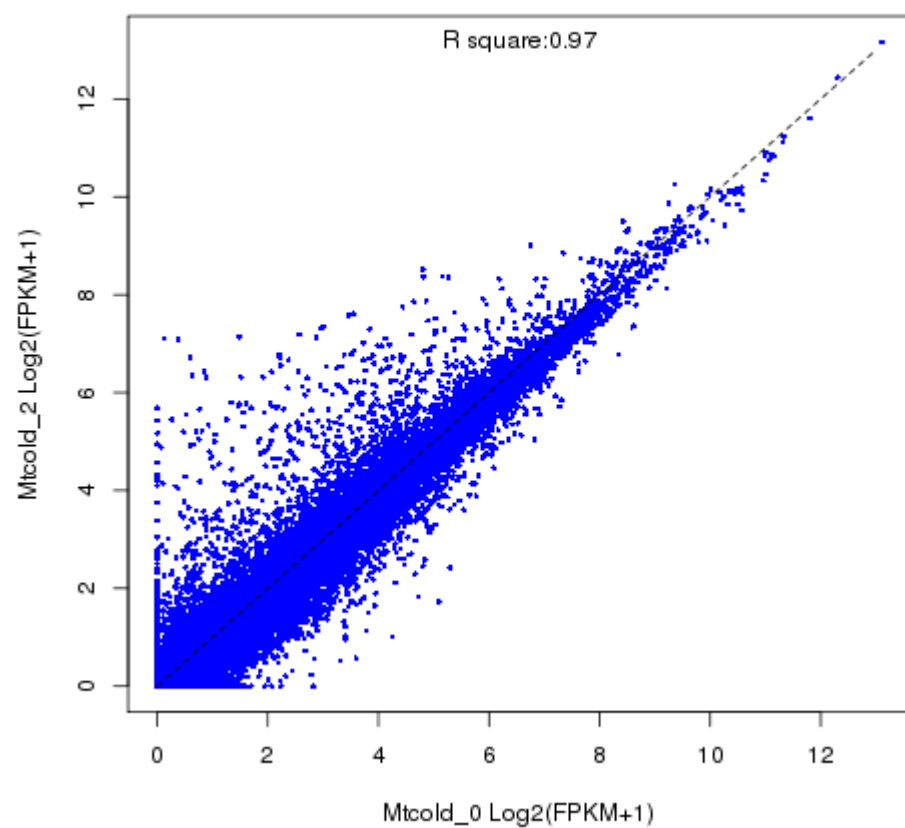

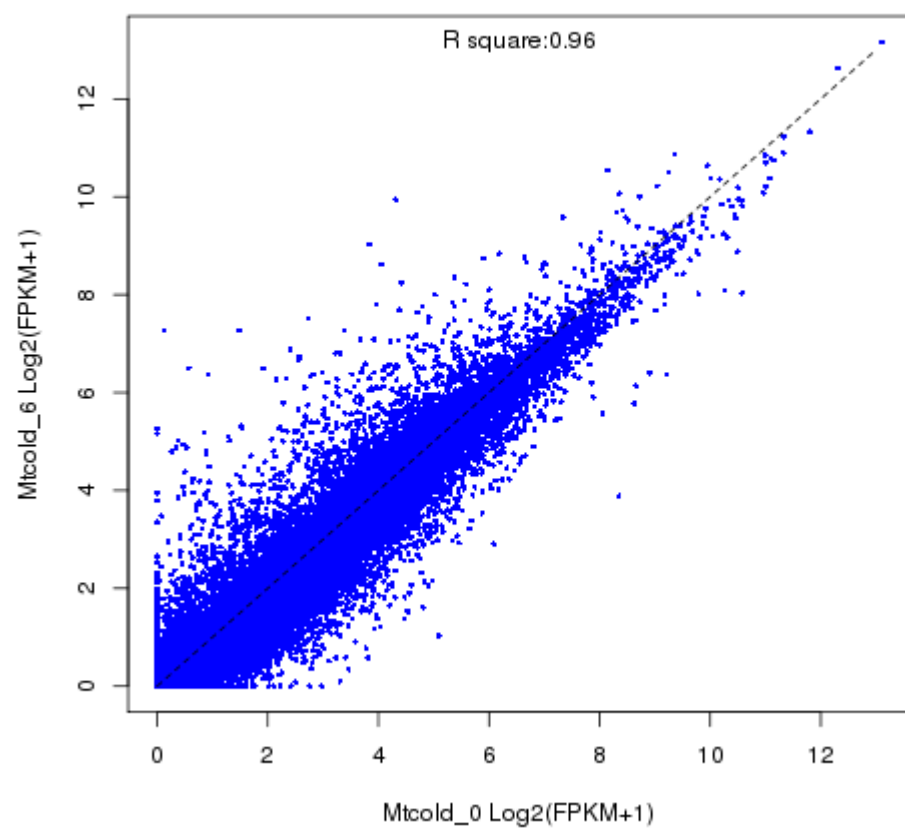

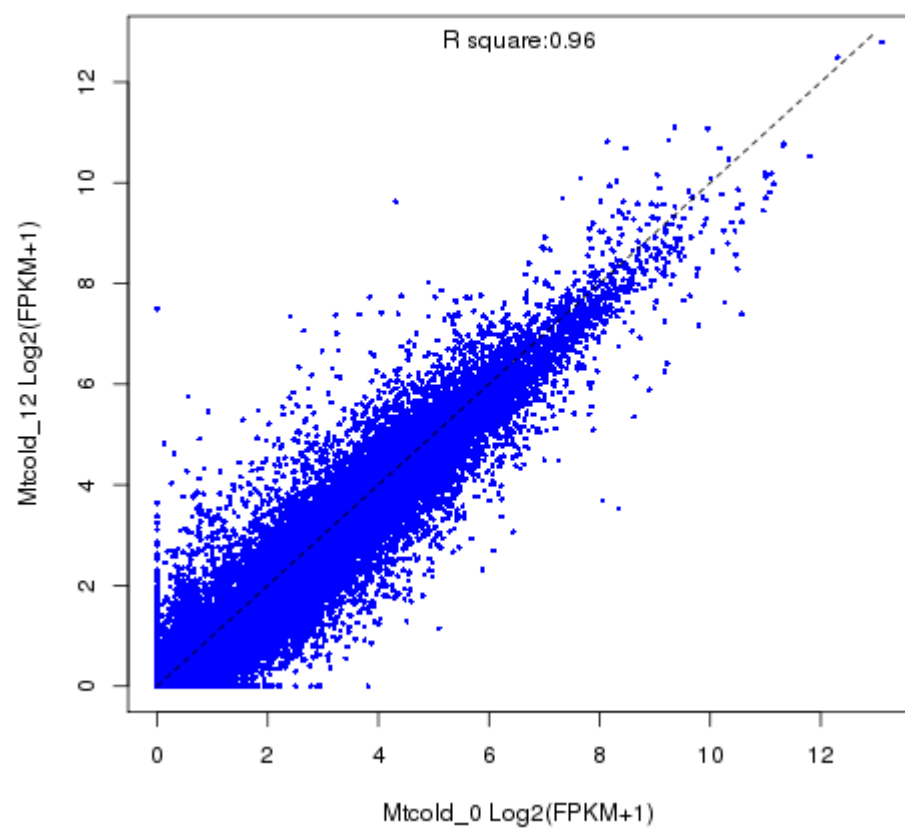

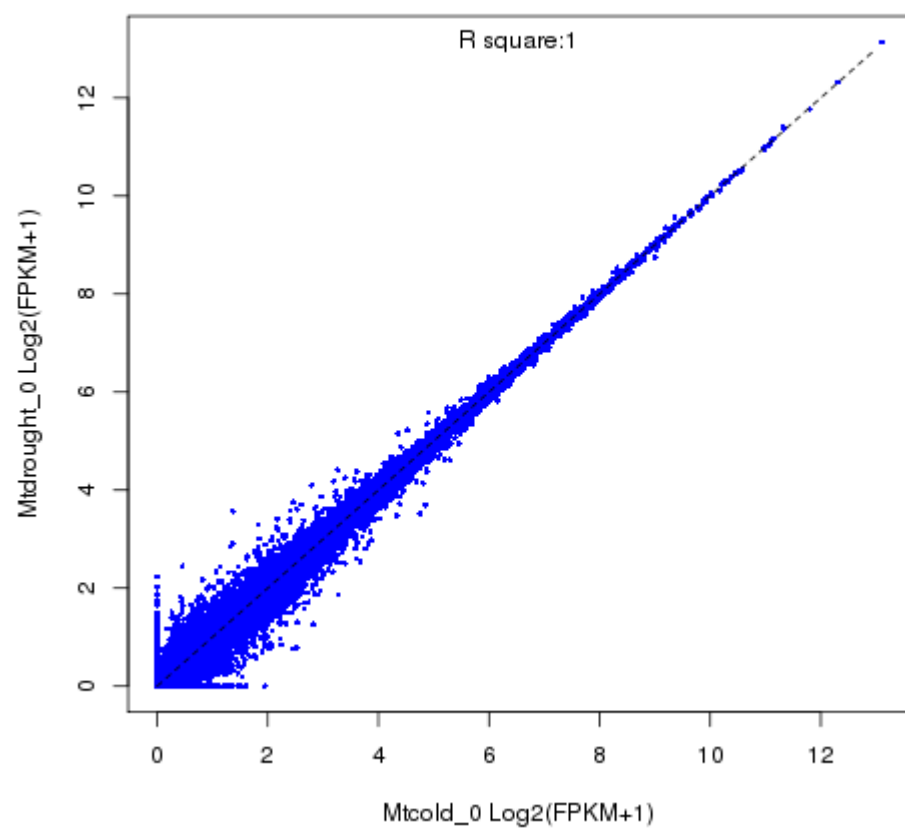

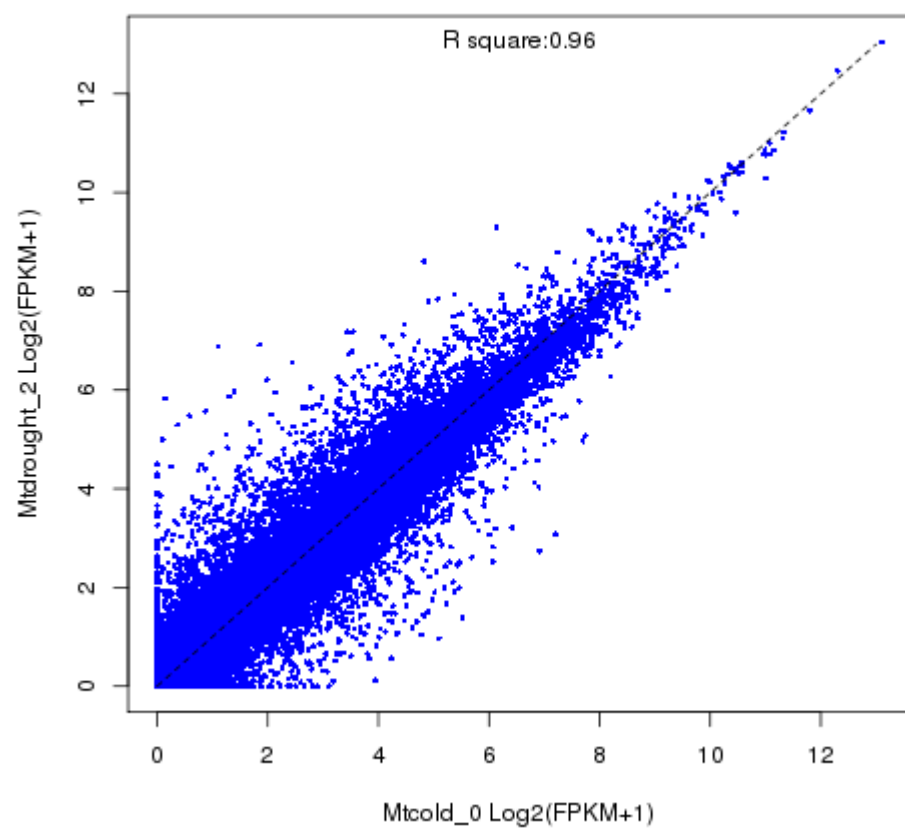

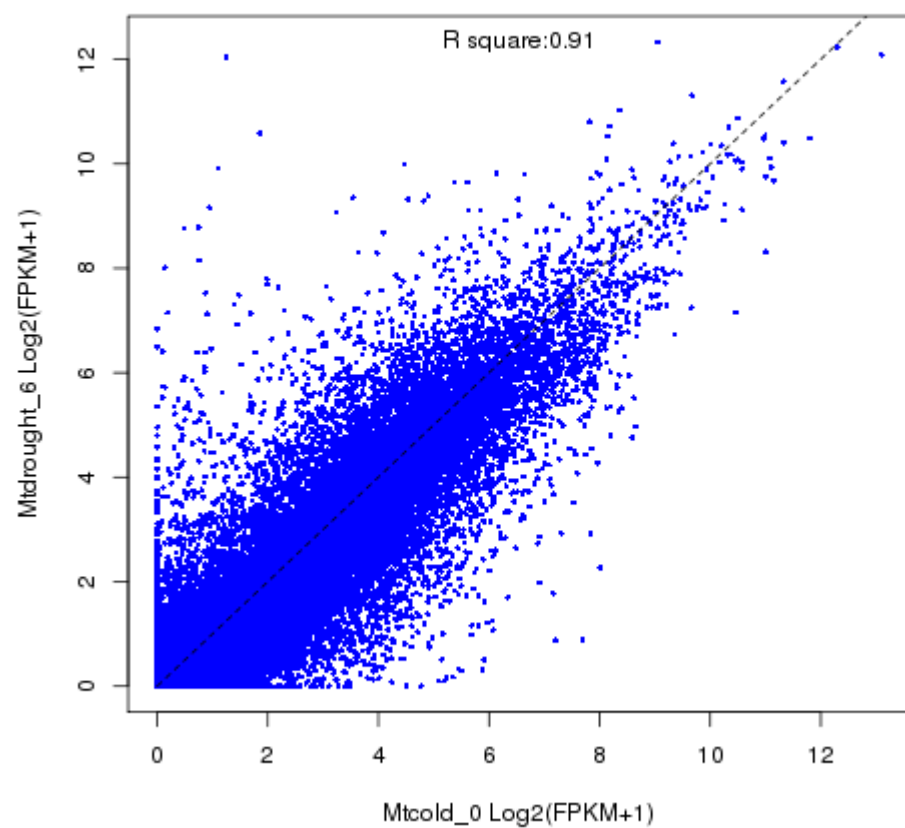

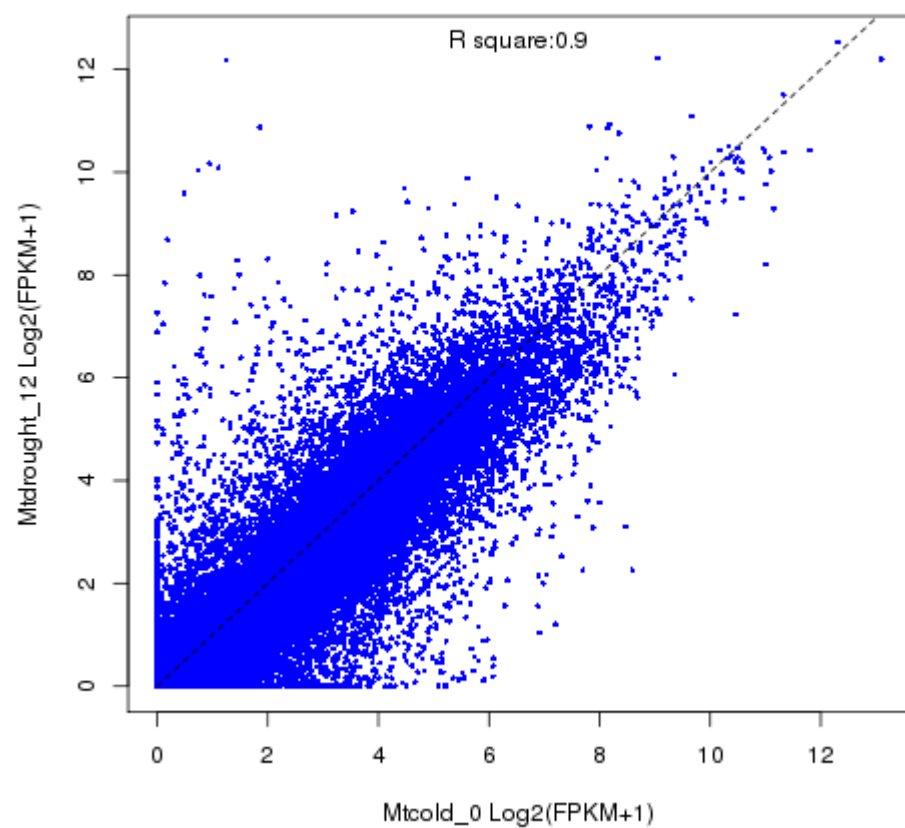

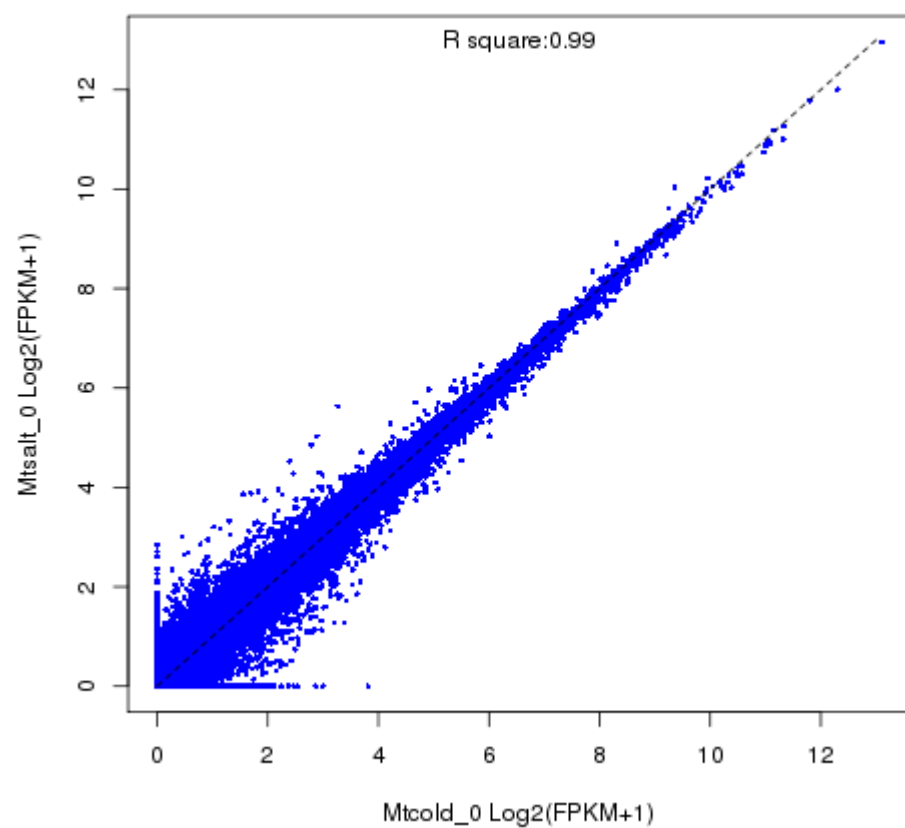

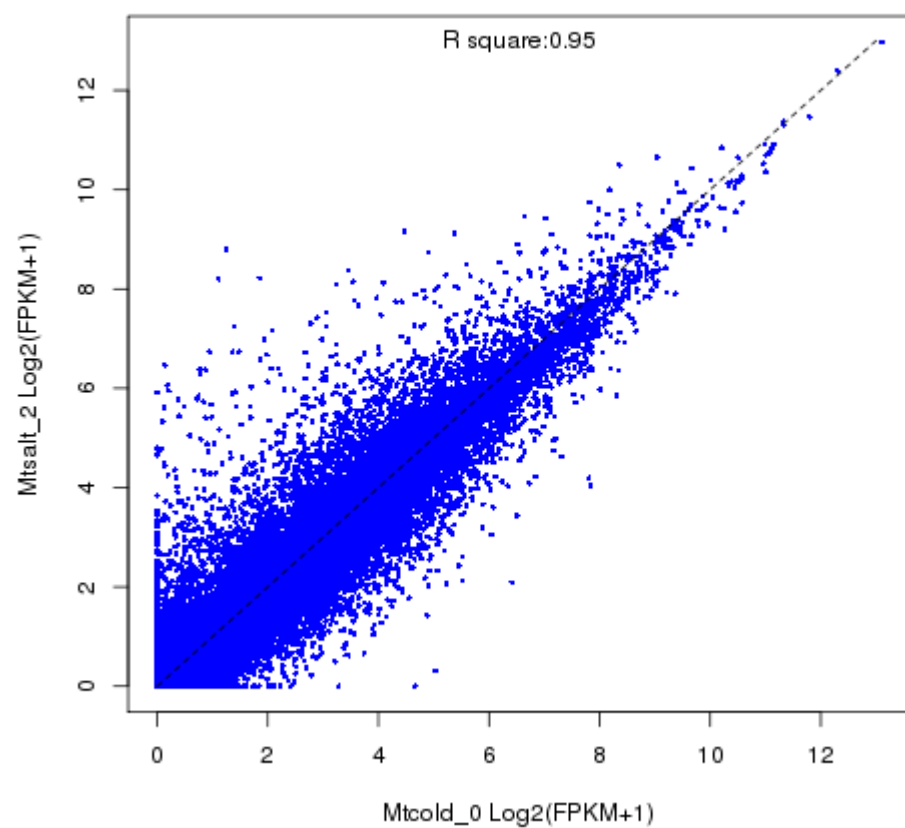

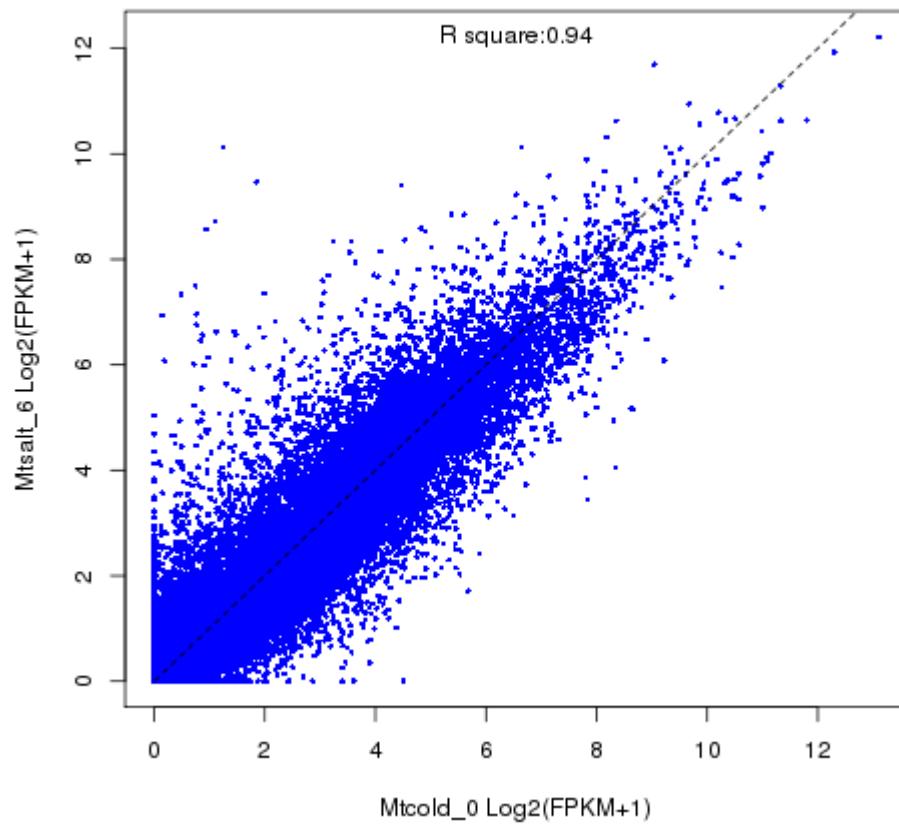

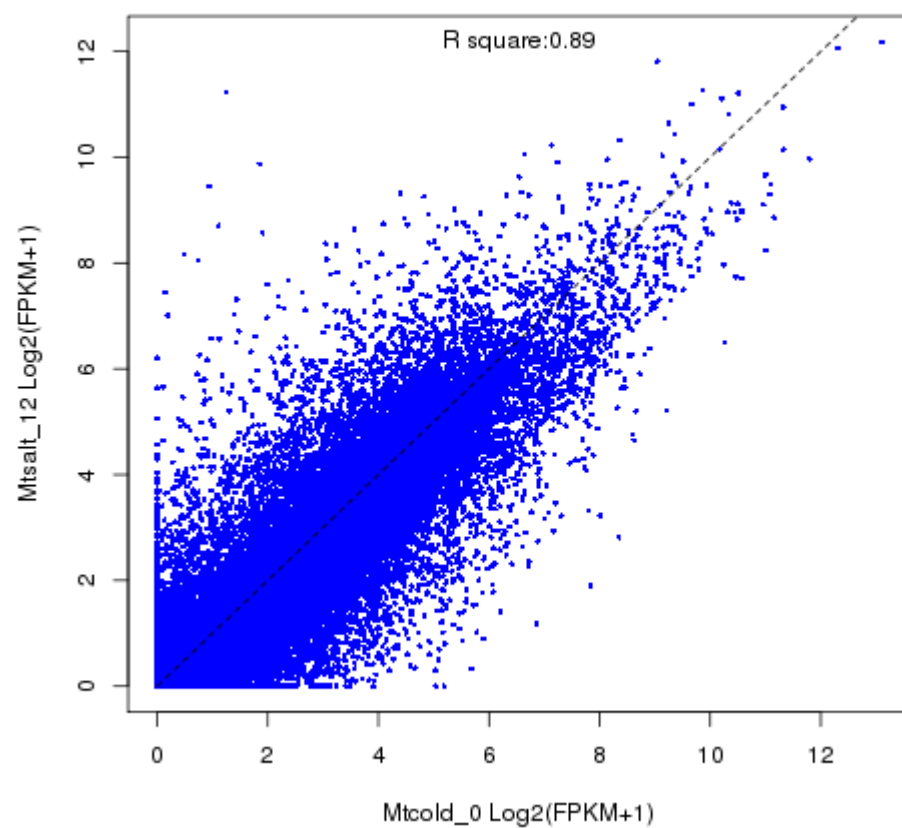

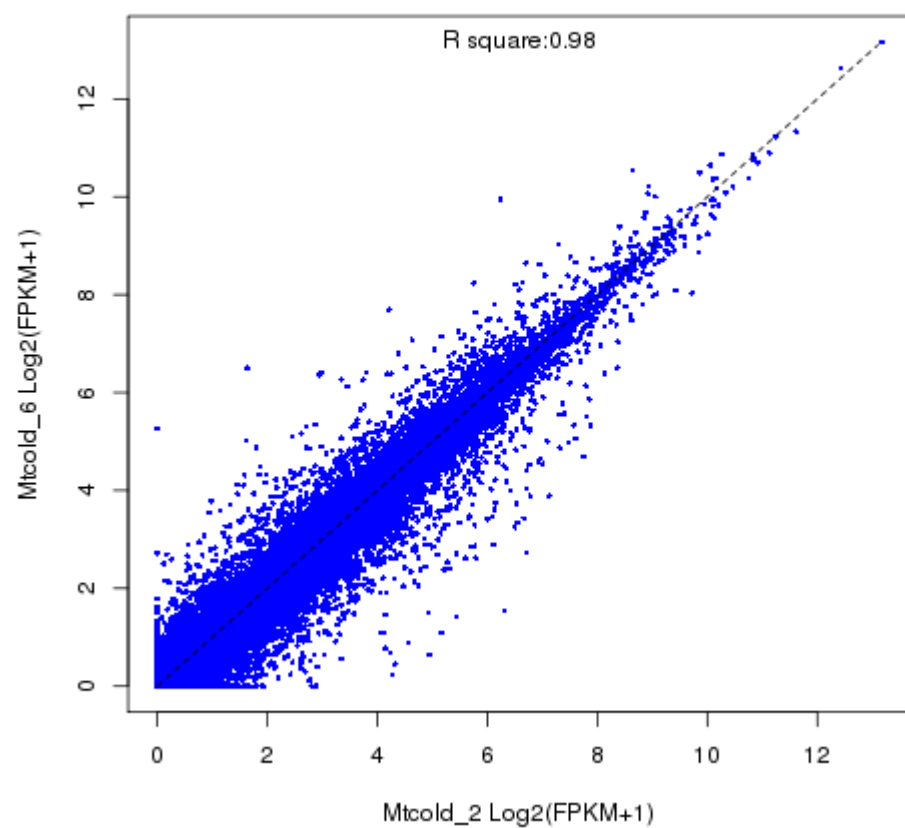

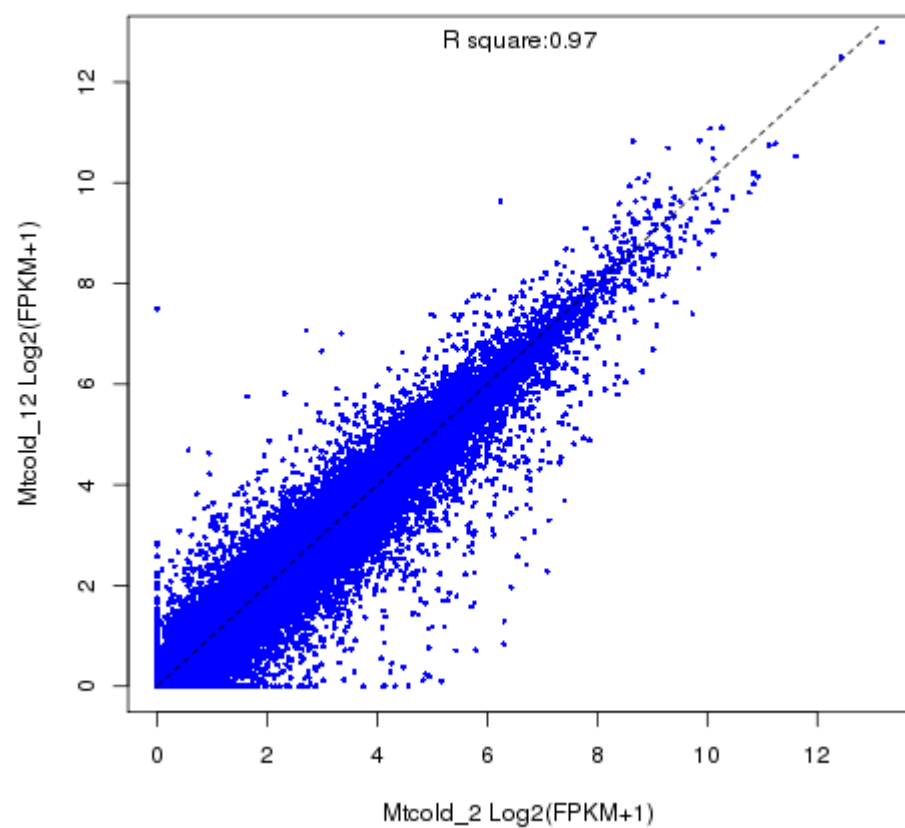

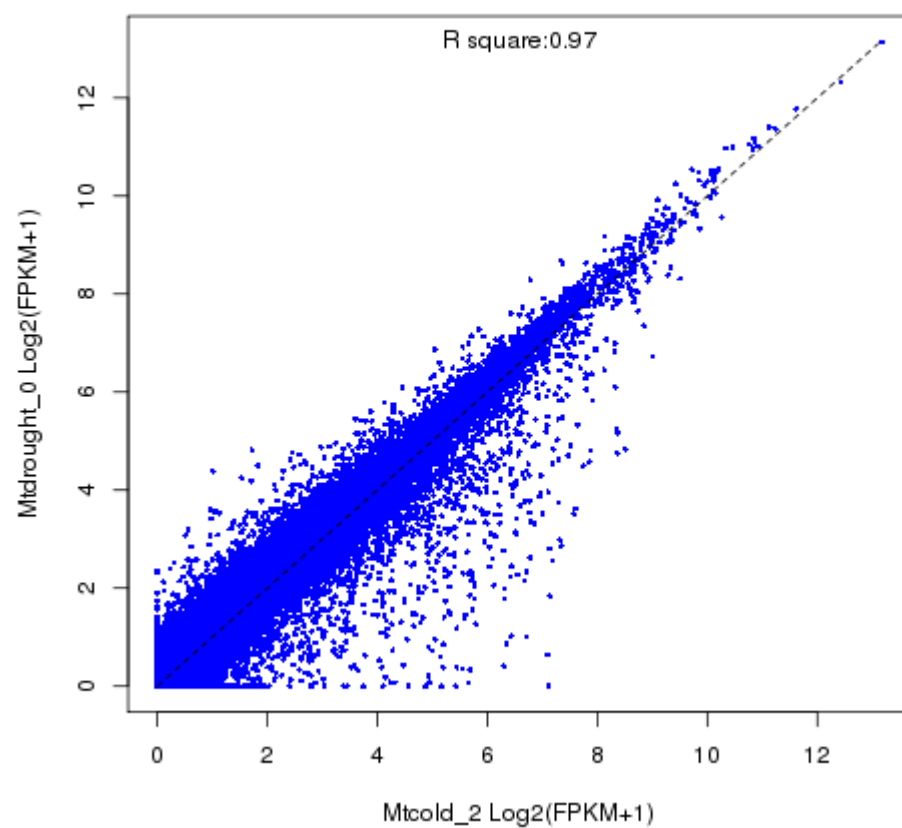

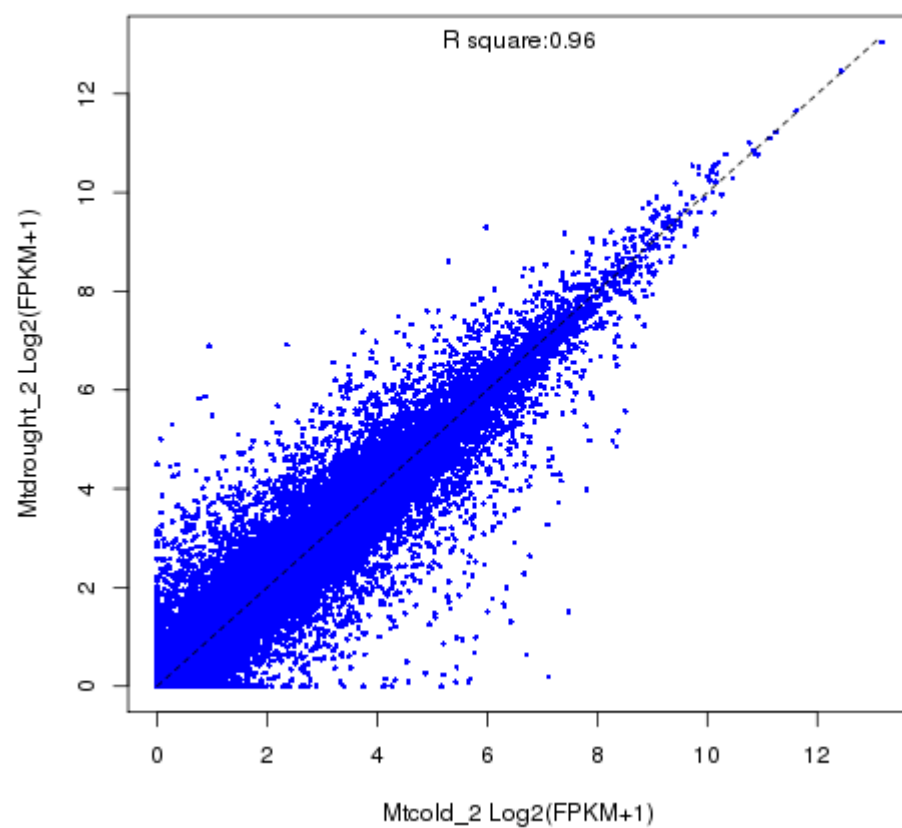

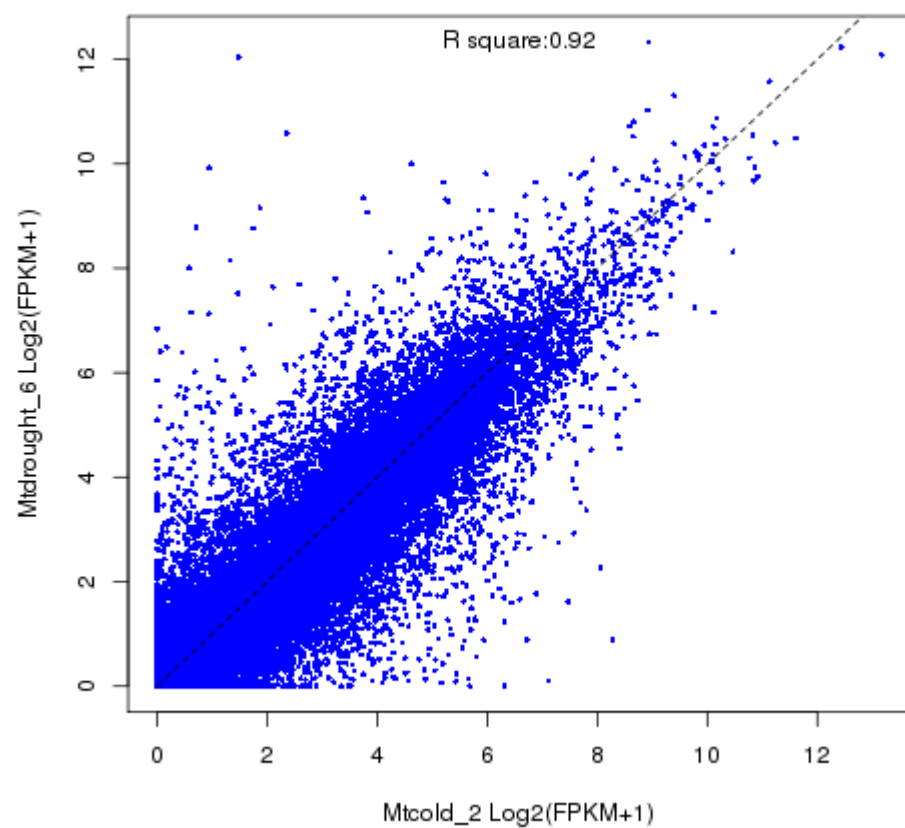

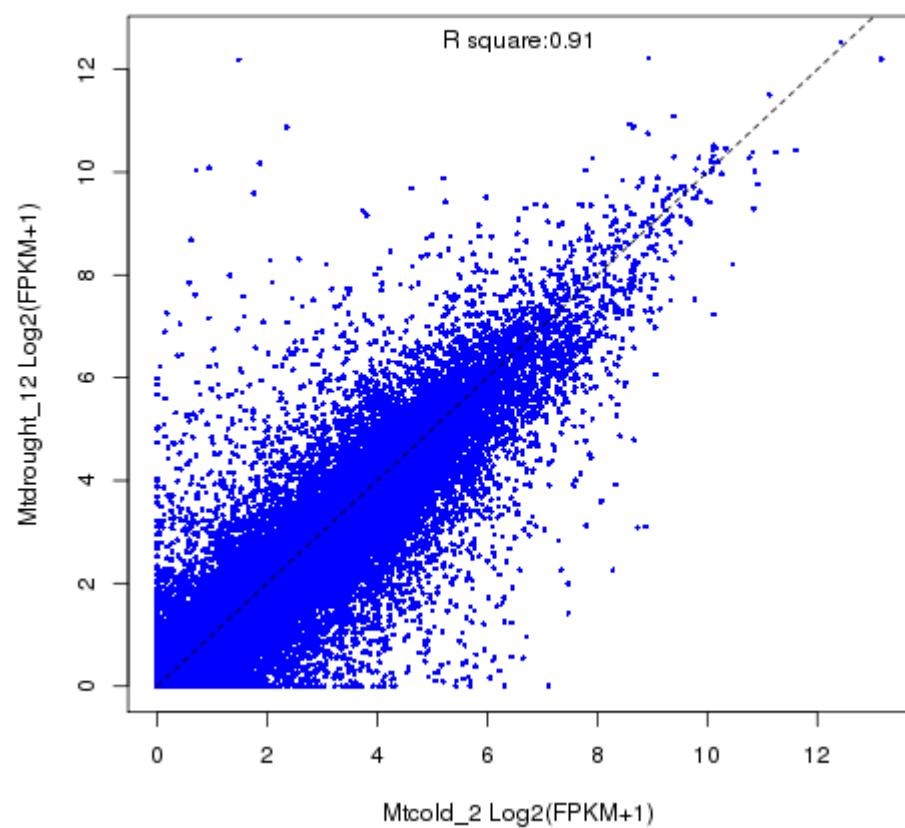

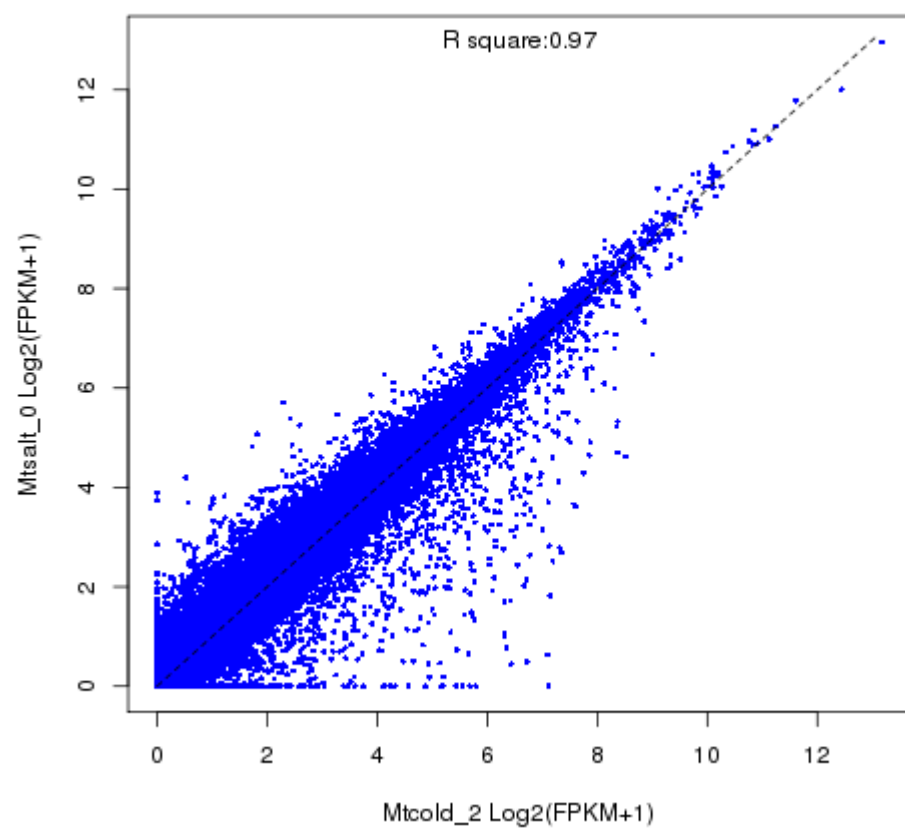

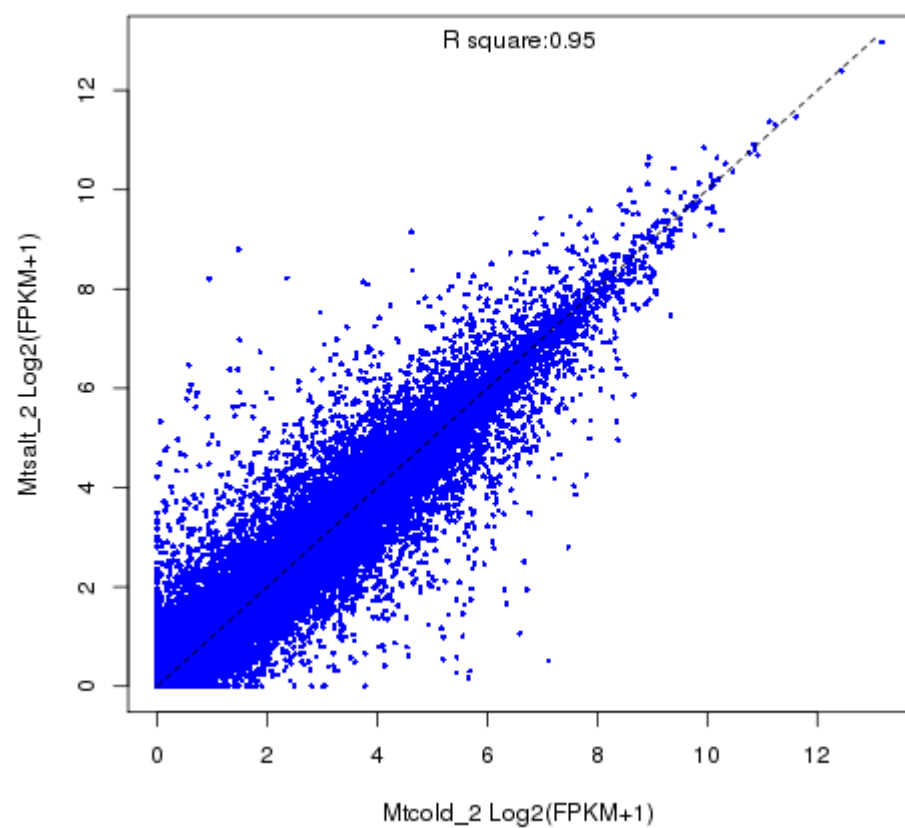

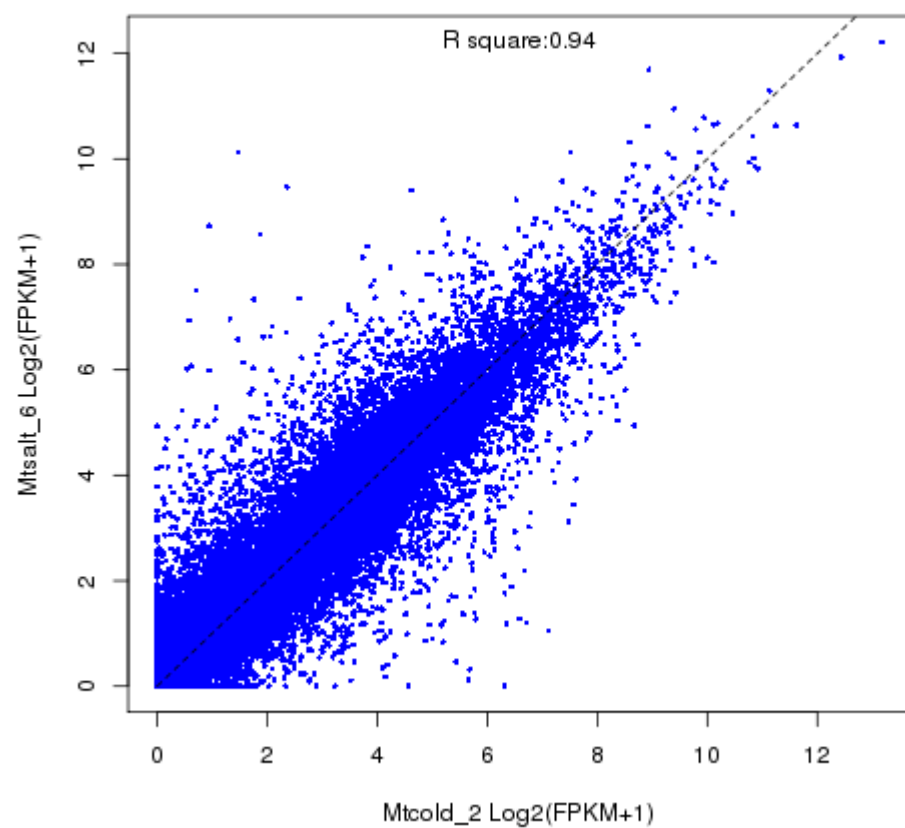

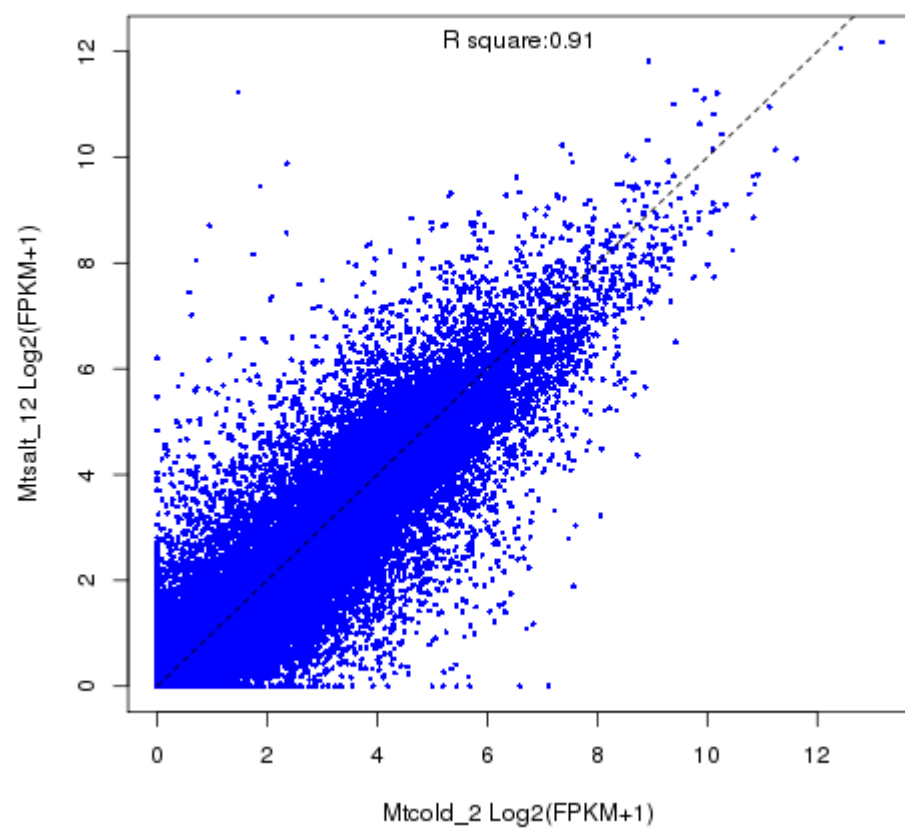

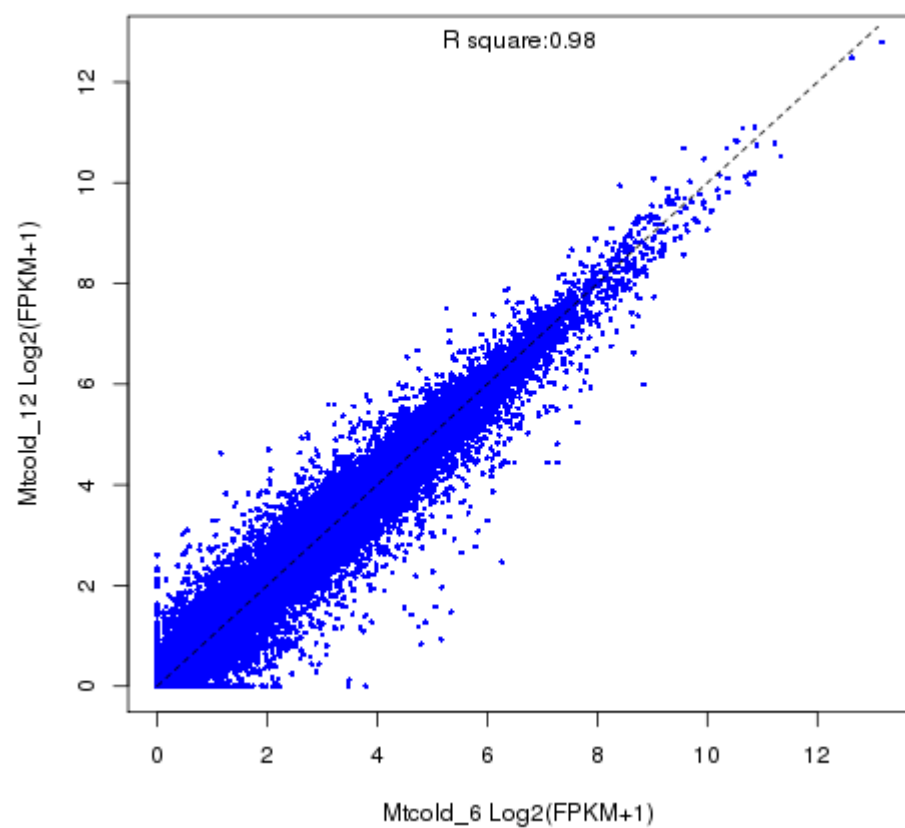

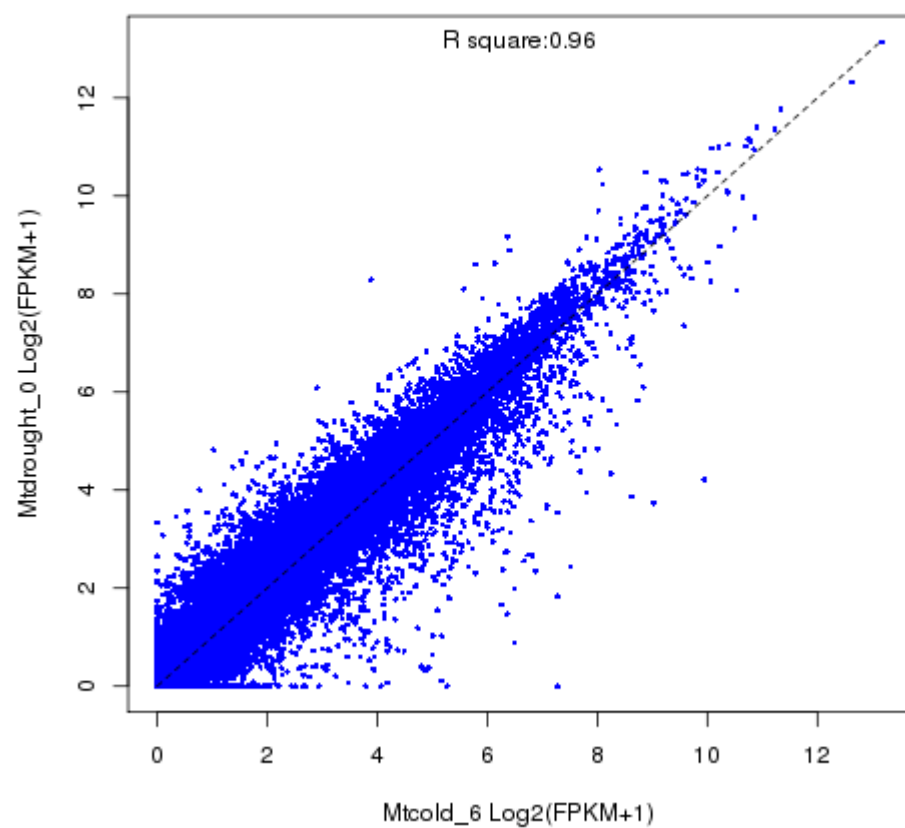

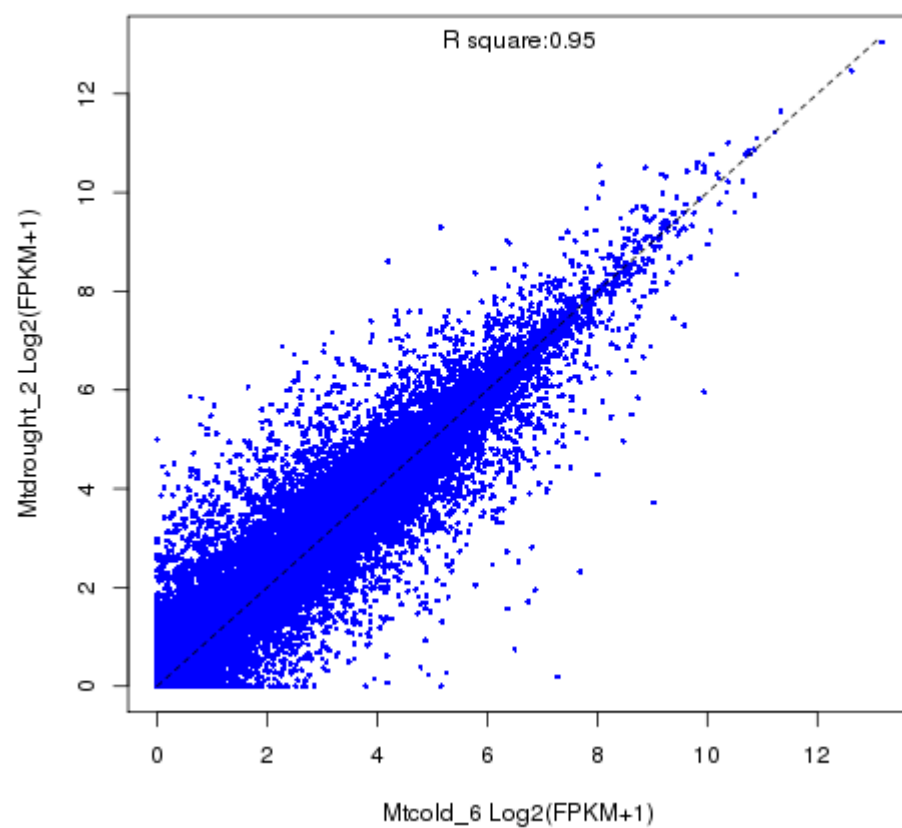

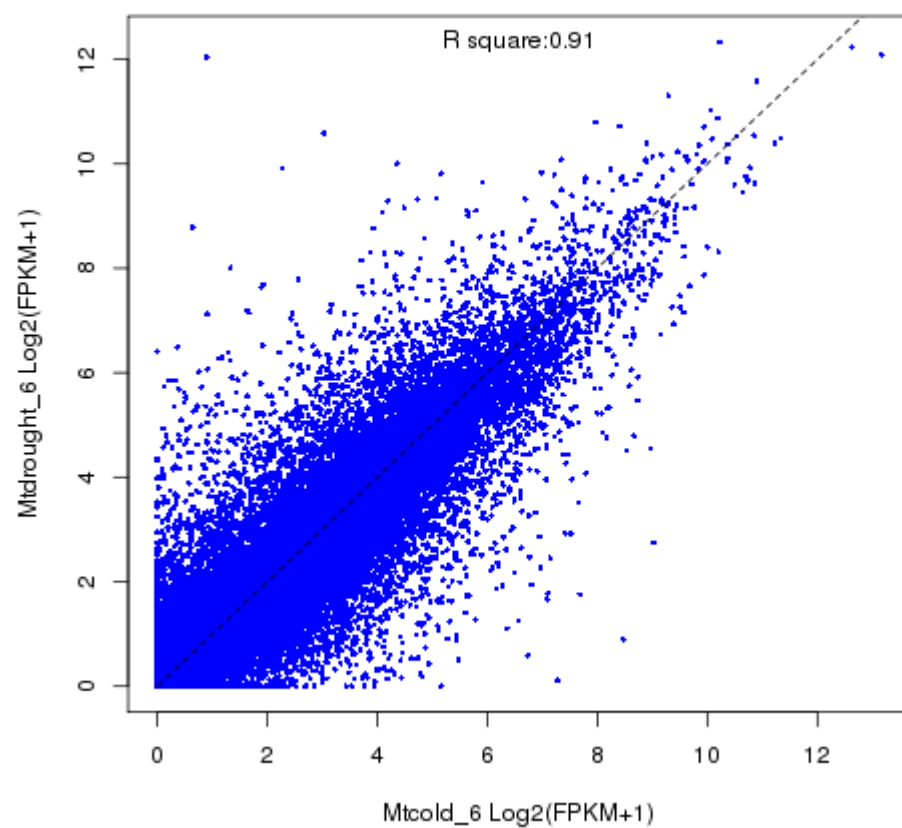

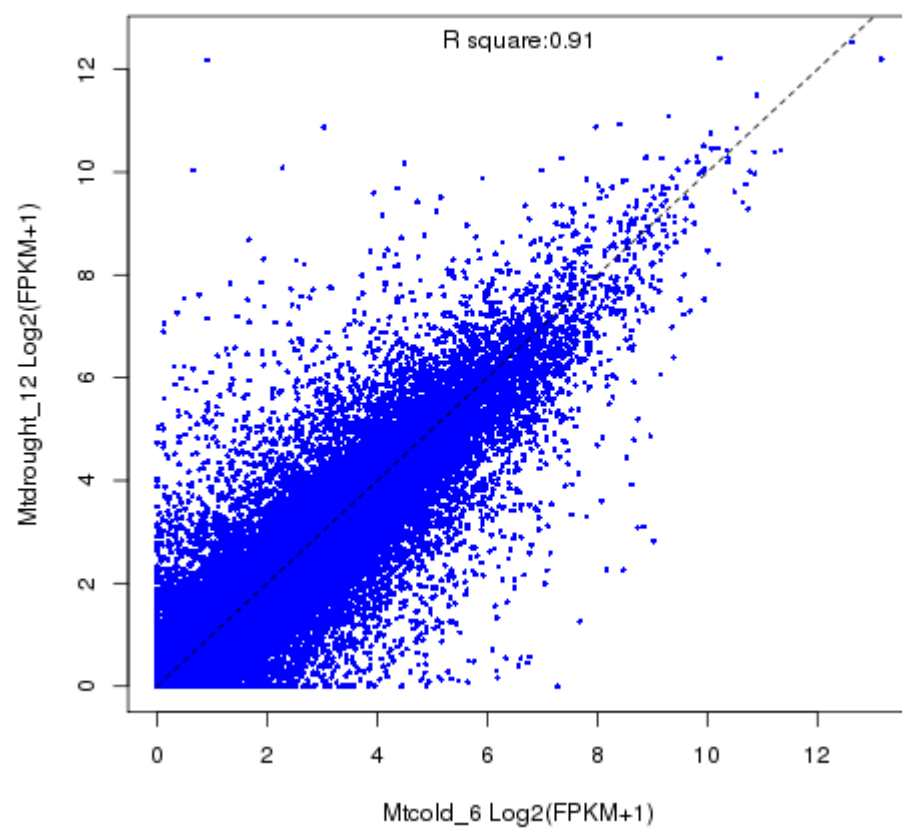

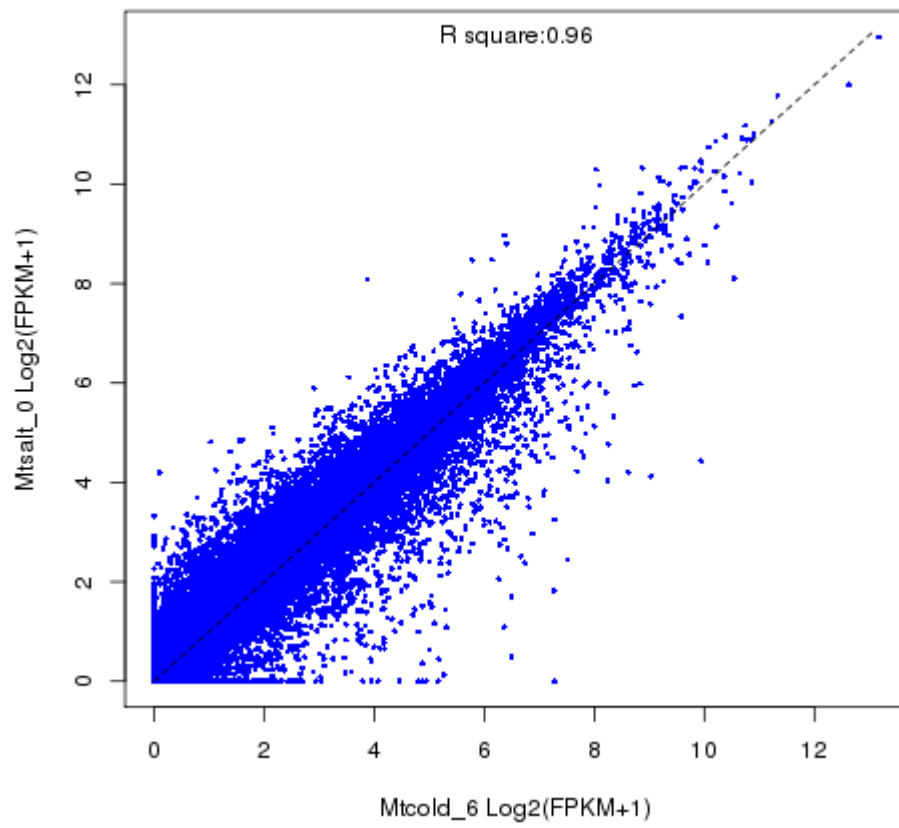

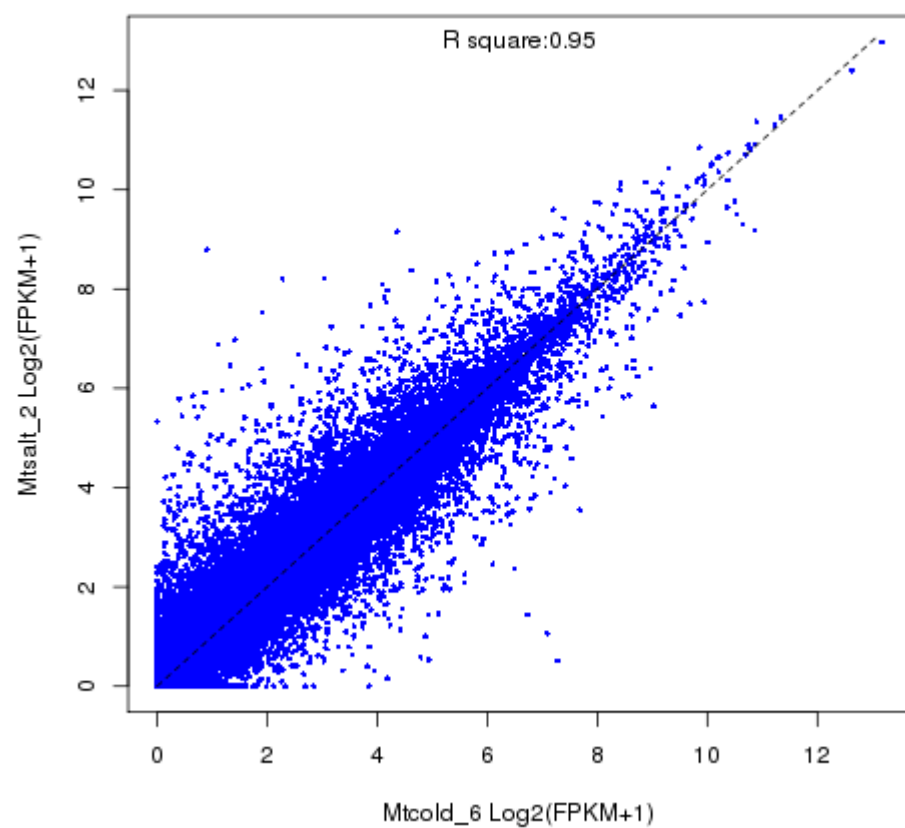

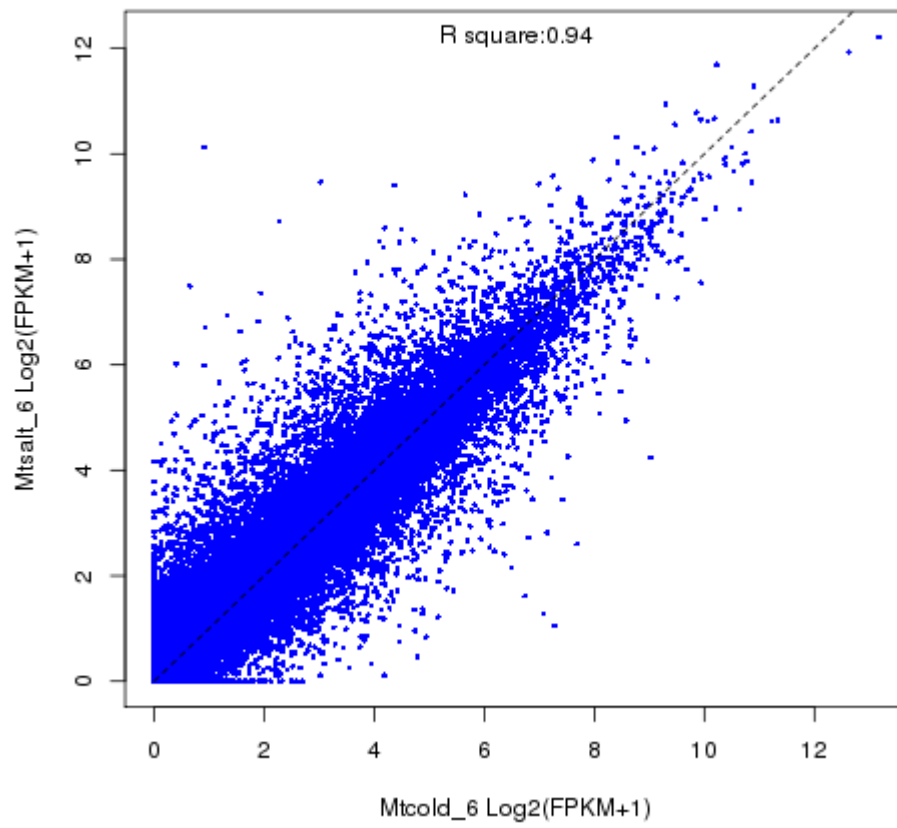

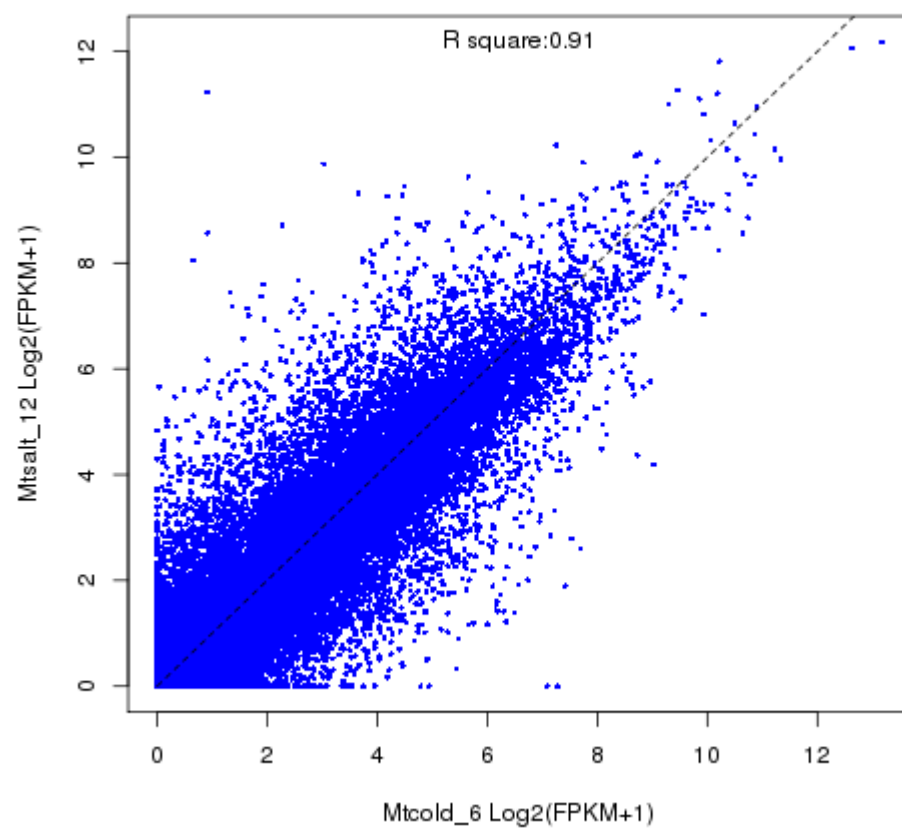

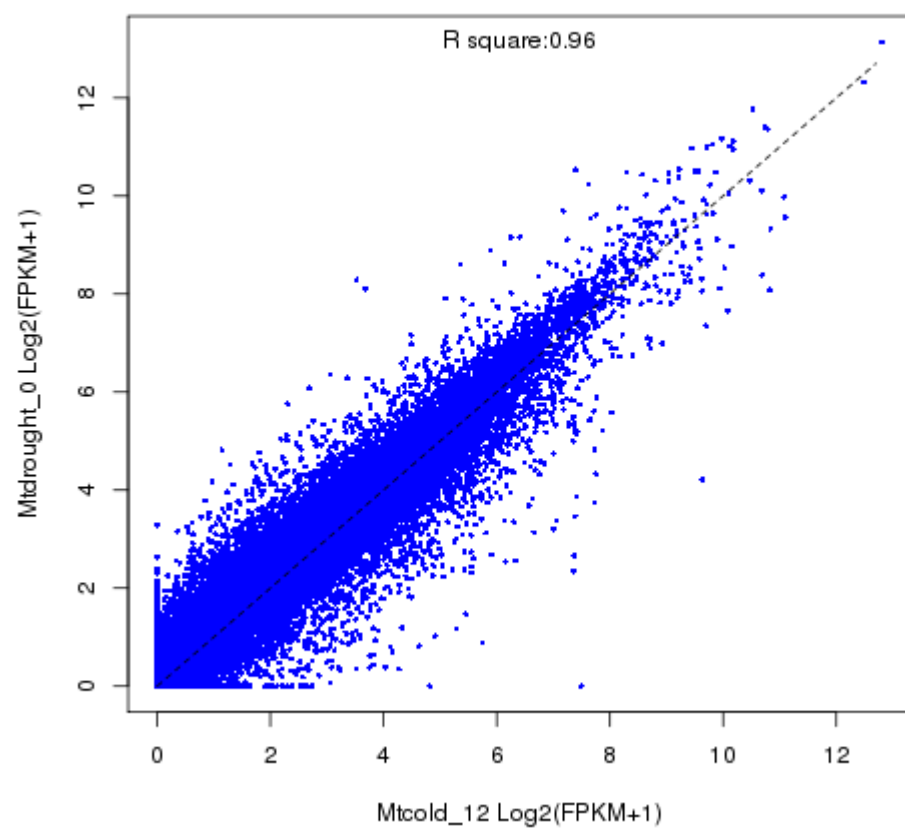

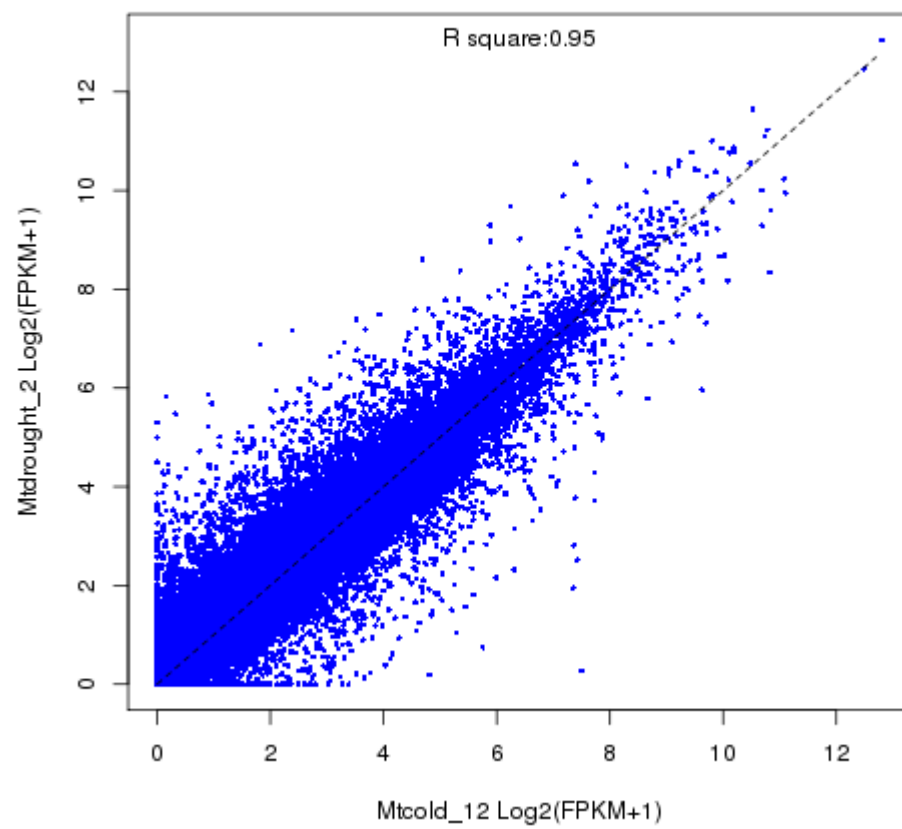

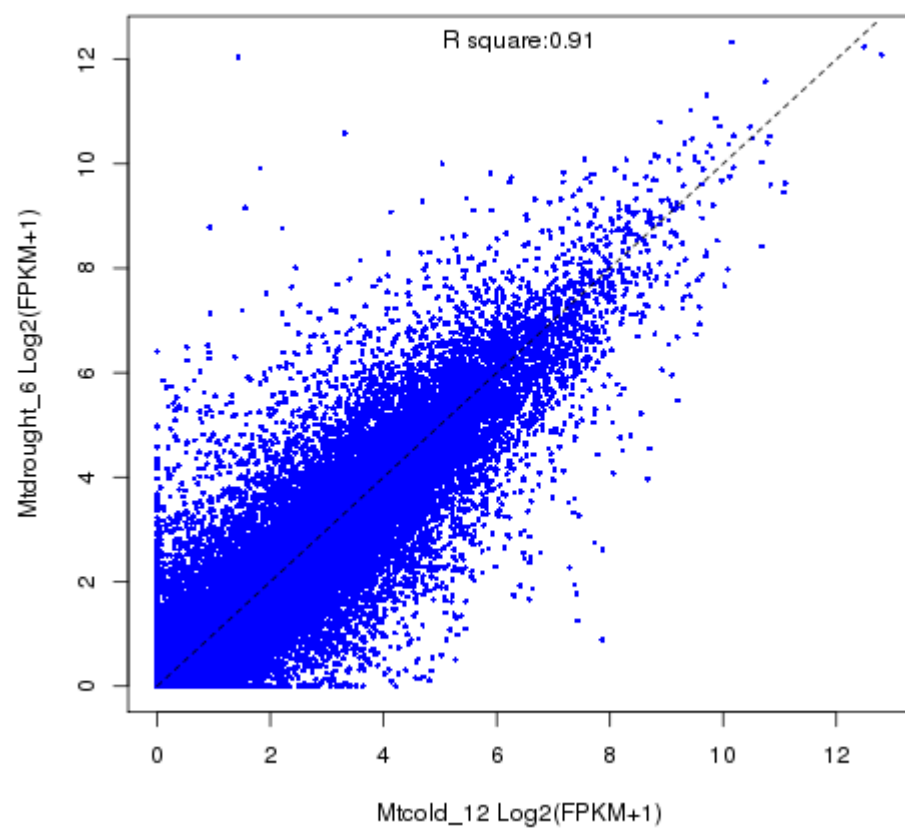

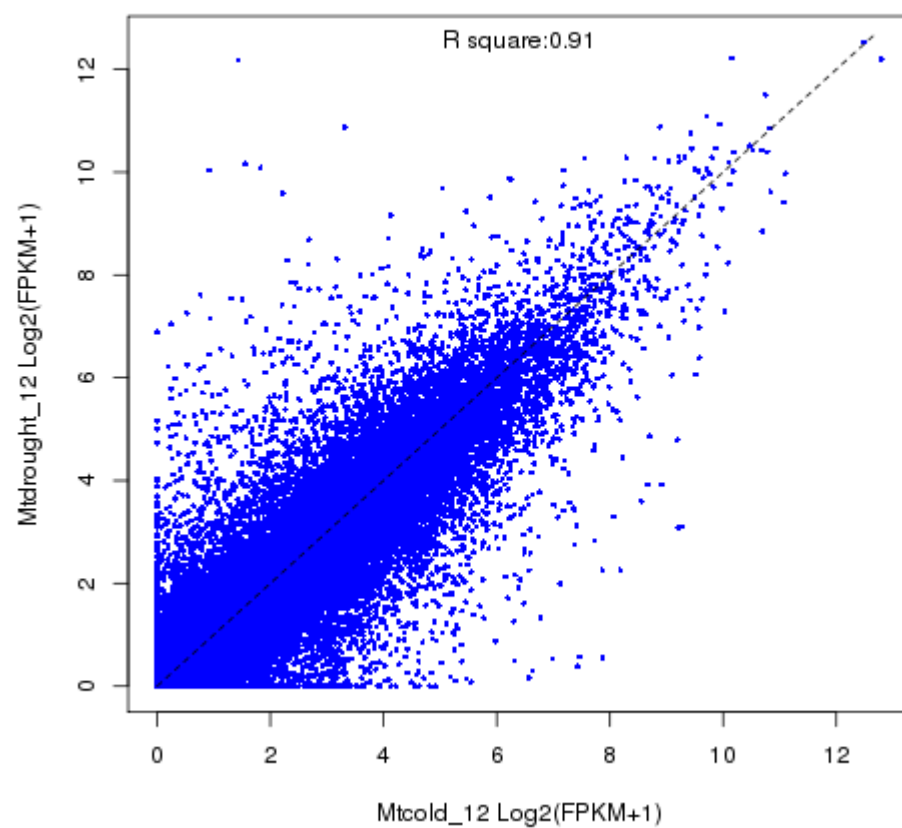

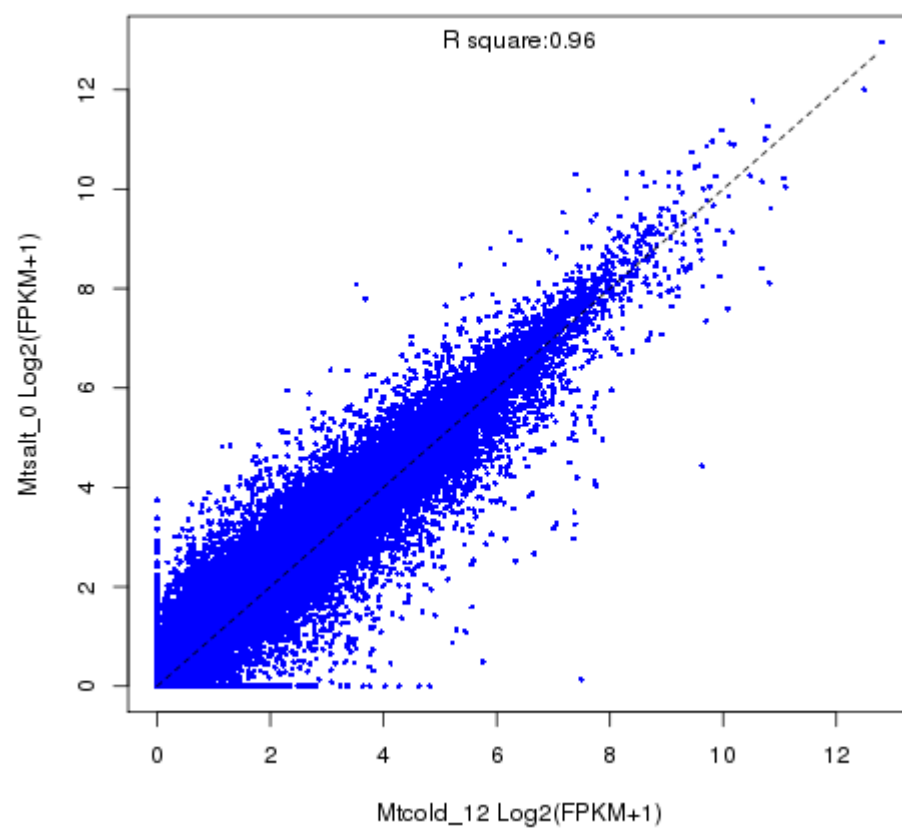

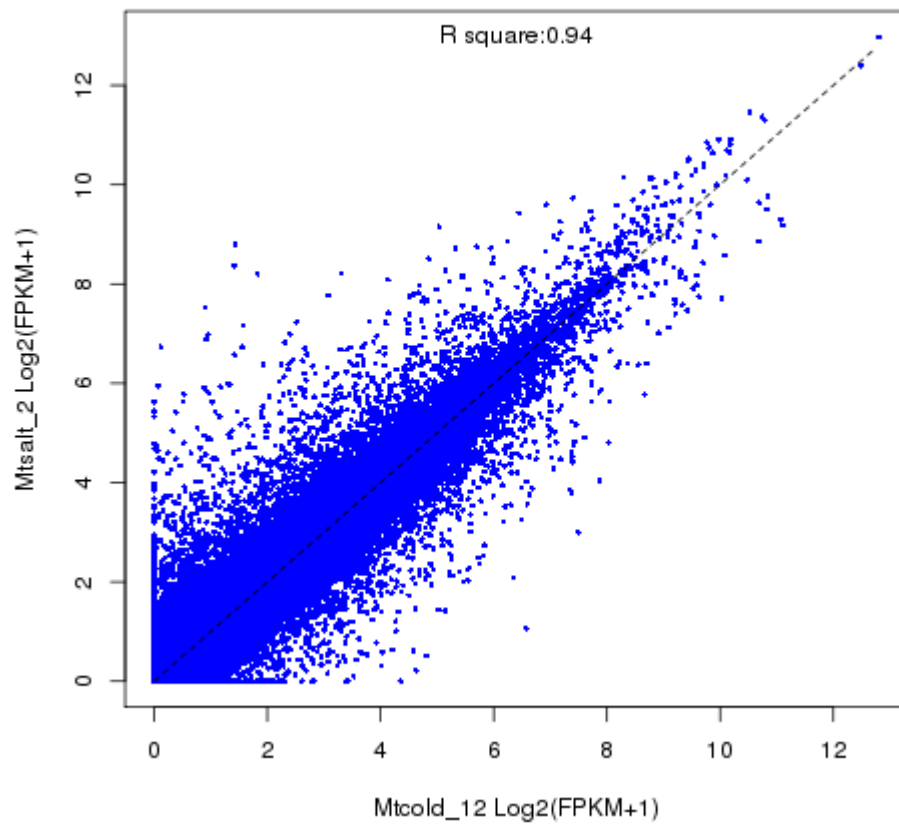

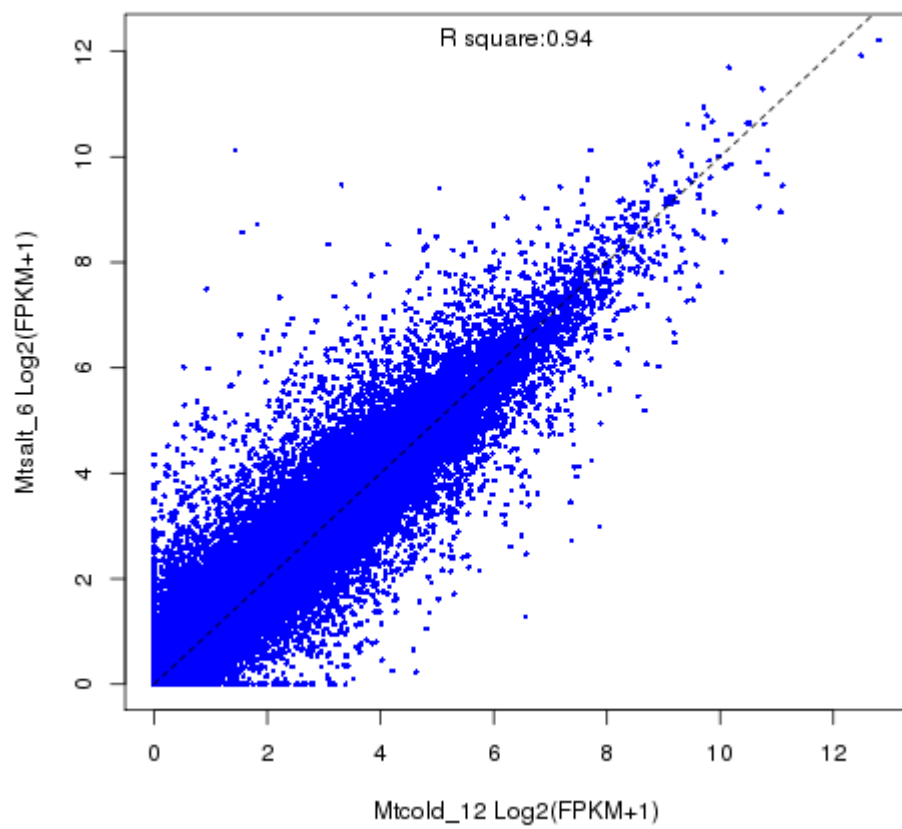

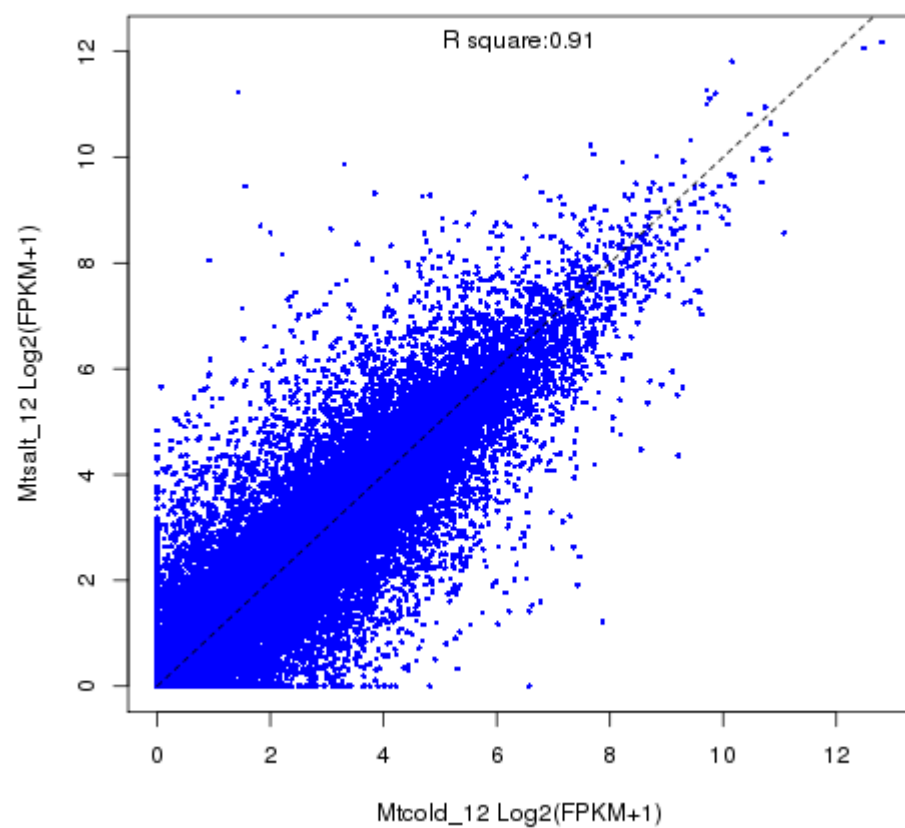

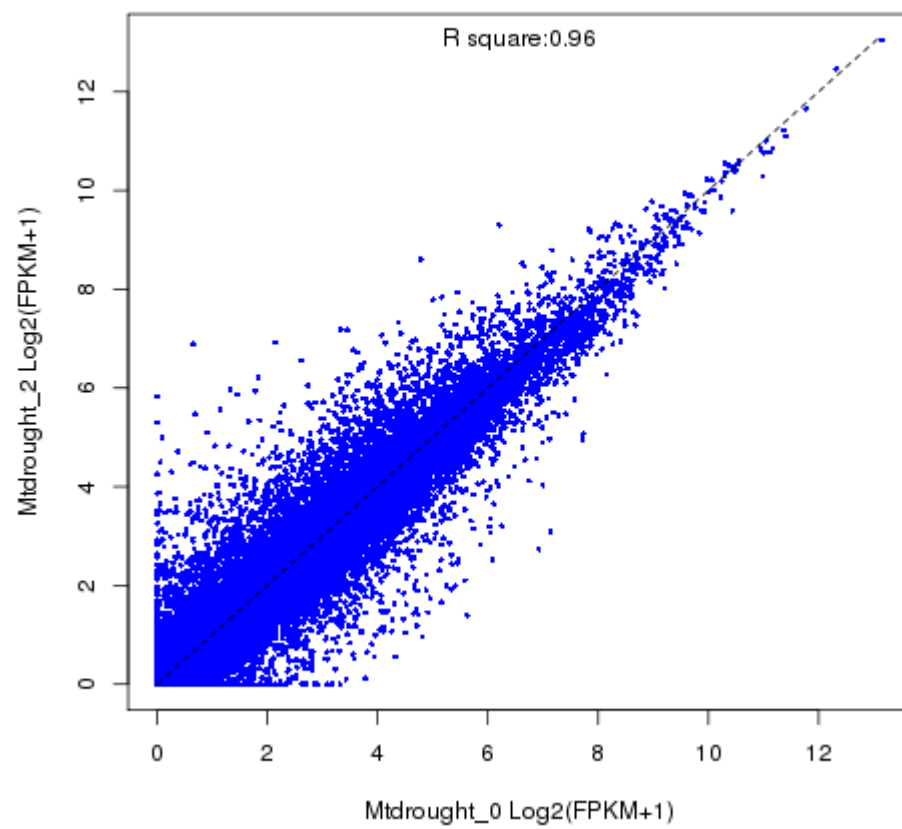

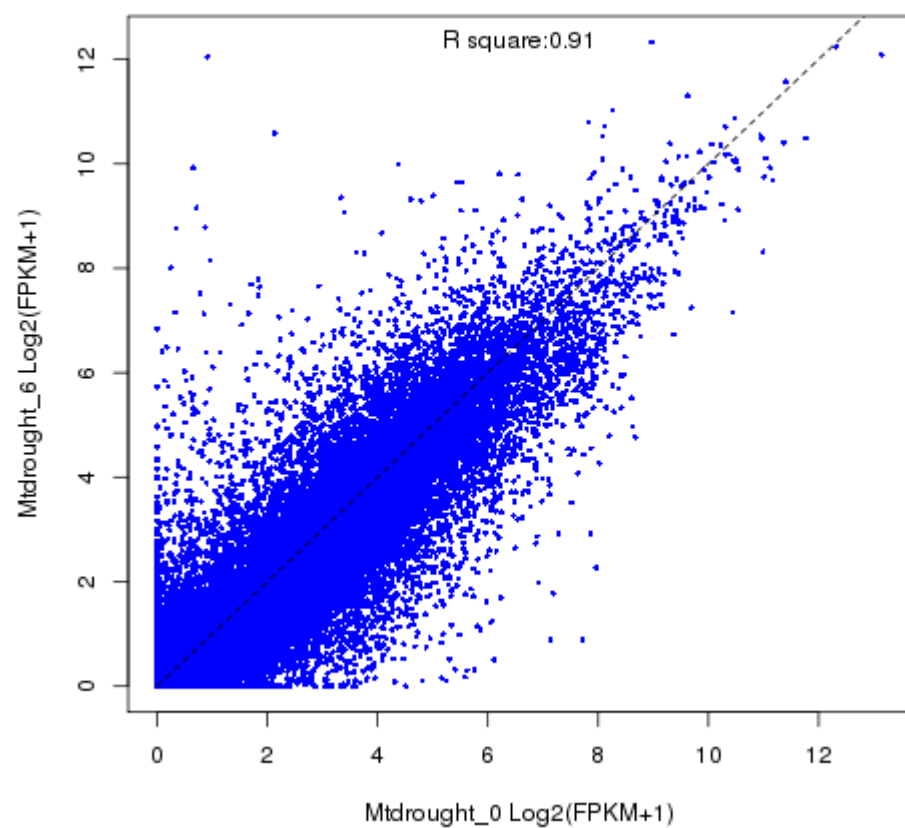

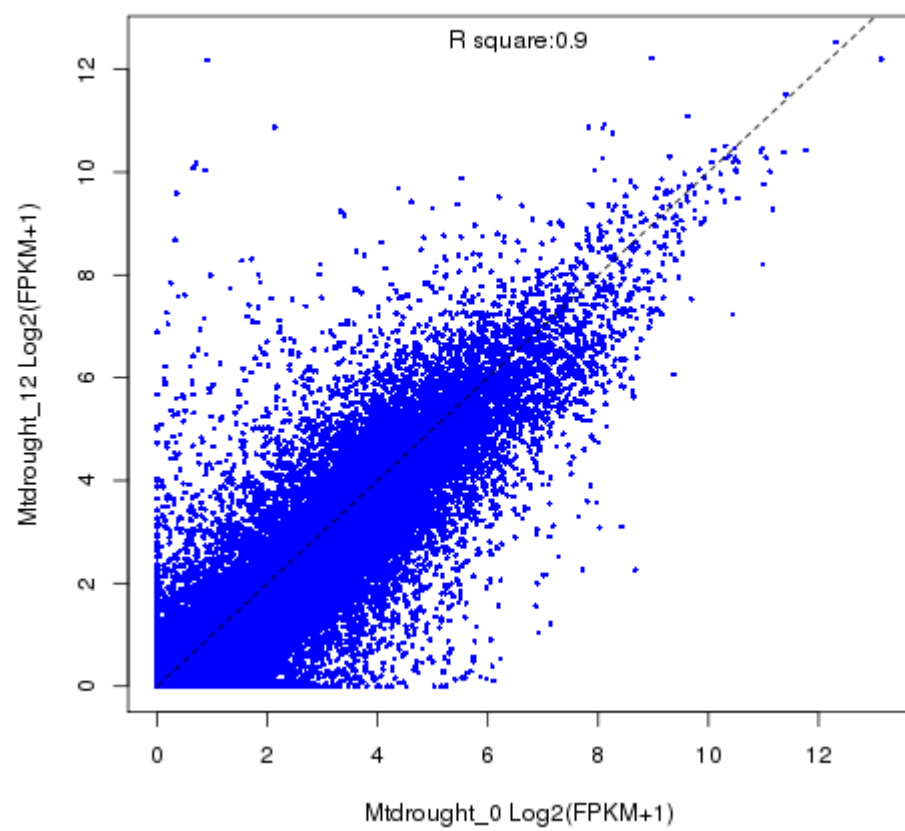

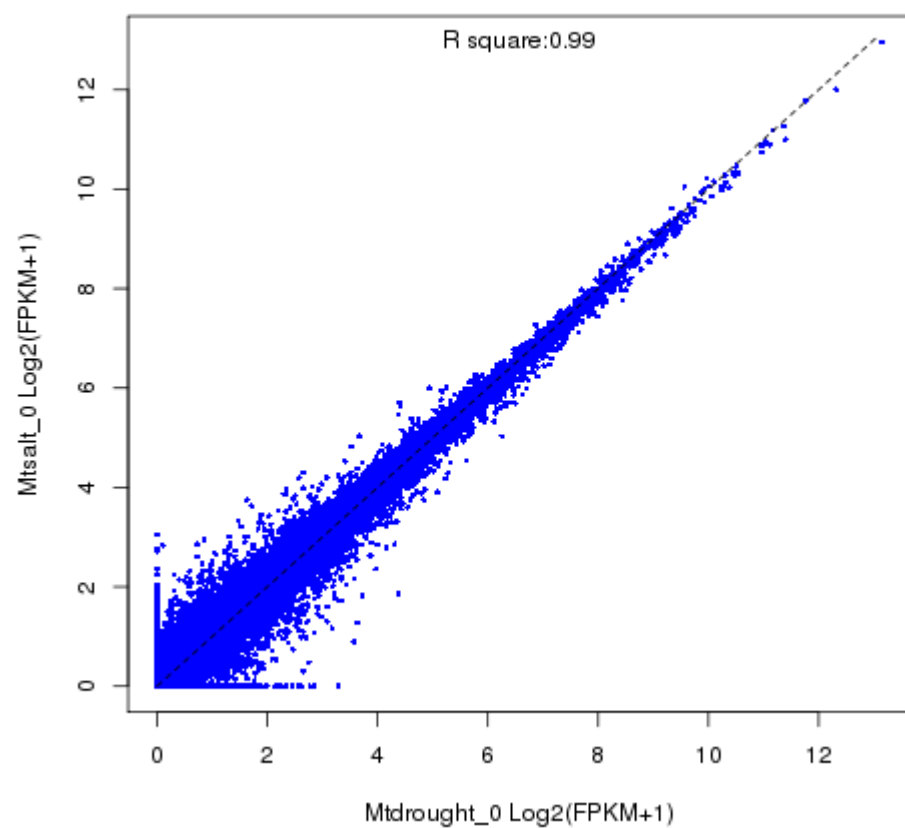

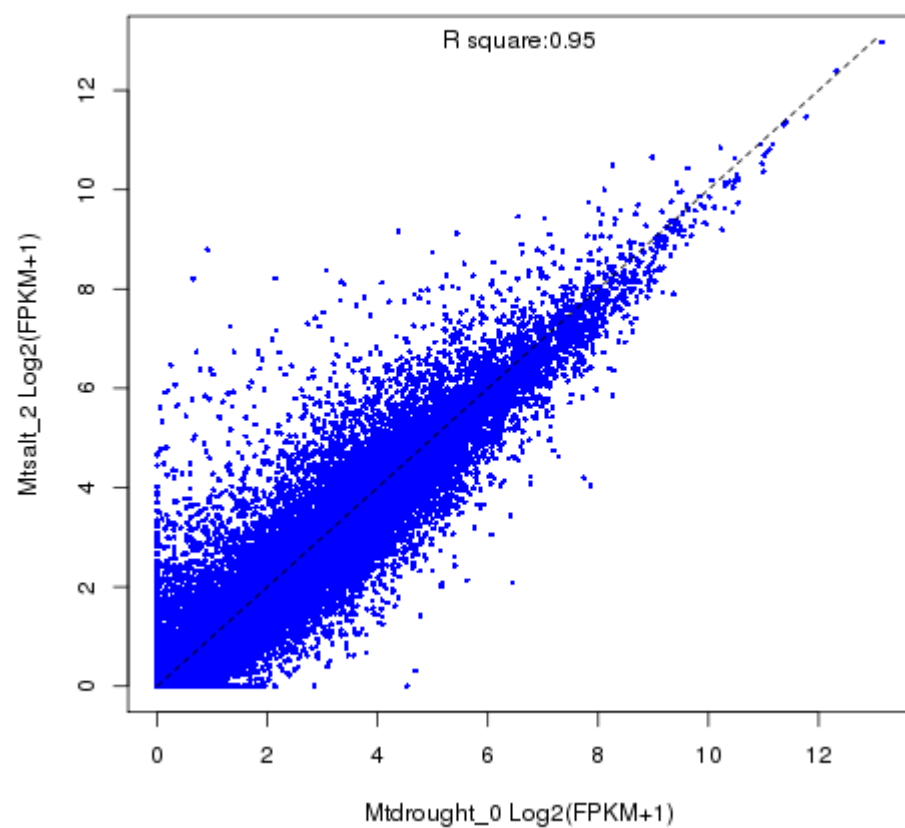

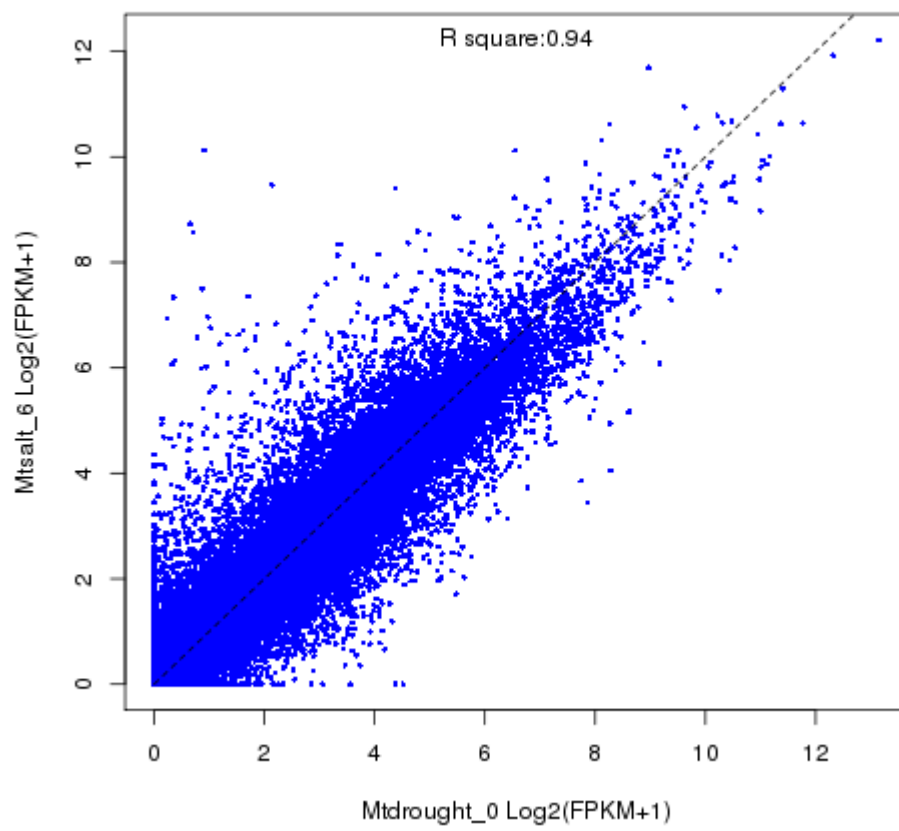

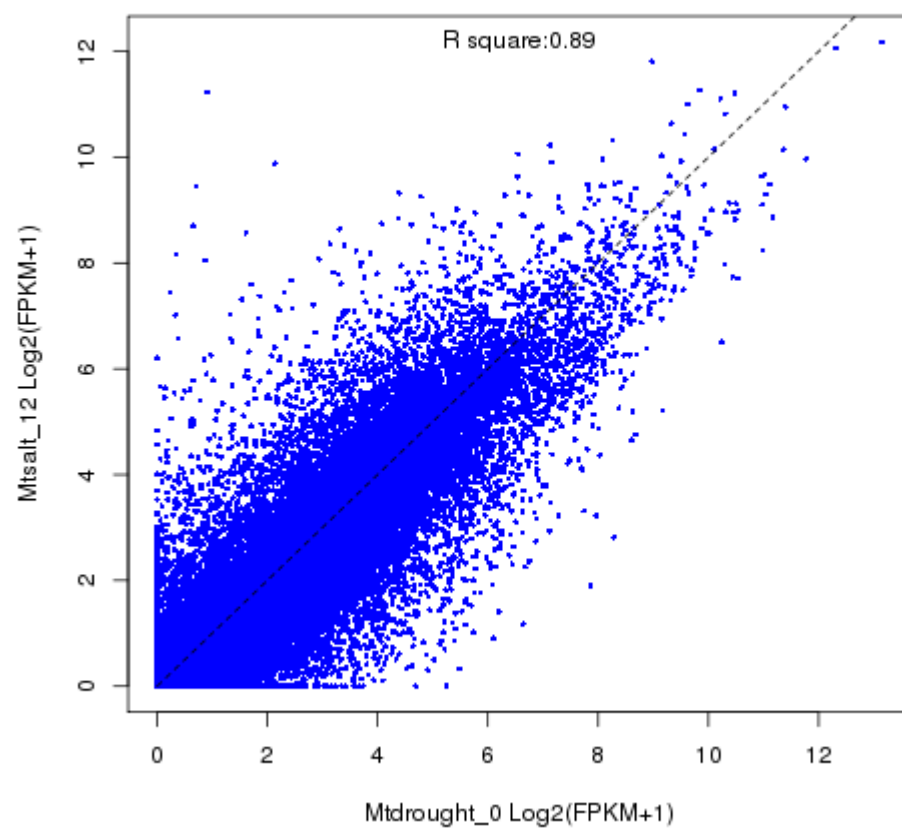

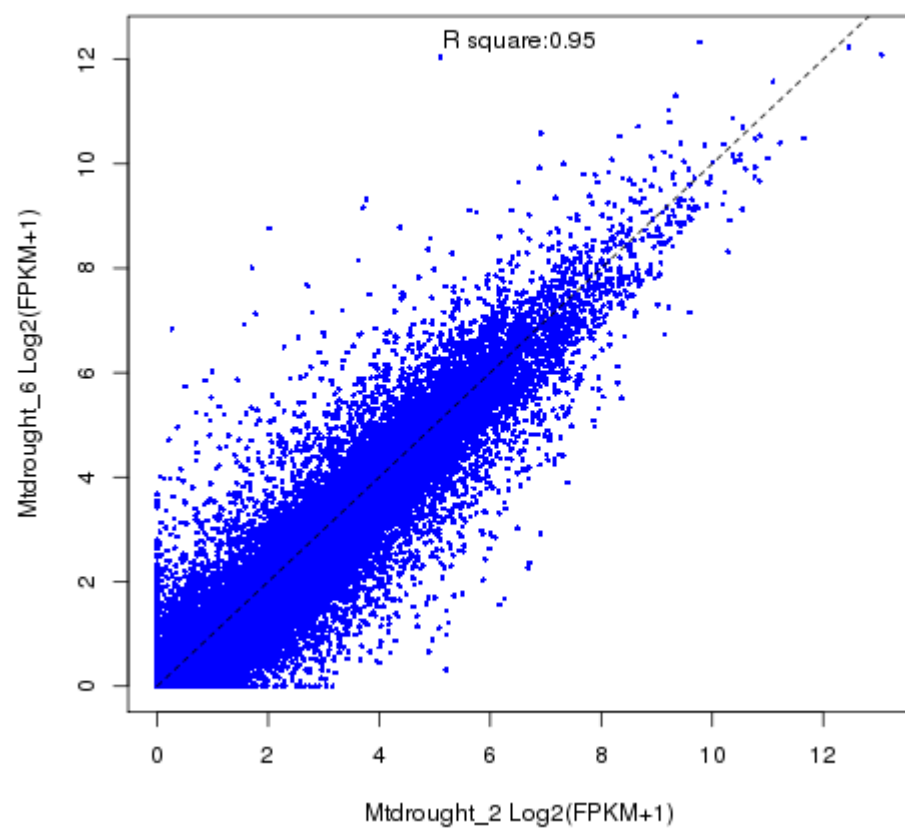

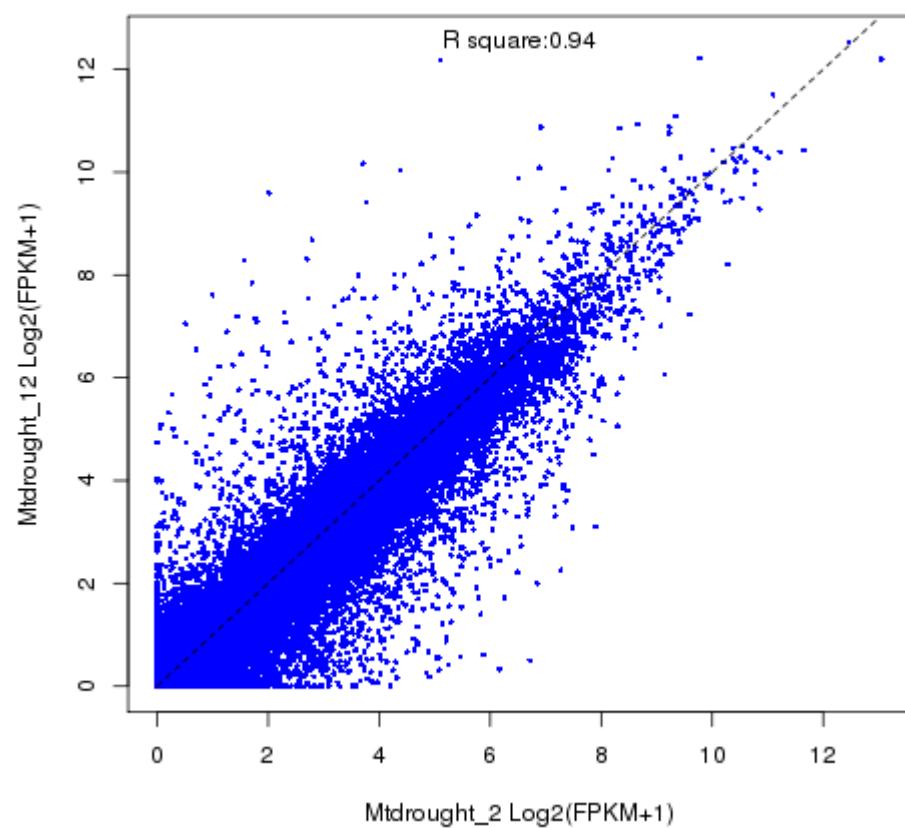

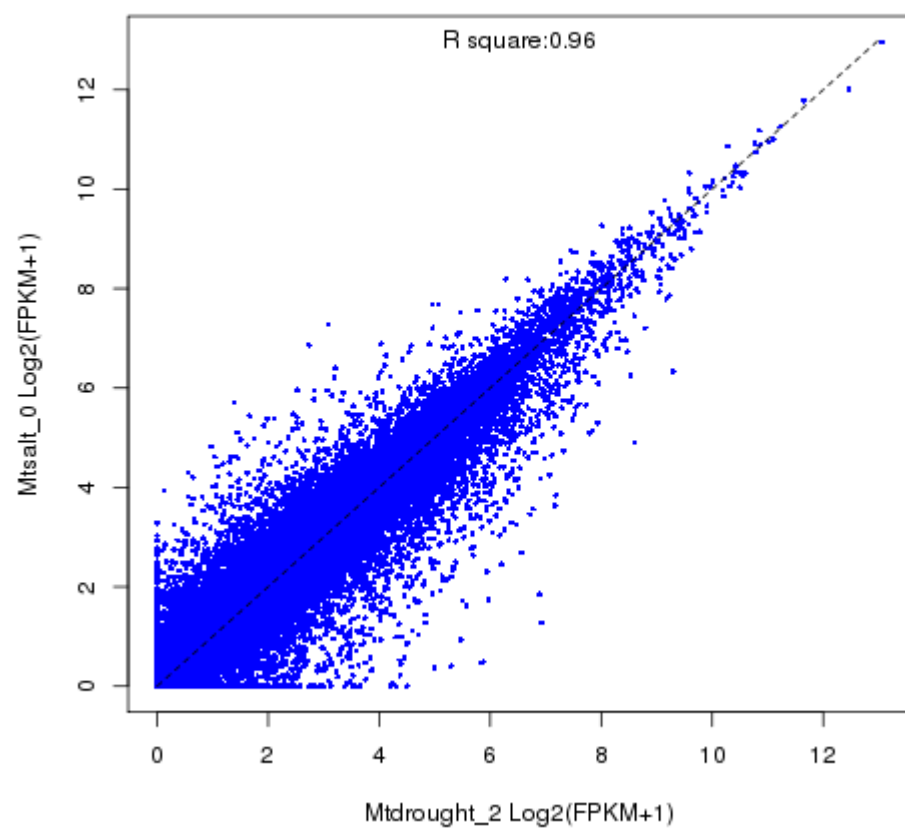

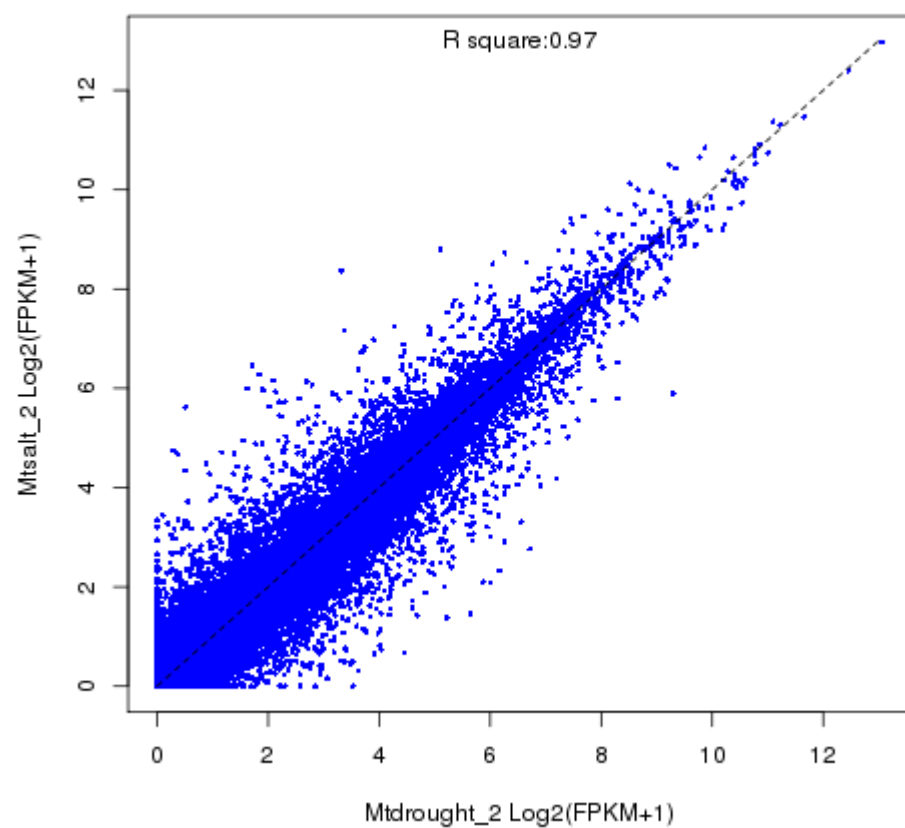

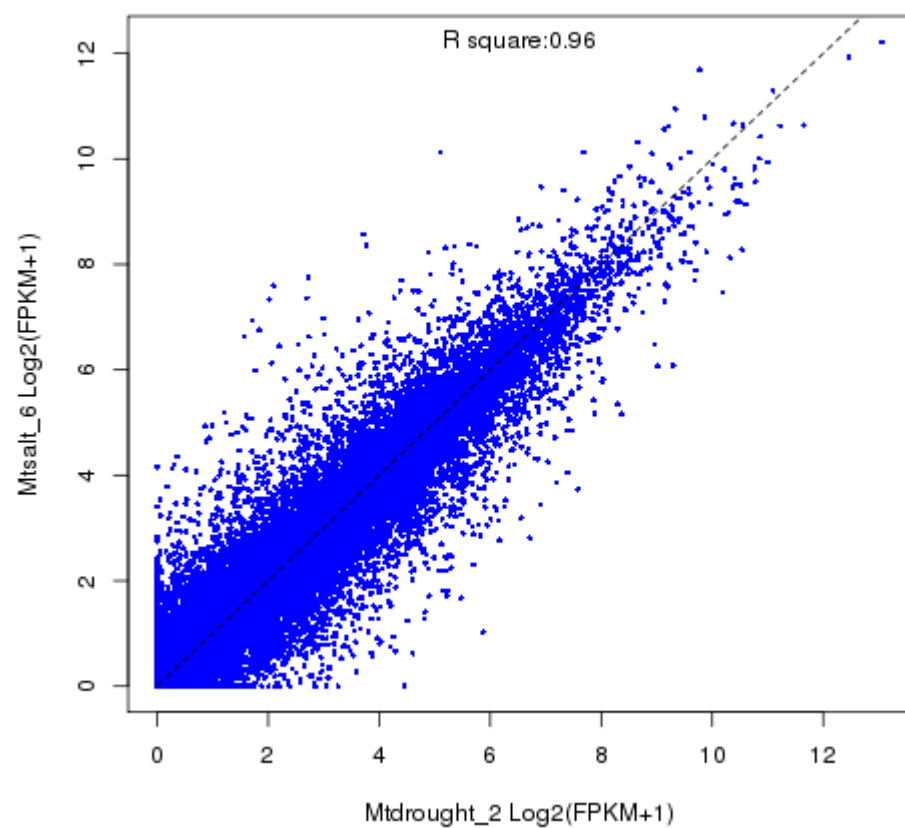

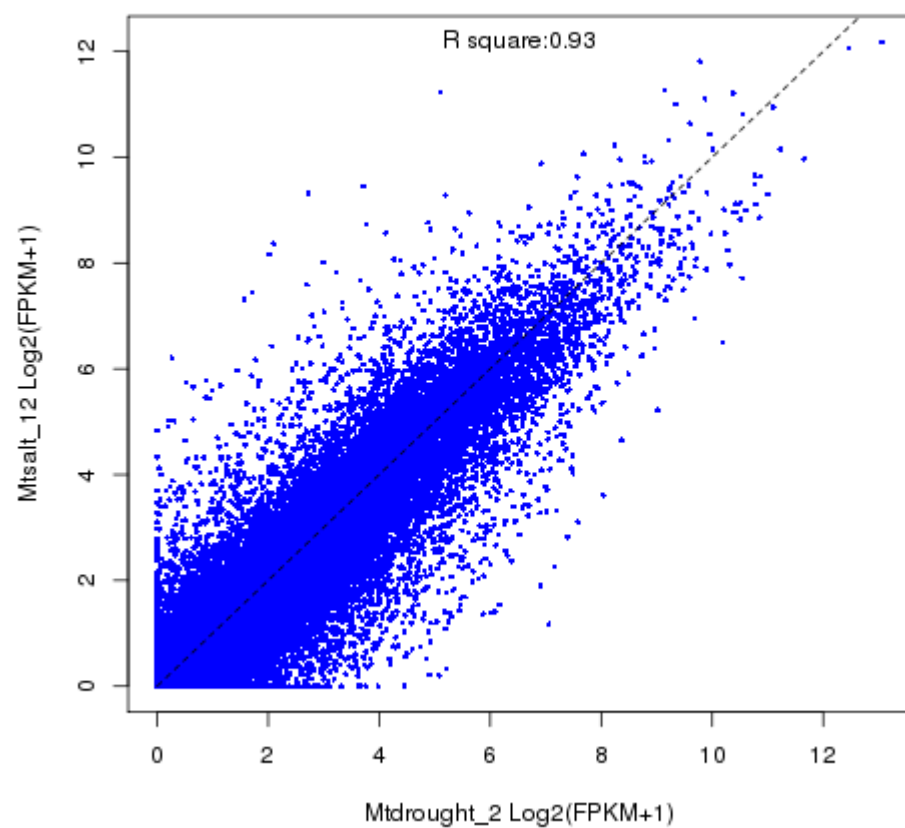

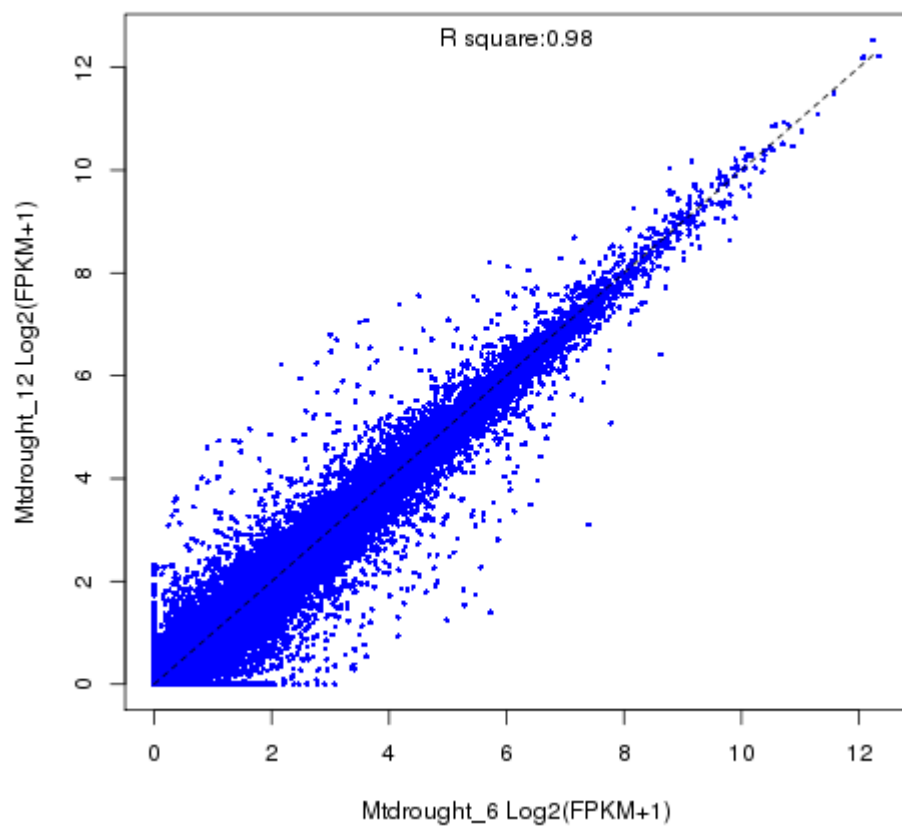

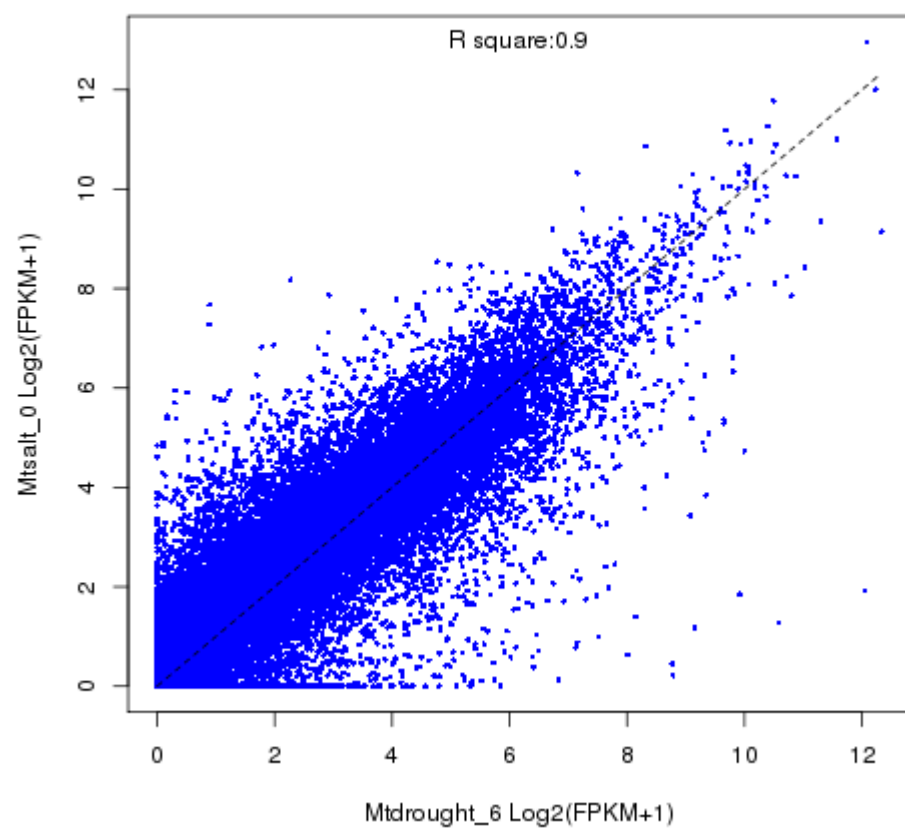

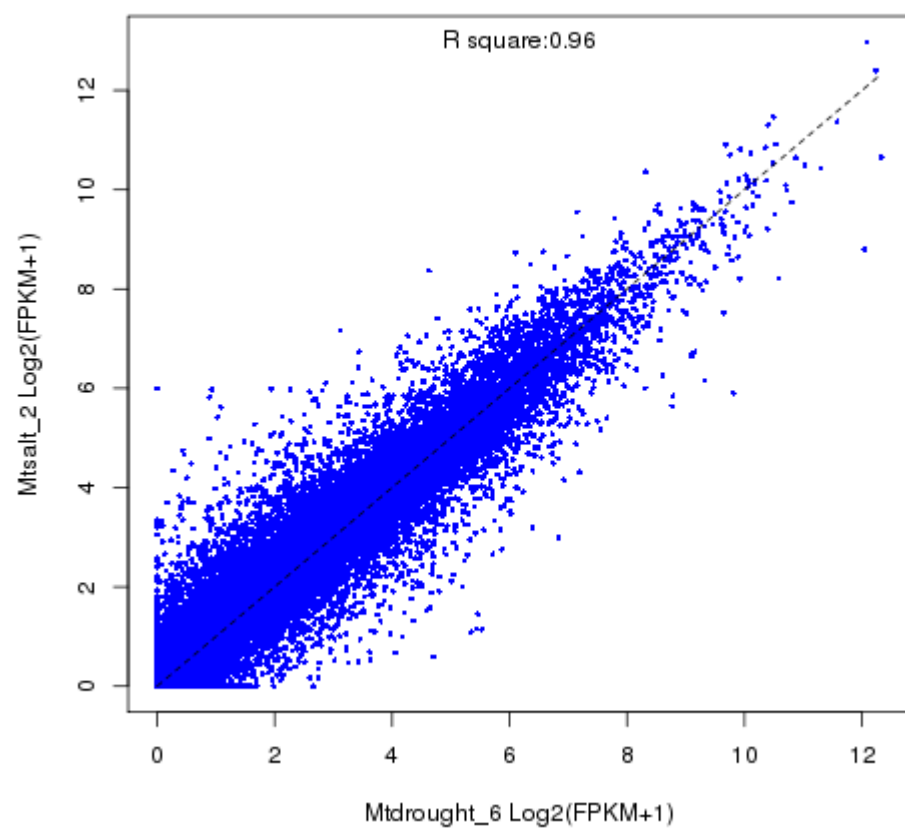

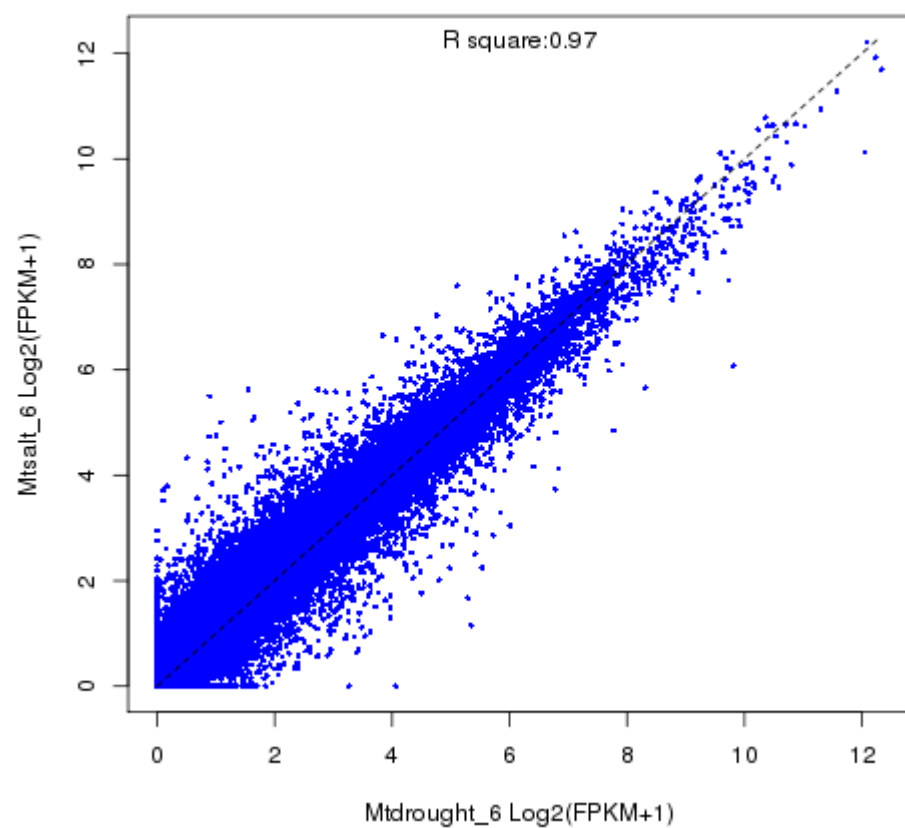

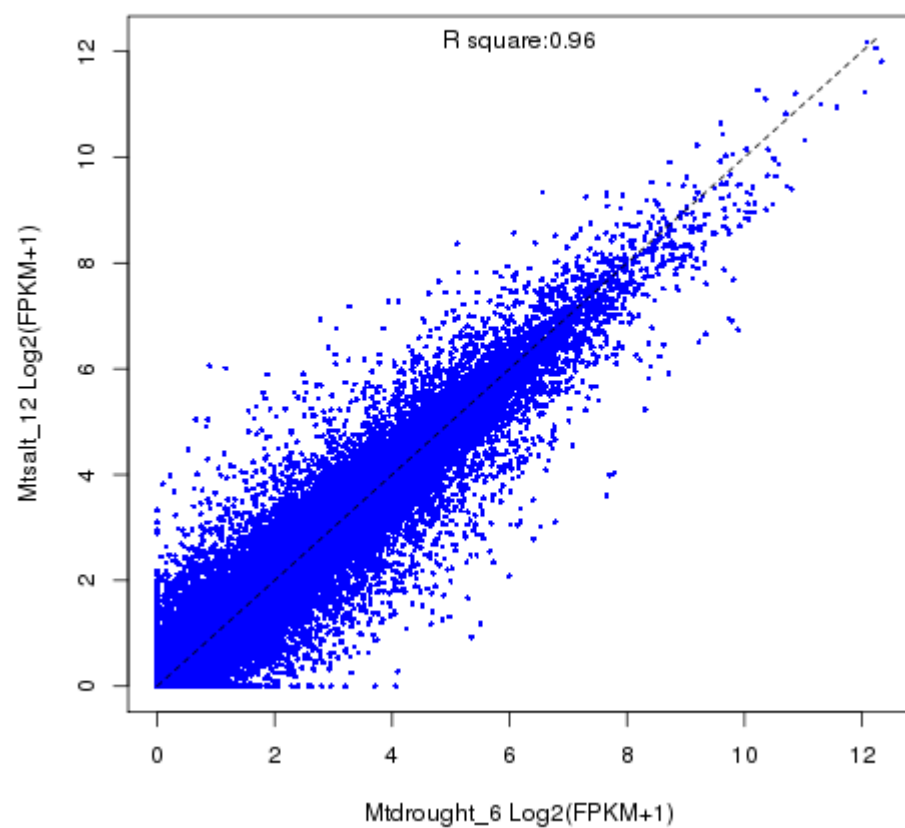

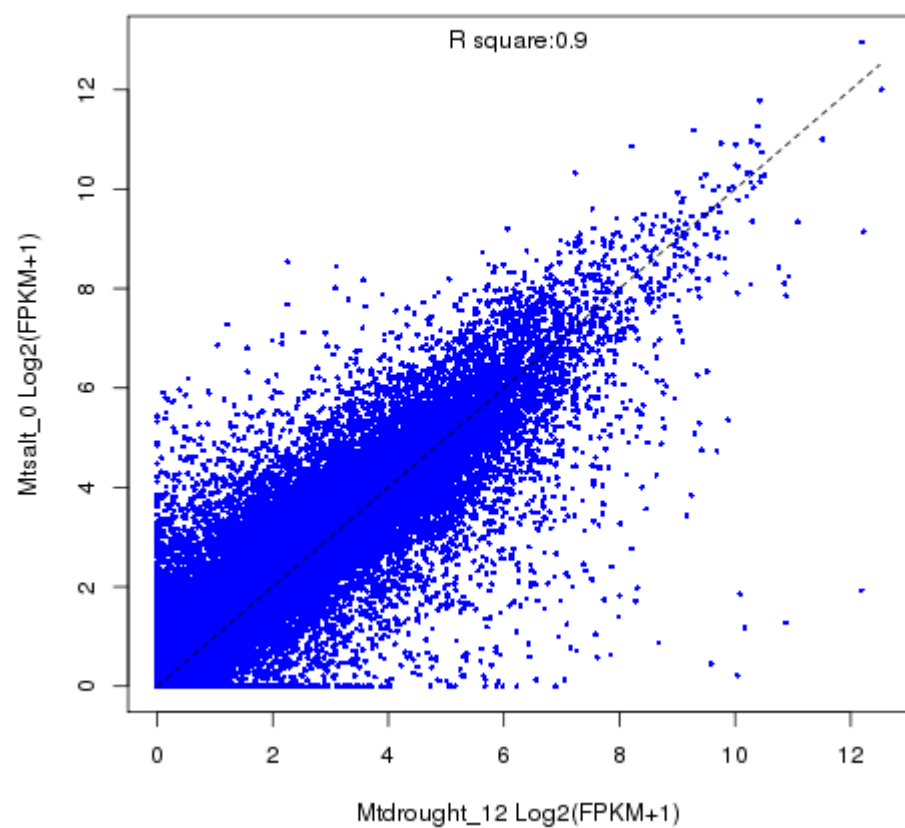

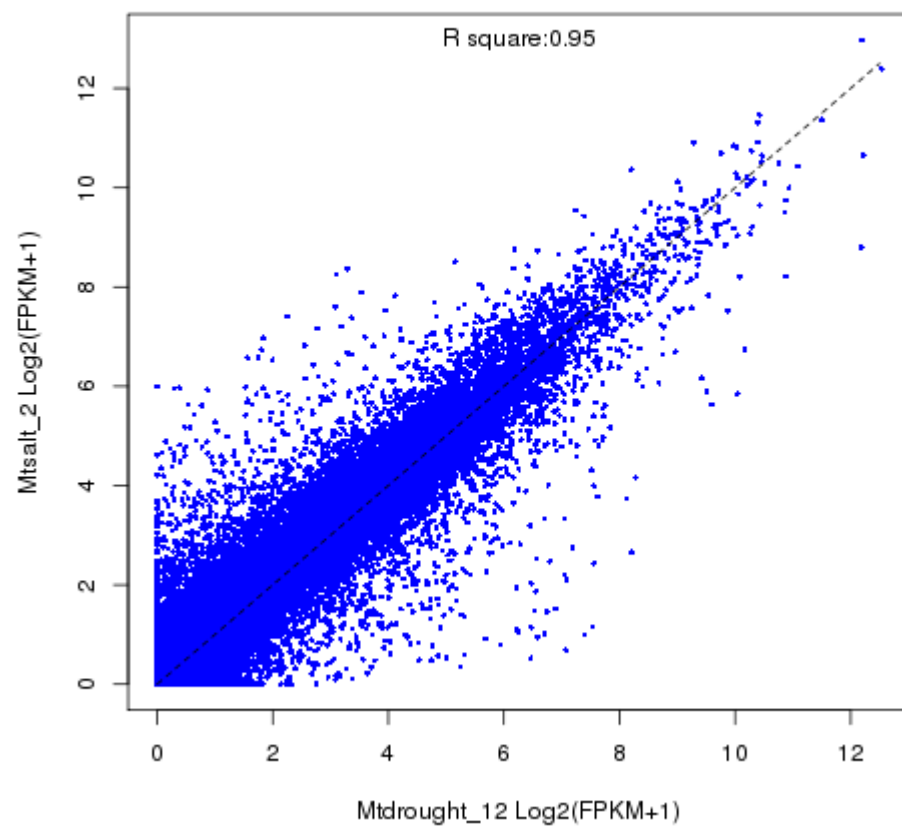

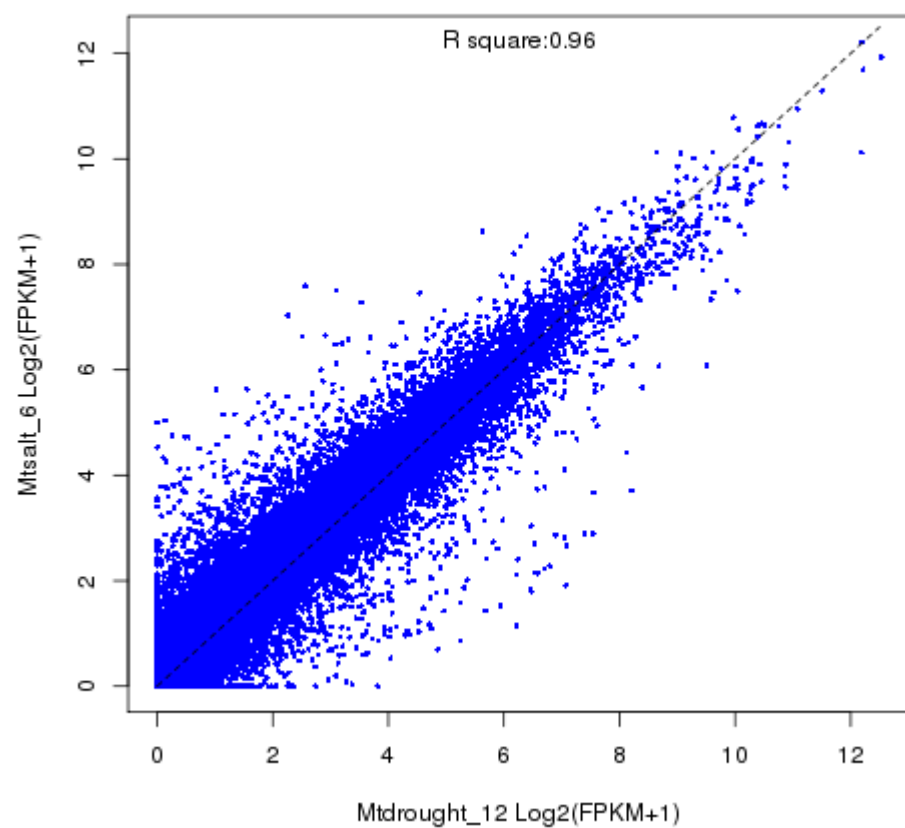

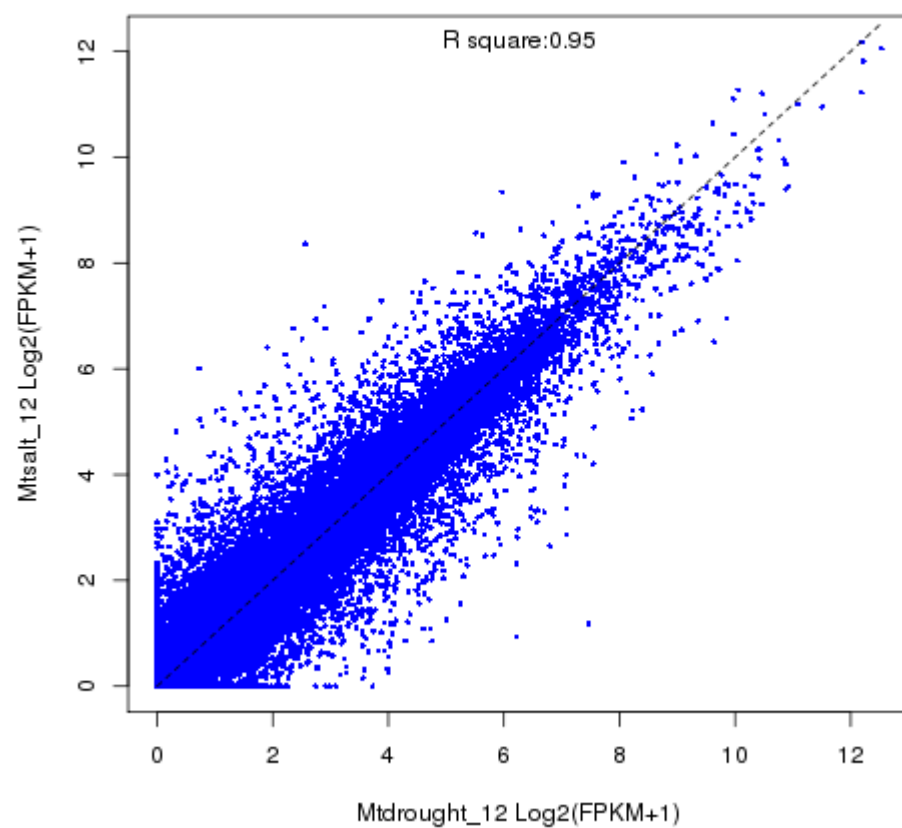

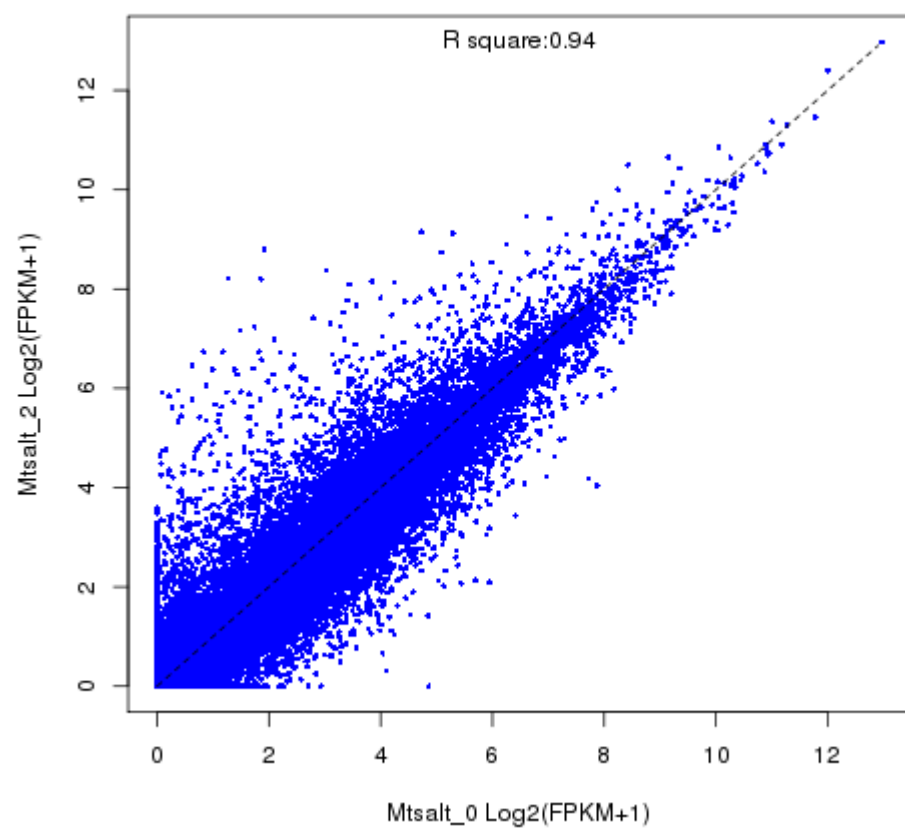

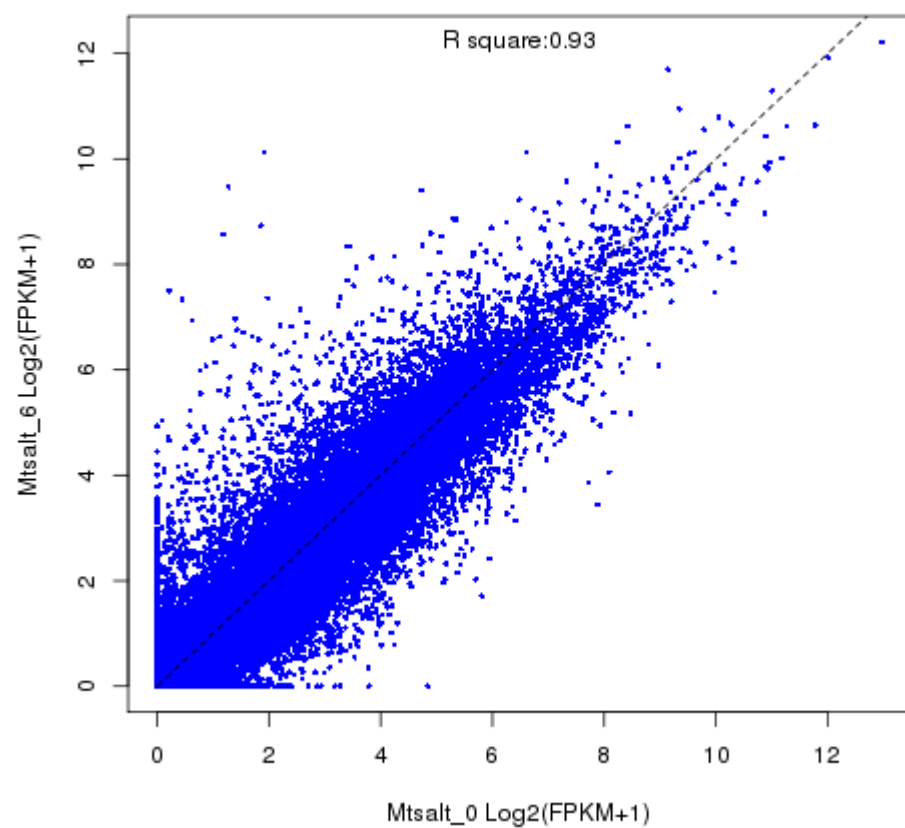

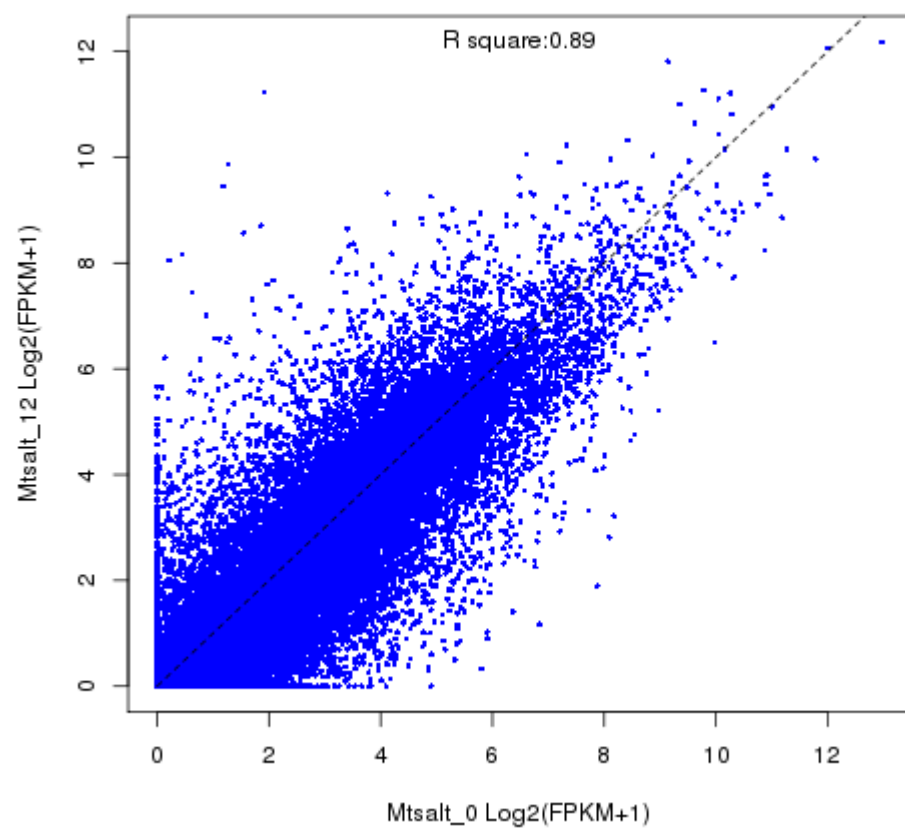

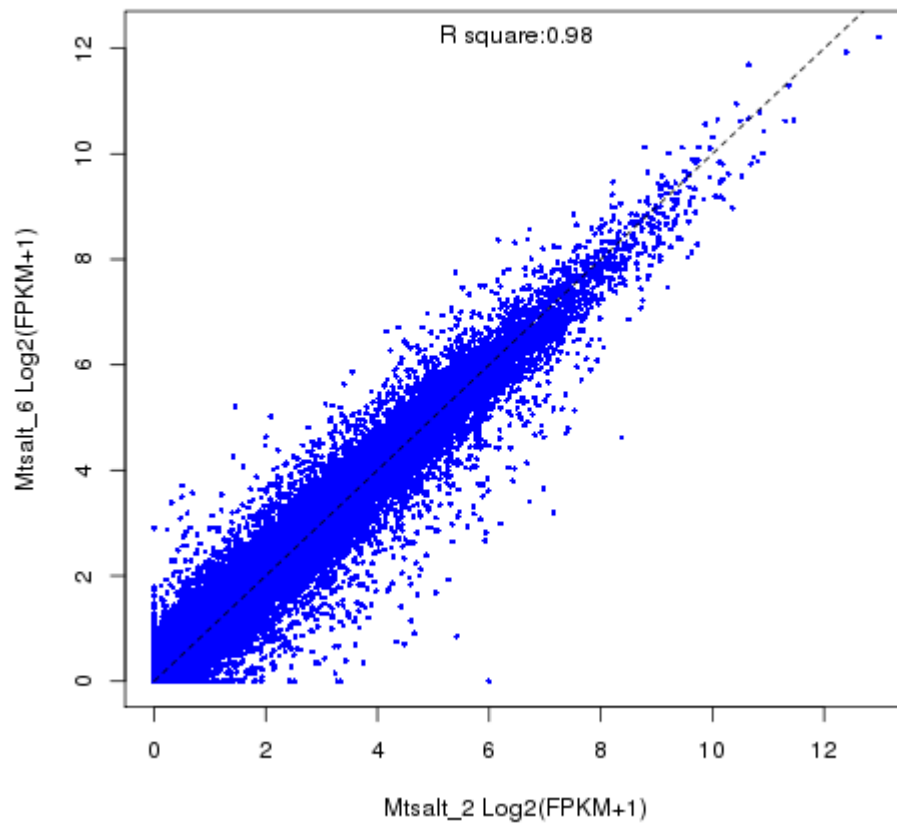

S3 Fig. Abundance of transcriptions between two samples

Supplement: S3 Fig — (PDF) [file pone.0182402.s003.pdf]
